# Structure–Activity Relationship Studies on 6-Chloro-1-phenylbenzazepines Leads to the Identification of a New Dopamine D1 Receptor Antagonist

**DOI:** 10.3390/molecules28166010

**Published:** 2023-08-11

**Authors:** Rajan Giri, Hari K. Namballa, Vishwashiv Emogaje, Wayne W. Harding

**Affiliations:** 1 Department of Chemistry, Hunter College, City University of New York, 695 Park Avenue, New York, NY 10065, USA; 2 Program in Chemistry, CUNY Graduate Center, 365 5th Avenue, New York, NY 10016, USA; 3 Program in Biochemistry, CUNY Graduate Center, 365 5th Avenue, New York, NY 10016, USA

**Keywords:** benzazepine, D1R, D5R, antagonist, dopamine

## Abstract

The 1-phenylbenzazepine template has yielded a number of D1R-like ligands, which, though useful as pharmacological tools, have significant drawbacks in terms of selectivity versus D5R as well as pharmacokinetic behavior. A number of 1-phenylbenzazepines contain a 6-chloro functional group, but extensive SAR studies around the 6-chloro-1-phenylbenzazepine framework have not been reported in the literature. To further understand the tolerance of the 6-chloro-1-phenylbenzazepine template for various substituent groups towards affinity and selectivity at D1R, we synthesized two series of analogs with structural variations at the C-7, C-8, *N*-3, C-3′ and C-4′ positions. The series 2 analogs differed from series 1 analogs in possessing a nitrogenated functionality at C-8 and lacked a C-4′ substituent, but were otherwise similar. Analogs were assessed for affinity at D1R, D2R and D5R. For both series, we found that the analogs lacked affinity for D2R and showed modest D1R versus D5R selectivity. For series 1 analogs, an *N*-3 methyl substituent group was better tolerated than *N*-H or an *N*-3 allyl substituent. The C-8 position appears to be tolerant of amino and methanesulfonamide substituents for high D1R affinity, but C-8 amides displayed low to moderate D1R affinities. A C-3′ methyl substituent appeared to be critical for the D1R affinity of some analogs, but the C-4′ substituents tried (hydroxy and methoxy; series 1) did not result in any significant boost in D1R affinity. Compound **15a** was the most potent and selective D1R ligand identified from these studies (K_i_ at D1R = 30 nM; 6-fold selectivity versus D5R). Further functional activity assessments indicate that **15a** functions as a D1R antagonist towards cAMP-mediated signaling. The predicted drug-like properties of **15a** are encouraging for further pharmacological assessments on the compound.

## 1. Introduction

The endogenous neurochemical dopamine is involved in various physiological processes in both the central and peripheral nervous systems, including the regulation of movement, emotion, blood pressure and cognition [1,2,3,4,5,6,7,8,9,10]. Its effects are mediated through interaction with five dopaminergic G-protein-coupled receptors [1,11]. These receptors are categorized into two families: the “D1R-like” and “D2R-like” families, based on their structural similarities and associated pharmacological effects. The D1R-like family consists of D1R (dopamine D1 receptor) and D5R (dopamine D5 receptor), while the D2R-like family includes D2R (dopamine D2 receptor), D3R (dopamine D3 receptor) and D4R (dopamine D4 receptor) subtypes [12,13,14,15]. Discovery of selective and potent ligands for the dopamine D1R and D5R subtypes has been particularly challenging due to the high degree of structural homology between these two receptor types.

Disturbances in dopaminergic neurotransmission have significant implications for neurological disorders such as Parkinson’s disease, schizophrenia and psychostimulant disorders. There is a current interest in the deployment of selective D1R full agonists/partial agonists to treat these conditions [16,17,18,19,20,21]. Classical D1R agonists contain a catechol motif that is associated with suboptimal pharmacokinetic properties, including diminished blood-brain barrier penetration and poor oral bioavailability. Only recently have selective non-catechol D1R agonists being discovered which may overcome these issues; the preclinical and clinical evaluation of non-catechol D1R agonists remains an active area of research. Selective D1R antagonists are also of recent interest in the treatment of Tourette’s syndrome and restless leg syndrome.

**Figure 1:**
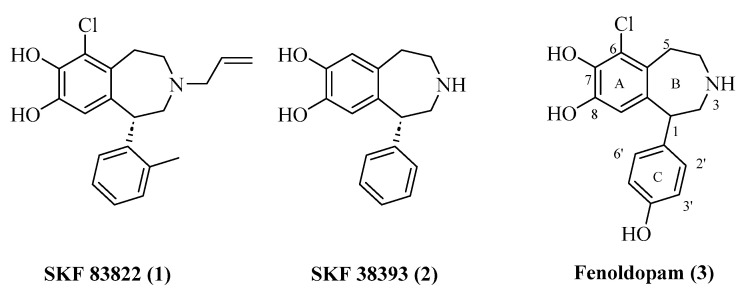
Structures of typical 1-phenylbenzazepine D1R-like ligands—SKF 83822 (**1**), SKF 38393 (**2**) and Fenoldopam (**3**).

The 1-phenylbenzazepine skeleton is a privileged D1R-like-targeted scaffold and has been extensively studied for the development of D1R-like selective ligands [22,23,24,25,26,27,28,29]. A number of these compounds have been employed as pharmacological tools. For example, SKF 83822 [30] (**1**) (with a K_i_ value of 0.2 at D1R and 0.3 nM at D5R) and SKF 38393 [31] (**2**) (with a K_i_ value of 1.0 at D1R and 0.5 nM at D5R) are two widely employed 1-phenylbenzazepines used as pharmacological tools to study D1R-like receptors (Figure 1). However, these compounds demonstrate limited selectivity between D1R and D5R, making their use limited for distinguishing the relative roles of D1R and D5R in pharmacological studies. In addition to the noted selectivity issue, D1R full and partial agonists from the 1-phenylbenzazepine class contain a catechol motif that is associated with poor pharmacokinetic properties, precluding their use as clinically useful therapeutics. The only clinically used D1R-like agonist from this class is Fenoldopam (**3**), [32] which is primarily employed as a peripherally restricted anti-hypertensive drug. 

**Figure 2:**
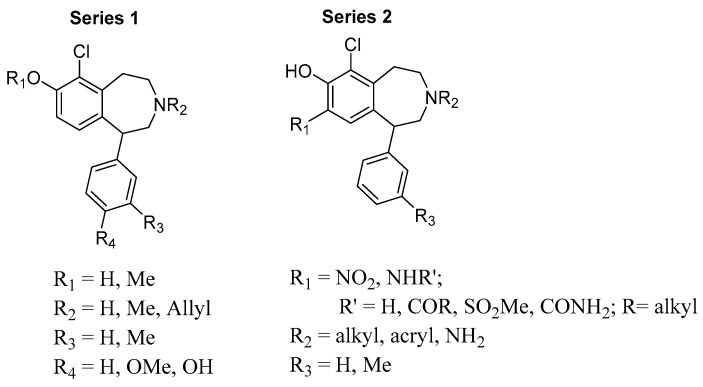
Analog library for 6-Chloro-1-phenylbenzazepine.

Previous structure–activity relationship (SAR) studies indicate that the presence of a 6-chloro group enhances D1R affinity in the scaffold, and that the 6-position is tolerant of a chloro group for D1R agonist activity. In addition, it has been established that the catechol motif is important for the retention of D1R agonist activity. The presence of a C-3′-methyl group is also known to enhance D1R affinity. The benzazepine nitrogen tolerates methyl as well as allyl substituent groups for high D1R affinity. Despite the existence of a broad body of SAR studies, there are still considerable SAR gaps that need to be addressed in order to more fully exploit the D1R targeting and drug-like capabilities of the 1-phenylbenzazepine template. In this study, we aimed to investigate the extent to which the presence of a 6-chloro group in tandem with other substitutions in the scaffold would impact D1R affinity and selectivity. Two series of 6-chloro-1-phenylbenzazepine analogs were designed (Figure 2). Series 1 analogs contain a 6-chloro-7-hydroxy or 6-chloro-7-methoxy motif and *N*-methyl, *N*-allyl or *N*-H groups in tandem with various substitutions at the C-3′ (-Me or -H) and C-4′ (-H, -OH or -OMe) positions. Series 2 compounds are broadly similar to the series 1 analogs, but are distinguished structurally by possessing a variety of nitrogen-containing substituents at C-8 (e.g., nitro, amino, amide, sulfonamide, etc.). We anticipated that compounds in series 1 would retain high D1R affinity due to the simultaneous presence of structural features associated with strong D1R affinity, e.g., the 6-chloro and 3′-methyl substituents. Similarly, it was our expectation that compounds in series 2 would also display strong D1R affinity due to the presence of similar structural features as in series 1. Furthermore, we envisaged that evaluation of compounds in series 2 that contain H-bond donor motifs at C-8 would allow us to illuminate the potential for such motifs to function as isosteric replacements of the C-8 phenol group found in classical D1R-targeted 1-phenylbenzazepine ligands, such as **1**–**3**. This, we envisaged, may open up avenues for further structural derivations towards selective D1R ligands with enhanced pharmacokinetic properties. The results of our investigation are summarized henceforth.

## 2. Results and Discussion

**Scheme 1:**
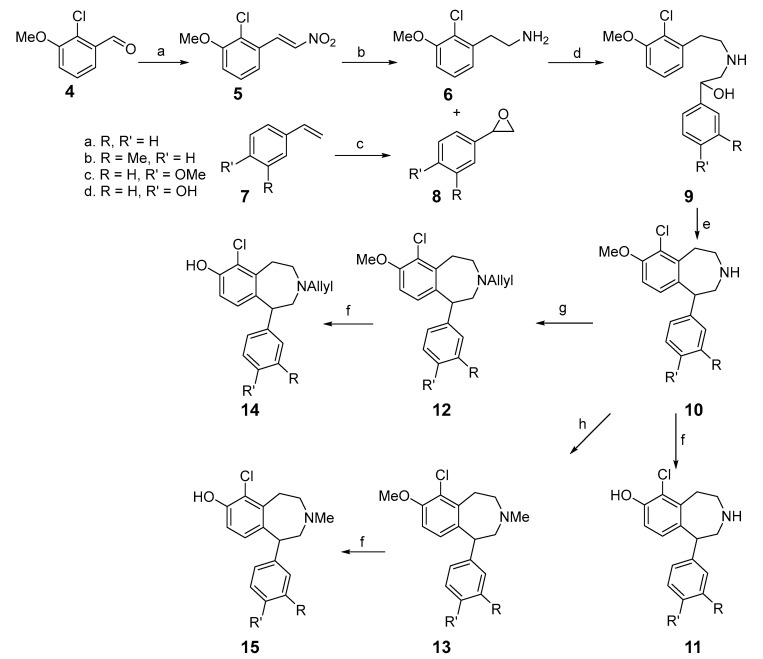
Reagents and conditions: (a) CH_3_NO_2_, NH_4_OAc, AcOH, reflux, 6 h, 92%; (b) 1. LiBH_4_, TMSCl, anhyd. THF, reflux, 6 h; 2. MeOH, 20% KOH, 45% over 2 steps; (c) 1. *m*CPBA, DCM, rt, 12 h; 2. NaOH, rt, 88–96%; (d) LiNTf_2_, THF, reflux, 24 h, 53–78%; (e) TFA, H_2_SO_4_ (18M), rt, 5 h, 36–58%; (f) BBr_3_, DCM, 0 °C, 18 h, 34–58%; (g) allyl bromide, Et_3_N, DCM, rt, 18 h, 55–63%; (h) HCHO, Na(OAc)_3_BH, ACN, rt, 18 h, 77–91%.

Synthesis of the series 1 analogs is depicted in Scheme 1. The aldehyde **4** was converted to nitrostyrene **5** via Henry reaction using nitromethane and ammonium acetate in acetic acid. Nitrostyrene **5** was then reduced with lithium borohydride to produce phenethylamine **6**. Epoxidation of phenylstyrene **7** was achieved with *m*CPBA, affording epoxide **8a**–**8d**. The amine **6** was then coupled with epoxide **8** to produce aminoalcohols **9a**–**9d**. Thereafter, aminoalcohols **9a**–**9d** were cyclized in the presence of trifluoroacetic acid and concentrated sulfuric acid to deliver benzazepines **10a**–**10d**. *O*-demethylation of benzazepines **10a**–**10d** with BBr_3_ produced phenolic benzazepines **11a**–**11d**. The free amino groups of benzazepines **10a**–**10d** were converted into the *N*-methyl derivatives **13a**–**13d** via reductive amination. The *N*-allyl analogs **12a**–**12** were obtained from compound **10a**–**10d** via S_N_2 displacement with allyl bromide. The respective *N*-substituted analogs (**12** and **13**) were then *O*-demethylated using BBr_3_ to afford the corresponding phenolic compounds **14a**–**14d** and **15a**–**15d**, respectively. 

**Scheme 2:**
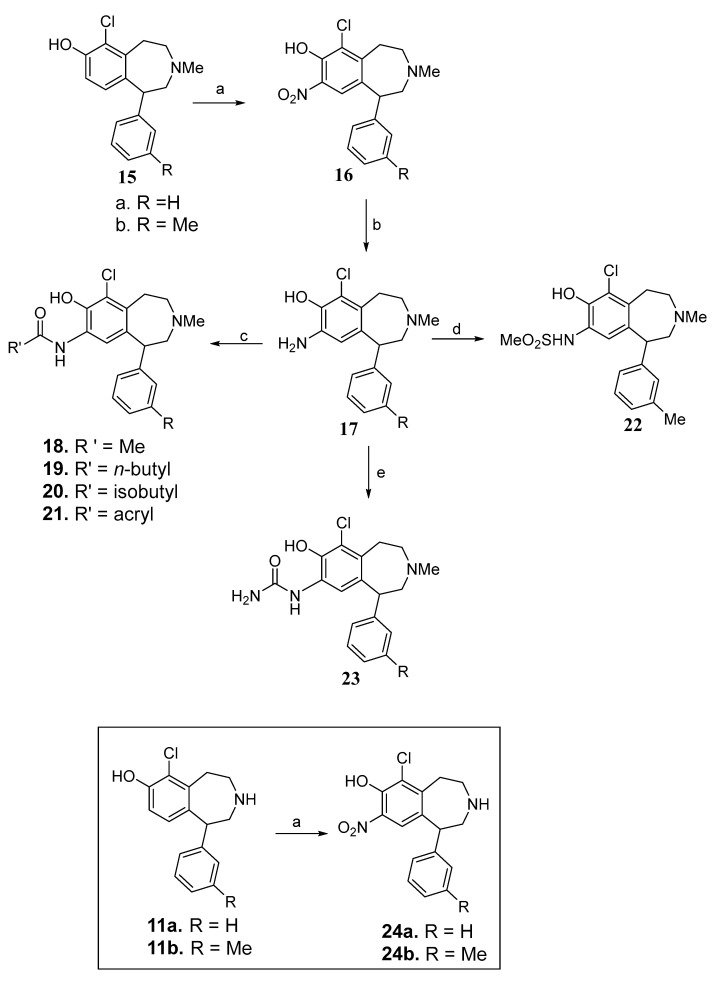
Reagents and conditions: (a) conc. HNO_3_, AcOH, rt, 2 h, 70–86%; (b) 1. LiBH_4_, TMSCl, anhyd. THF, reflux, 6 h; 2. MeOH, 20% KOH, 45–54% over 2 steps; (c) (R’CO)_2_O, DCM, rt, 3 h, 76–82%; (d) methanesulfonic anhydride, DCM, rt, 3 h, 40%; (e) KNCO, H_2_O, rt, 12 h, 76%.

Synthesis of the series 2 analogs is shown in Scheme 2. Phenols **15a**–**15b** were nitrated at the C-8 position to afford nitro compounds **16a**–**16b**. The nitro functionality of compounds **16a**–**16b** was reduced with LiBH_4_ and TMSCl to afford corresponding *o*-aminophenols **17a**–**17b**. The aminophenols **17a**–**17b** were then converted to amide derivatives **18**–**21** by treating with suitable acid anhydrides. The sulfonamide **22** was obtained by reacting aminophenol **17b** with methanesulfonic anhydride. Treatment of aminophenols **17a**–**17b** with potassium cyanate delivered the corresponding urea derivatives **23a**–**23b**. The C-8 nitro analogs **24a** and **24b** were prepared by nitration of the corresponding phenols **11a** and **11b**, respectively.

The binding affinities of analogs in series 1 (compounds **10**–**15**) and series 2 (compounds **16**–**24**) were assessed at dopamine D1R, D2R and D5R. The binding affinities of the series 1 analogs are recorded in Table 1. All compounds in this series lacked affinity for D2R, in line with our expectations based on previous SAR reports on the scaffold. Most of the compounds also lacked affinity for D1R and D5R. Compounds **14a**, **15a**, **14b**, **15b** and **15d** were the only compounds that retained D1R affinity. These compounds all contain a C-7 phenolic group; all compounds with a C-7 methoxy functionality lacked D1R affinity. Thus, it appears that the C-7 phenol group is important for retention of D1R affinity in this series. We have observed an H-bond interaction between the C-8 phenol and Asp-198 in other 1-phenylbenzazepines [28]. Others have noted H-bonding interactions between the catechol group of the 1-phenylbenzazepine SKF 81297 and Ser-198 [33]. Similar interactions may ensue with the C-7 phenol of the compounds herein; the fact that compounds with a C-7 methoxy function lack D1R affinity suggests that the C-7 phenol group may be functioning in an H-bond-donor role. Interestingly, although the C-7 phenol is important, it is not sufficient for D1R affinity, since other compounds that possess this functionality such as **11a**, **11b**, **11d** and **14d** did not bind to D1R. It appears that substituents on both the benzazepine nitrogen atom as well as the 1-phenyl moiety can also impact affinity. In particular, the data suggests that oxygenated substituents at the C-4′ position in the 1-phenyl moiety tend to diminish D1R affinity. For example, a comparison of D1R affinities of compounds in the **14a**/**14d** pair (K_i_ = 102 nM and no affinity, respectively) as well as the **15a**/**15d** pair (K_i_ = 30 and 161 nM, respectively) reinforces this conclusion. The presence of the C-3′methyl group also appears to reduce D1R affinity, as indicated from comparison of **14a**/**14b** and **15a**/**15b** where the C-3′ methylated analogs **14b** and **15b** showed lower D1R affinities than their analogous demethylated counterparts **14a** and **15a**, respectively. With regards to the effect of the *N*-substituent on D1R affinity, it was observed that compounds with an *N*-methyl substituent showed higher D1R affinity than analogous compounds with an *N*-allyl moiety and that compounds with an *N*-H moiety showed lower affinity than compounds with an *N*-allyl moiety. Thus, the overall trend in increasing D1R affinity is *N*-H < *N*-allyl < *N*-methyl. Evidence for this trend comes from examination of the data for the analogous *N*-H, *N*-allyl and *N*-methyl compounds **11a**, **14a** and **15a** (K_i_ = no affinity, 102 nM and 30 nM, respectively). A similar trend was also observed for the **11b**, **14b** and **15b** subset (K_i_ = no affinity, 281 nM and 78 nM, respectively). 

Compounds that displayed D1R affinity (**14a**, **14b**, **15a**, **15b** and **15d**) also had affinity for D5R. Furthermore, the compounds were generally moderately selective for D1R over D5R (ranging from roughly 2- to 6-fold). Compound **11a** is anomalous in this regard, as it showed moderate D5R affinity (K_i_ = 470 nM) and no D1R affinity. Similar to what was observed at the D1R, we also noted that the *N*-substituent affected the D5R affinity according to the trend *N*-H < *N*-allyl < *N*-methyl (e.g., compare **11a**, **14a** and **15a**; K_i_ = 470 nM, 297 nM and 187 nM, respectively). Other general trends at D1R were also seen at D5R (e.g., preference for C-7 phenol versus C-7 methoxy; C-3′ methyl diminishes D5R affinity).

Affinity data for the series 2 compounds are collated in Table 2. As was seen for the series 1 compounds, the analogs in this series showed no affinity for D2R. Additionally, they generally lacked affinity for D5R, had low to moderate D1R affinity and displayed moderate selectivity for D1R over D5R. Analysis of the data indicates that the presence of a C-8 nitro group significantly reduces D1R affinity as compared to the series 1 group (i.e., compare compounds **15a** and **15b** from series 1 with the analogous C-8 nitro analogs **16a** and **16b** from series 2). Replacement of the C-8 nitro group with a C-8 amino functionality restores D1R affinity, as can be seen from comparison of the **16a**/**17a** and **16b**/**17b** pairs; K_i_ = no affinity/210 nM and 4354nM/72 nM, respectively. This improvement in D1R affinity may be attributable to the H-bond-donor characteristics of the amino functionality and suggests that the C-8 amino group is functioning in a similar role as the C-8 phenol group found in classical 1-phenylbenzazepine ligands such as **1**–**3**. For the amide analogs (**18a**–**21b**), the D1R affinities were generally low (ranging from no affinity to 634 nM) and there was no clear SAR trend with regards to size or electronics of the amide unit or the C-3′ substituent. Overall, amide groups at C-8 are tolerated less than a C-8 amino functionality for D1R affinity. However, the methanesulfonamide analog **22** showed significantly higher affinity than the amide analogs and was in a comparable affinity range (K_i_ = 113 nM) to the C-8 amino analogs. Like the amide analogs, the urea derivatives **23a** and **23b** had low D1R affinities. For the C-8 nitro analogs that had no alkyl substituent on the benzazepine nitrogen (**24a** and **24b**), it was interesting to observe that the presence of a C-3′ methyl group in **24b** versus its absence led to a significant improvement in D1R affinity (K_i_ = no affinity and 38 nM for **24a** and **24b**, respectively). Furthermore, comparison of **16b** and **24b** suggests that the *N*-methyl substituent group in **16b** is not necessary for D1R affinity. This effect of the *N*-substituent contrasts somewhat with the trend that we observed for the series 1 analogs. If one assumes that the compounds in the **16** and **24** subsets bind to D1R in a similar manner, it may be inferred that the C-3′ methyl group present in **16b** and **24b** makes a significant contribution to the affinity of the compounds. 

The compound with the highest affinity and D1R selectivity from our studies (compound **15a**) was further evaluated for D1R functional activity in agonist and antagonist mode with cAMP-based functional assays. Dopamine and SCH 39166 served as the controls for D1R agonistic and antagonistic assays (Table 3). Compound **15a** displayed antagonist activity in these assays with an IC_50_ value of 130.32 nM. No D1R agonistic activity was observed for this compound.

## 3. Conclusions

In summary, our evaluation focused on investigating the effect of C-7, C-8, *N*-3, C-3′ and C-4′ substitutions on modified 6-chloro-1-phenylbenzazepines to expand the SAR footprint of 1-phenylbenzazepines as D1R ligands. Our studies reinforce that the catechol motif present in classical 1-phenylbenzazepines D1R ligands is a critical motif for D1R affinity, as modifications to this motif generally reduced D1R affinity via both series 1 and series 2. That being said, the C-8 position appears to be tolerant of amino and methanesulfonamide groups towards D1R affinity, possibly serving in an isosteric H-bond-donor capacity to mimic a C-8 phenol group. Furthermore, the compounds with the highest D1R affinities identified herein either lack a C-8 substituent (compound **15a**) or possess a C-8 nitro group (compound **24b**), affirming that a catechol motif, while important, is not absolutely required for D1R affinity in the scaffold. Our SAR data further suggests that substituents on the benzazepine nitrogen atom, as well as substituents in the 1-phenyl ring, can contribute significantly to D1R affinity of the 6-chloro-1-phenylbenzazepines. Compounds with D1R affinity displayed modest selectivity versus D5R and were generally devoid of D2R affinity, indicating that a similar selectivity profile may be maintained as compared to the classical, catecholic 1-phenylbenzazepine D1R ligands. Compound **15a** was revealed as a new, moderately selective D1R antagonist and has favorable predicted drug-like characteristics based on Lipinski rules (clogP = 4.0; H-bond donors = 1; H-bond acceptors = 2; molecular weight = 287). Further physicochemical and pharmacokinetic evaluations of the compound are merited and will be the subject of future investigations.

## 4. Materials and Methods

### 4.1. General Synthetic Experimental Procedures

Unless otherwise stated, chemicals were purchased from Fisher Scientific (Boston, MA, USA) and used without further purification. Reactions were carried out in oven-dried glassware under a nitrogen atmosphere. HRESIMS spectra were obtained using an Agilent 6520 QTOF instrument. ^1^H NMR and ^13^C NMR spectra were recorded using Bruker DPX-600 spectrometer (operating at 600 MHz for ^1^H; 150 MHz, for C), Bruker NEO-500 spectrometer (operating at 500 MHz for ^1^H; 125 MHz, for ^13^C) or Bruker DPX-400 spectrometer (operating at 400 MHz for ^1^H; 100 MHz, for ^13^C) using CDCl_3_ as solvent, unless stated otherwise. Tetramethylsilane (δ 0.00 ppm) served as an internal standard in 1H NMR and CDCl3 (δ 77.0 ppm) in 13C NMR as solvent. Chemical shift (δ 0.00 ppm) values are reported in parts per million and coupling constants in Hertz (Hz). Splitting patterns are described as singlet (s), doublet (d), triplet (t) and multiplet (m). Reactions were monitored by TLC with Whatman Flexible TLC silica gel G/UV 254 precoated plates (0.25 mm). TLC plates were visualized by UV (254 nm). Silica gel column chromatography was performed with silica gel 60 (EMD Chemicals, 230–400 mesh, 0.063 mm particle size).

### 4.2. General Procedure A: Epoxidation of Styrenes

To a stirred solution of desired styrene **7** (1 equiv.) in DCM (125 mL), mCPBA (2 equiv.) was slowly added at 0 °C and the resulting reaction mixture was warmed to room temperature and stirred at the same temperature for 12 h. The progress of the reaction was monitored by TLC. After completion of the reaction, the reaction mixture was quenched with aqueous NaOH (3M, 100 mL) and extracted with DCM (3 × 50 mL). The combined organic layer was washed with brine, dried over anhydrous Na_2_SO_4_ and concentrated under reduced pressure to provide the product **8**, which was used in the subsequent step without further purification.

### 4.3. General Procedure B: Coupling of 2-(4-(Benzyloxy)-3-methoxyphenyl)Ethan-1-amine with Epoxides

Bis(trifluoromethane)sulfonimide lithium (1.1 equiv.) was slowly added to a solution of epoxide **8** (1 equiv.) and amine **6** (1.5 equiv.) in anhydrous THF (100 mL) at rt and the resulting reaction mixture was refluxed for 24 h. After completion (the reaction progress was monitored by TLC), THF was evaporated and the reaction mixture was quenched with saturated NaHCO_3_ (25 mL) and extracted with EtOAc (3 × 30 mL). The combined organic phase was dried with Na_2_SO_4_ and concentrated to provide crude amino alcohol **9** as a viscous oil which was used in the next step without further purification.

### 4.4. General Procedure C: Amino Alcohol Cyclization

To a stirred solution of respective amino alcohols **9** (0.02 mol, 1 equiv.) in TFA (0.4 mol, 20 mL) was 18 M H_2_SO_4_ (0.01 mol, 0.5 mL) added at rt, and the resulting reaction mixture was stirred for 5 h at rt. Anhydrous NaOAc (2 equiv.) was added and stirred for an additional 15 min. TFA was evaporated and the reaction mixture was neutralized with 14 M NH_4_OH to produce a solid residue. The residue was dissolved in EtOAc (25 mL), washed with water and brine, and the organic phase was dried over Na_2_SO_4_ and concentrated under reduced pressure to produce a crude residue, which was purified by silica gel column chromatography to produce a pure product of **10**.

### 4.5. General Procedure D: N-Methylation

To a stirred solution of secondary amine **10** (1.4 mmol, 1 equiv.) in acetonitrile (100 mL) was paraformaldehyde (4.2 mmol, 3 equiv.) added and the reaction mixture was stirred at room temperature for 30 min. Sodium triacetoxyborohydride (2.1 mmol, 1.5 equiv.) was added to the reaction mixture and the resulting reaction mixture was stirred for 12 h at the same temperature. After completion of the reaction (monitored by TLC), it was quenched with saturated NaHCO_3_ (20 mL), washed with water, extracted with EtOAc (2 × 25 mL), dried over anhydrous Na_2_SO_4_ and evaporated under reduced pressure to produce a crude compound, which was purified by silica gel flash chromatography to afford pure *N*-methylated product **13**.

### 4.6. General Procedure E: N-Allylation

To a stirred solution of secondary amine **10** (2.01 mmol, 1 equiv.) in acetonitrile (80 mL), TEA (3.98 mmol, 2 equiv.) was added followed by allyl bromide (2.95 mmol, 1.5 equiv.) at room temperature, and the resulting reaction mixture was stirred at the same temperature for 12 h. After completion of the reaction (monitored by TLC), the reaction mixture was quenched with water, washed with NaHCO_3_ (25 mL) and extracted with EtOAc (2 × 25 mL). The combined organic layer was dried over anhydrous Na_2_SO_4_ and evaporated under vacuum to produce a crude compound, which was purified by silica gel flash chromatography to produce pure *N*-allylated product **12**.

### 4.7. General Procedure F: O-Demethylation with BBr_3_

To a stirred solution of compound **10**/**12**/**13** (0.29 mmol, 1 equiv.) in DCM (20 mL), BBr_3_ (1.2 mmol, 4 equiv.) was slowly added at 0 °C and the resulting reaction mixture was warmed to room temperature and stirred at the same temperature for 18 h. After completion (monitored by TLC), ice water was added to the reaction mixture and stirred for 30 min, and the obtained precipitate was filtered to produce pure compounds of **11**/**14**/**15**.

### 4.8. General Procedure G: Ortho-Nitration of Phenol

Substituted phenol **15** (1 equiv.) was dissolved in acetic acid (5 mL), and fuming nitric acid (1 equiv.) was slowly added to the solution. The reaction was stirred at rt for 2 h. After completion of the reaction (confirmed by the TLC), acetic acid was evaporated under vacuum to obtain a yellow residue. The crude residue was basified with sat. NaHCO_3_ and extracted with EtOAc (2 × 25 mL). The combined organic layer was dried over anhydrous Na_2_SO_4_ and evaporated under vacuum to produce a crude compound as a yellow solid, which was purified by silica gel flash chromatography to produce a pure compound of **16**.

### 4.9. General Procedure H: Reduction of O-Nitrophenol

Lithium borohydride (4 equiv.) was suspended in anhydrous THF under a nitrogen atmosphere and the suspension was cooled to 0 °C. Trimethyl chlorosilane was added to the mixture and stirred for 15 min. The 2-nitrophenol **16** was added slowly to the reaction mixture in portions and the reaction mixture was refluxed overnight. After completion of the reaction as confirmed by TLC, it was cooled to 0 °C and quenched with methanol. The clear solution obtained was evaporated under vacuum to obtain a viscous residue. The residue was basified with sat. NaHCO_3_ to obtain a white solid, which was purified using silica gel flash chromatography to produce the pure compound of **17**.

### 4.10. General Procedure I: Amidation of O-Aminophenol

To a stirred solution of *o*-aminophenol, **17** (1 equiv.) in DCM (20 mL) was added the corresponding anhydrides (1.5 equiv.). The reaction was stirred for 3 h. After completion of the reaction (confirmed by TLC), the mixture was diluted with hexane to obtain a cloudy solid that was filtered to produce an off-white solid. The final product was purified using silica gel flash chromatography to obtain the pure product (**18**/**19**/**20**/**21**/**22**) as a white solid.

*6-chloro-7-methoxy-1-phenyl-2,3,4,5-tetrahydro-1H-benzo[d]azepine* (**10a**): Synthesized according to general procedure C. White solid. Yield: 46%. mp: 156.4–158.0 °C.

^1^H NMR (600 MHz, CDCl_3_) δ 7.38 (t, *J* = 15 Hz, 2H), 7.32 (d, *J* = 7.32 Hz, 1H), 7.12 (d, *J* = 8.50 Hz, 2H) 6.64 (d, *J* = 8.64 Hz, 1H) 6.44 (d, *J* = 8.70 Hz, 1H) 4.67 (d, *J* = 9.66 Hz, 1H) 3.84 (s, 3H), 3.70 (m, 2H), 3.50 (m, 1H), 3.7 (m, 2H), 2.94–2.90 (m, 1 Hz, 1H) ppm. ^13^C NMR (150 MHz, CDCl_3_) δ 154.1, 139.8, 137.2, 135.7, 129.2, 128.2, 127.6, 127.2, 122.7, 109.8, 77.2, 76.8, 56.2, 50.6, 45.6, 44.7, 27.4 ppm. HRESIMS calculated for C_17_H_19_ClNO [M + H]^+^ 288.1077, found 288.1076.

*6-chloro-7-methoxy-1-(m-tolyl)-2,3,4,5-tetrahydro-1H-benzo[d]azepine* (**10b**): Synthesized according to general procedure C. White solid. Yield: 46%. mp: 180.4–181.8 °C.

^1^H NMR (400 MHz, CDCl_3_) δ 7.28 (s, 1H), 7.26 (d, *J* = 7.6 Hz, 1H), 7.13 (d, *J* = 7.5 Hz, 1H), 6.94 (s, 1H), 6.91 (d, *J* = 7.8 Hz, 1H), 6.67 (d, *J* = 8.7 Hz, 1H), 6.53 (d, *J* = 8.7 Hz, 1H), 4.61 (d, *J* = 9.3 Hz, 1H), 3.86 (s, 3H), 3.74 (d, *J* = 12.9 Hz, 1H), 3.66 (dd, *J* = 16.1, 7.0 Hz, 1H), 3.51 (dd, *J* = 13.0, 7.2 Hz, 1H), 3.45–3.36 (m, 2H), 3.00–2.92 (m, 1H), 2.35 (s, 3H) ppm. ^13^C NMR (100 MHz, CDCl_3_) δ 154.5, 139.3, 136.3, 134.2, 129.3, 128.6, 128.4, 128.2, 124.7, 120.7, 118.2, 110.3, 56.3, 50.8, 45.9, 45.8, 27.0, 21.4 ppm. HRESIMS calculated for C_18_H_21_ClNO [M + H]^+^ 302.1233, found 302.1300.

*6-chloro-7-methoxy-1-(4-methoxyphenyl)-2,3,4,5-tetrahydro-1H-benzo[d]azepine* (**10c**): Synthesized according to general procedure C. Yield: 47%. mp: 151.3–152.7 °C.

^1^H NMR (400 MHz, DMSO-d6) δ 7.15 (d, *J* = 8.6 Hz, 2H), 7.00 (d, *J* = 8.6 Hz, 2H), 6.92 (d, *J* = 8.7 Hz, 1H), 6.53 (d, *J* = 8.7 Hz, 1H), 4.61 (t, *J* = 5.5 Hz, 1H), 3.81 (s, 1H), 3.80 (s, 3H), 3.78 (s, 3H), 3.76 (s, 1H), 3.72 (dd, *J* = 8.1, 3.7 Hz, 2H), 3.56 (d, *J* = 5.5 Hz, 1H), 3.01 (t, *J* = 10.3 Hz, 1H) ppm. ^13^C NMR (100 MHz, DMSO-d6) δ 158.7, 153.9, 137.6, 136.3, 132.5, 129.7, 128.0, 124.7, 121.5, 118.3, 114.8, 110.8, 56.6, 55.5, 49.6, 44.72, 44.29, 27.2 ppm. HRESIMS calculated for C_18_H_21_ClNO_2_ [M + H]^+^ 318.1183, found 318.1170.

*6-chloro-1-phenyl-2,3,4,5-tetrahydro-1H-benzo[d]azepin-7-ol* (**11a**): Synthesized according to general procedure F. Off-white solid. Yield: 43%. mp: 134.6–135.8 °C.

^1^H NMR (600 MHz, CD_3_OD) δ 7.42 (t, *J* = 15.2 Hz, 2H), 7.34 (t, *J* = 12.3 Hz, 1H), 7.21 (d, *J* = 7.4 HZ, 2H) 6.71 (d, *J* = 8.4 Hz, 1H) 6.54 (d, *J* = 8.8 Hz, 1H) 4.67 (d, *J* = 2.1 Hz, 1H) 3.73 (m, 2H), 3.53 (m, 3H), 3.16 (t, *J* = 10.7, 3H) ppm. ^13^C NMR (150 MHz, CD_3_OD) δ 162.7, 162.4, 154.1, 139.0, 137.3, 135.73, 129.2, 128.2, 127.6, 127.2, 122.7, 109.8, 56.2, 50.6, 45.6, 44.7, 27.4 ppm. HRESIMS calculated for C_16_H_17_ClNO [M + H]^+^ 274.0920, found 274.0922.

*6-chloro-1-(m-tolyl)-2,3,4,5-tetrahydro-1H-benzo[d]azepin-7-ol* (**11b**): Synthesized according to *O*-demethylation procedure F. Gray solid. Yield: 31%. mp: 122.3–124.2 °C.

^1^H NMR (600 MHz, DMSO-d6) δ 7.31 (t, *J* = 7.6 Hz, 1H), 7.15 (d, *J* = 7.6 Hz, 1H), 7.05 (s, 1H), 6.99 (d, *J* = 7.5 Hz, 1H), 6.75 (d, *J* = 8.5 Hz, 1H), 6.38 (d, *J* = 8.5 Hz, 1H), 4.61–4.56 (m, 1H), 3.54 (d, *J* = 4.9 Hz, 2H), 3.47–3.41 (m, 2H), 3.36–3.30 (m, 1H), 2.99 (t, *J* = 10.7 Hz, 1H), 2.33 (s, 3H) ppm. ^13^C NMR (150 MHz, DMSO-d6) δ 151.7, 140.2, 137.7, 136.9, 133.8, 128.5, 128.4, 127.4, 127.0, 124.9, 119.9, 113.6, 48.8, 44.8, 43.6, 26.7, 20.8 ppm. HRESIMS calculated for C_17_H_19_ClNO [M + H]^+^ 288.1077, found 288.1100.

*6-chloro-1-(4-hydroxyphenyl)-2,3,4,5-tetrahydro-1H-benzo[d]azepin-7-ol* (**11c**): Synthesized according to general procedure F. Gray solid. Yield: 38%. mp: 135.8–136.9 °C.

^1^H NMR (400 MHz, DMSO-d6) δ 6.96 (d, *J* = 8.1 Hz, 2H), 6.76 (d, *J* = 8.0 Hz, 2H), 6.69 (d, *J* = 8.3 Hz, 2H), 6.43 (d, *J* = 8.4 Hz, 1H), 5.76 (s, 1H), 4.31 (d, *J* = 5.0 Hz, 1H), 3.25–3.11 (m, 1H), 3.09–2.90 (m, 2H), 2.88–2.79 (m, 2H), 2.68 (s, 1H) ppm. ^13^C NMR (100 MHz, CDCl_3_) δ 155.6, 136.3, 129.7, 129.2, 129.1, 128.9, 126.7, 126.3, 126.2, 116.1, 75.0, 59.0, 56.3, 28.9 ppm.

HRESIMS calculated for C_16_H_17_ClNO_2_ [M + H]^+^ 290.0870, found 290.0879.

*3-allyl-6-chloro-7-methoxy-1-(m-tolyl)-2,3,4,5-tetrahydro-1H-benzo[d]azepine* (**12b**): Synthesized according to general procedure E. Yield: 58%. mp: 172.1–173.8 °C.

^1^H NMR (400 MHz, CDCl_3_) δ 7.28 (s, 1H), 7.26 (d, *J* = 7.5 Hz, 1H), 7.10 (d, *J* = 7.5 Hz, 1H), 7.00 (s, 1H), 6.64 (d, *J* = 8.6 Hz, 1H), 6.55 (d, *J* = 8.6 Hz, 1H), 5.97–5.87 (m, 1H), 5.20 (t, *J* = 12.3 Hz, 2H), 4.34 (d, *J* = 8.4 Hz, 1H), 3.87 (s, 3H), 3.51–3.46 (m, 1H), 3.25–3.11 (m, 4H), 3.03 (dd, *J* = 10.1, 9.7 Hz, 1H), 2.92 (dd, *J* = 11.8, 9.0 Hz, 1H), 2.47–2.39 (m, 1H), 2.37 (s, 3H) ppm. ^13^C NMR (100 MHz, CDCl_3_) δ 153.4, 143.0, 140.1, 138.3, 138.1, 134.9, 129.1, 128.4, 127.2, 126.7, 125.3, 122.1, 118.37, 108.8, 62.4, 60.3, 56.1, 54.0, 49.3, 30.5, 21.5 ppm. HRESIMS calculated for C_21_H_25_ClNO [M + H]^+^ 342.1546, found 342.1600.

*3-allyl-6-chloro-7-methoxy-1-(4-methoxyphenyl)-2,3,4,5-tetrahydro-1H-benzo[d]azepine* (**12c**): Synthesized according to general procedure E. Yield: 61%. mp: 155.4–157.1 °C.

^1^H NMR (400 MHz, CDCl_3_) δ 7.07 (d, *J* = 8.7 Hz, 2H), 6.97 (d, *J* = 8.7 Hz, 2H), 6.70 (d, *J* = 8.7 Hz, 1H), 6.58 (d, *J* = 8.2 Hz, 1H), 5.95 (m, 1H), 5.60–5.51 (m, 2H), 4.60 (d, *J* = 9.6 Hz, 1H), 3.93 (dd, *J* = 9.9, 3.5 Hz, 1H), 3.87 (s, 3H), 3.86 (s, 3H), 3.85–3.82 (m, 2H), 3.81 (d, *J* = 3.8 Hz, 1H), 3.72 (d, *J* = 6.3 Hz, 2H), 3.40 (dd, *J* = 16.4, 10.7 Hz, 2H) ppm. ^13^C NMR (125 MHz, Acetone-d6) δ 159.9, 155.2, 137.7, 130.1, 128.9, 126.4, 125.7, 124.8, 122.93, 122.33, 119.7, 115.42, 115.34, 111.2, 61.9, 56.6, 55.6, 54.0, 30.4, 29.3, 27.8 ppm. HRESIMS calculated for C_21_H_25_ClNO_2_ [M + H]^+^ 358.1496, found 358.1496.

*6-chloro-7-methoxy-3-methyl-1-(m-tolyl)-2,3,4,5-tetrahydro-1H-benzo[d]azepine* (**13b**): Synthesized according to general procedure D. Yield: 81%. mp: 174.5–176.1 °C.

^1^H NMR (500 MHz, Acetone-d6) δ 7.31 (t, *J* = 7.6 Hz, 2H), 7.18 (d, *J* = 7.6 Hz, 1H), 7.09 (s, 1H), 7.04 (d, *J* = 7.7 Hz, 1H), 6.91 (d, *J* = 8.6 Hz, 1H), 4.77 (d, *J* = 10.1 Hz, 1H), 3.88 (s, 3H), 3.81–3.72 (m, 2H), 3.70–3.61 (m, 1H), 3.55–3.50 (m, 1H), 3.28–3.22 (m, 1H), 3.09 (s, 3H), 2.33 (s, 3H), 2.31–2.26 (m, 1H) ppm. ^13^C NMR (125 MHz, Acetone-d6) δ 155.1, 141.3, 139.4, 138.1, 129.8, 128.8, 128.3, 126.2, 122.8, 122.3, 119.7, 111.0, 61.5, 56.6, 55.9, 46.6, 46.3, 28.2, 21.5 ppm. HRESIMS calculated for C_19_H_23_ClNO [M + H]^+^ 316.1390, found 316.1390.

*6-chloro-7-methoxy-1-(4-methoxyphenyl)-3-methyl-2,3,4,5-tetrahydro-1H-benzo[d]azepine* (**13c**): Synthesized according to general procedure D. Yield: 82%. mp: 155.4–157.1 °C.

^1^H NMR (500 MHz, Acetone-d6) δ 7.16 (d, *J* = 8.6 Hz, 2H), 6.94 (d, *J* = 8.7 Hz, 2H), 6.84 (d, *J* = 8.6 Hz, 1H), 6.66 (d, *J* = 7.7 Hz, 1H), 4.44–4.39 (m, 1H), 3.90–3.87 (m, 1H), 3.85 (s, 3H), 3.81 (s, 3H), 3.76 (d, *J* = 2.3 Hz, 1H), 3.24 (dd, *J* = 14.6, 9.5 Hz, 1H), 3.10–3.14 (m, 1H), 3.00–2.89 (m, 1H), 2.64–2.55 (m, 1H), 2.51 (s, 3H). ^13^C NMR (125 MHz, Acetone-d6) δ 211.8, 206.6, 159.2, 154.6, 130.1, 124.8, 122.5, 119.7, 117.1, 110.1, 62.8, 56.7, 56.4, 55.5, 48.6, 47.4 ppm. HRESIMS calculated for C_19_H_23_ClNO_2_ [M + H]^+^ 333.1339, found 333.1320.

*6-chloro-3-methyl-1-phenyl-2,3,4,5-tetrahydro-1H-benzo[d]azepin-7-ol* (**15a**): Synthesized according to general procedure F. Gray solid. Yield: 28%. mp: 143.5–145.1 °C.

^1^H NMR (600 MHz, CDCl_3_) δ 7.34 (t, *J* = 14.8 Hz, 2H), 7.26 (t, *J* = 10.4 Hz, 1H), 7.16 (d, *J* = 7.6 Hz, 2H), 6.69 (d, *J* = 8.7 Hz, 1H), 6.46 (d, *J* = 8.2 Hz, 1H), 4.33 (d, *J* = 8.4 Hz, 1H), 3.34 (m, 2H), 3.18 (m, 2H), 3.10 (m, 2H), 2.80 (s, 3H) ppm. ^13^C NMR (600 MHz, CDCl_3_) δ 149.8, 142.9, 138.9, 137.8, 128.6, 128.3, 127.3, 126.5, 120.2, 112.9, 62.6, 55.9, 49.2, 47.4, 30.8 ppm. C_17_H_19_ClNO [M + H]^+^ 288.1077, found 288.1170.

*6-chloro-3-methyl-1-(m-tolyl)-2,3,4,5-tetrahydro-1H-benzo[d]azepin-7-ol (***15b**): Synthesized according to general procedure F. Yield: 51%. mp: 142.6–144.0 °C.

^1^H NMR (600 MHz, DMSO-d6) δ 10.08 (s, 1H), 7.31 (t, *J* = 7.6 Hz, 1H), 7.16 (d, *J* = 7.4 Hz, 1H), 7.05 (s, 1H), 7.00 (d, *J* = 7.3 Hz, 1H), 6.74 (d, *J* = 8.3 Hz, 1H), 4.56 (d, *J* = 6.7 Hz, 1H), 3.59 –3.56 (m, 3H), 3.32 (s, 3H), 2.77 (s, 3H), 2.33 (s, 3H) ppm. ^13^C NMR (125 MHz, DMSO-d6) δ 151.9, 138.0, 137.1, 128.7, 127.6, 125.3, 124.1, 123.2, 120.7, 119.9, 118.1, 113.8, 59.6, 53.7, 44.5,44.4 27.0, 21.0 ppm. HRESIMS calculated for C_18_H_21_ClNO [M + H]^+^ 302.1233, found 302.1236.

*3-allyl-6-chloro-1-(m-tolyl)-2,3,4,5-tetrahydro-1H-benzo[d]azepin-7-ol* (**14b**): Synthesized according to general procedure F. Off-white solid. Yield: 48%. mp: 151.3–152.7 °C.

^1^H NMR (400 MHz, DMSO-d6) δ 10.45 (s, 1H), 7.35 (t, *J* = 7.6 Hz, 1H), 7.19 (d, *J* = 7.6 Hz, 1H), 7.07 (s, 1H), 7.04 (d, *J* = 7.6 Hz, 1H), 6.73 (d, *J* = 8.6 Hz, 1H), 6.20 (d, *J* = 8.6 Hz, 1H), 6.01 (td, *J* = 17.1, 7.0 Hz, 1H), 5.55 (s, 1H), 5.53–5.48 (m, 1H), 4.72 (d, *J* = 10.1 Hz, 1H), 3.85–3.76 (m, 3H), 3.67–3.65 (m, 1H), 3.52–3.43 (m, 3H), 3.02–2.94 (m, 1H), 2.34 (s, 3H) ppm. ^13^C NMR (100 MHz, DMSO-d6) δ 152.4, 141.2, 138.6, 137.1, 134.9, 129.4, 129.3, 128.4, 127.9, 127.7, 126.7, 125.9, 120.4, 114.4, 59.8, 58.1, 51.8, 44.3, 26.7, 21.5 ppm. HRESIMS calculated for C_20_H_23_ClNO [M + H]^+^ 328.1390, found 328.1382.

*3-allyl-6-chloro-1-(4-hydroxyphenyl)-2,3,4,5-tetrahydro-1H-benzo[d]azepin-7-ol* (**14c**): Synthesized according to general procedure F. Off-white solid. Yield: 34%. mp: 132.5–134.1 °C.

^1^H NMR (400 MHz, Acetone-d6) δ 7.10 (d, *J* = 8.7 Hz, 2H), 6.93 (d, *J* = 8.7 Hz, 2H), 6.77 (d, *J* = 8.6 Hz, 1H), 6.36 (d, *J* = 8.6 Hz, 1H), 5.58–5.55 (m, 2H), 5.25 (d, *J* = 10.0 Hz, 1H), 4.34–4.29 (m, 1H), 4.11 (dd, *J* = 15.4, 8.1 Hz, 1H), 3.95–3.70 (m, 4H), 3.57–3.40 (m, 3H), 3.09–3.05 (m, 1H) ppm. ^13^C NMR (100 MHz, Acetone-d6) δ 160.2, 155.5, 137.9, 130.4, 130.0, 125.8, 125.1, 123.1, 122.5, 120.0, 115.5, 111.5, 62.2, 56.8, 54.2, 46.3, 28.1, 26.7, 21.5 ppm. HRESIMS calculated for C_19_H_21_ClNO_2_ [M + H]^+^ 330.1183, found 330.1211.

*6-chloro-3-methyl-8-nitro-1-phenyl-2,3,4,5-tetrahydro-1H-benzo[d]azepin-7-ol* (**16a**): Synthesized according to general procedure G. Yellow solid. Yield: 80%. mp: 198.7–200.0 °C

^1^H NMR (600 MHz, Acetone-d6) δ 7.60 (s, 1H), 7.49–7.45 (m, 2H), 7.40 (dd, *J* = 13.0, 7.2 Hz, 2H), 7.34 (d, *J* = 8.0 Hz, 1H), 5.20 (d, *J* = 6.0 Hz, 1H), 4.11–3.95 (m, 2H), 3.88–3.76 (m, 1H), 3.69–3.58 (m, 1H), 3.50–3.36 (m, 2H), 3.14 (s, 3H) ppm. ^13^C NMR (150 MHz, CDCl_3_) 140.9, 136.5, 131.9, 128.1, 128.0, 127.6, 126.4, 126.1, 118.9, 113.2, 64.8, 58.6, 42.0, 25.3, 24.7 ppm. HRESIMS calculated for C_17_H_18_ClN_2_O_3_ [M + H]^+^ 333.0298, found 333.0220.

*6-chloro-3-methyl-8-nitro-1-(m-tolyl)-2,3,4,5-tetrahydro-1H-benzo[d]azepin-7-ol* (**16b**): Synthesized according to general procedure G. Yellow solid. Yield: 78%. mp: 210.4–212.6 °C.

^1^H NMR (600 MHz, Acetone-d6) δ 7.23 (t, *J* = 7.6 Hz, 1H), 7.19 (s, 1H), 7.09 (d, *J* = 7.5 Hz, 1H), 7.04 (s, 1H), 6.99 (d, *J* = 7.5 Hz, 1H), 4.88 (d, *J* = 9.4 Hz, 1H), 3.80 (dd, *J* = 13.7, 9.7 Hz, 2H), 3.71 (d, *J* = 14.7 Hz, 1H), 3.63–3.57 (m, 2H), 3.15 (s, 1H), 2.93 (s, 3H), 2.22 (s, 3H) ppm. ^13^C NMR (150 MHz, Acetone-d6) δ 150.0, 146.1, 139.6, 138.9, 132.9, 129.2, 129.1, 128.5, 125.4, 124.0, 122.0, 121.2, 60.3, 53.9, 45.0, 44.8, 28.0, 20.5 ppm. HRESIMS calculated for C_18_H_20_ClN_2_O_3_ [M + H]^+^ 347.1084, found 347.1100.

*8-amino-6-chloro-3-methyl-1-phenyl-2,3,4,5-tetrahydro-1H-benzo[d]azepin-7-ol* (**17a**): Synthesized according to general procedure H. Off-white solid. Yield: 45%. mp: 137.0–138.9 °C.

^1^H NMR (600 MHz, CDCl_3_) δ 7.32 (d, *J* = 7.8 Hz, 2H), 7.27–7.23 (m, 1H), 7.08 (dd, *J* = 14.3, 7.6 Hz, 3H), 5.78 (s, 1H), 5.72 (s, 1H), 4.25 (d, *J* = 10.4 Hz, 1H), 3.53 (dt, *J* = 14.4, 10.6 Hz, 2H), 3.26 (dd, *J* = 16.7, 4.7 Hz, 2H), 3.10–3.05 (m, 1H), 2.96 (t, *J* = 10.9 Hz, 1H), 2.57 (s, 3H) ppm. ^13^C NMR (150 MHz, CDCl_3_) δ 141.9, 137.3, 136.3, 132.9, 128.9, 128.6, 127.1, 125.4, 119.9, 114.2, 65.7, 59.6, 43.0, 26.3, 25.6 ppm. HRESIMS calculated for C_17_H_20_ClN_2_O [M + H]^+^ 303.1186, found 303.1187.

*8-amino-6-chloro-3-methyl-1-(m-tolyl)-2,3,4,5-tetrahydro-1H-benzo[d]azepin-7-ol* (**17b**): Synthesized according to general procedure H. Off-white solid. Yield: 54%. mp: 134.8–136.0 °C.

^1^H NMR (600 MHz, CDCl_3_) δ 6.97 (dd, *J* = 14.1, 7.5 Hz, 2H), 6.82 (s, 1H), 6.78 (d, *J* = 3.1 Hz, 1H), 6.77 (s, 1H), 5.72 (s, 1H), 5.66 (s, 1H), 4.75 (d, *J* = 9.7 Hz, 1H), 4.12 (d, *J* = 10.6 Hz, 1H), 3.50–3.39 (m, 2H), 3.20–3.13 (m, 3H), 3.01–2.95 (m, 1H), 2.67 (s, 3H), 2.49 (s, 3H) ppm. ^13^C NMR (150 MHz, CDCl_3_) δ 138.6, 137.3, 132.8, 129.6, 129.3, 128.9, 128.7, 128.0, 127.8, 125.6, 119.9, 114.2, 68.0, 61.1, 59.6, 42.9, 25.6, 21.5 ppm. HRESIMS calculated for C_18_H_22_ClN_2_O [M + H]^+^ 317.1342, found 317.1350.

*N-(9-chloro-8-hydroxy-3-methyl-5-phenyl-2,3,4,5-tetrahydro-1H-benzo[d]azepin-7-yl)acetamide* (**18a**): Synthesized according to general procedure I. White solid. Yield: 78%. mp: 154.4–156.2 °C.

^1^H NMR (600 MHz, Acetone-d6) δ 7.41 (s, 1H), 7.32 (dd, *J* = 13.6, 7.0 Hz, 2H), 7.28–7.22 (m, 2H), 7.07–7.03 (m, 3H), 4.29 (d, *J* = 10.5 Hz, 1H), 3.61–59 (m, 1H), 3.51–3.47 (m, 1H), 3.40–3.37 (m, 1H), 3.29–3.26 (m, 2H), 2.99–2.91 (m, 1H), 2.77 (s, 3H), 2.59 (s, 3H) ppm. ^13^C NMR (150 MHz, CDCl_3_) δ 170.2, 129.2, 129.1, 128.6, 128.5, 127.6, 127.3, 124.3, 124.2, 123.4, 119.7, 65.7, 59.0, 42.9, 29.7, 26.6, 23.8 ppm. HRESIMS calculated for C_19_H_22_ClN_2_O_2_ [M + H]^+^ 345.1292, found 345.1244.

*N-(9-chloro-8-hydroxy-3-methyl-5-(m-tolyl)-2,3,4,5-tetrahydro-1H-benzo[d]azepin-7-yl)acetamide* (**18b**): Synthesized according to general procedure I. Off-white solid. Yield: 68%, mp: 151.6–152.8 °C.

^1^H NMR (400 MHz, CDCl_3_) δ 7.26–7.19 (m, 1H), 7.17–7.04 (m, 1H), 6.90–6.83 (m, 2H), 6.67 (dd, *J* = 8.7, 4.0 Hz, 1H), 6.43 (d, *J* = 8.7 Hz, 1H), 6.32 (d, *J* = 8.7 Hz, 1H), 5.51 (s, 1H), 4.75 (d, *J* = 9.6 Hz, 1H), 4.41 (d, *J* = 10.6 Hz, 1H), 4.07–4.03 (m, 1H), 3.65 (d, *J* = 10.4 Hz, 1H), 3.60–3.51 (m, 2H), 3.42–3.32 (m, 1H), 3.31–3.14 (m, 2H), 3.05 (s, 3H), 2.88 (s, 3H), 2.29 (d, *J* = 12.0 Hz, 3H) ppm. ^13^C NMR (100 MHz, CDCl_3_) δ 150.3, 141.5, 140.7, 140.9, 138.9, 135.8, 135.2, 129.1, 128.6, 128.3, 125.4, 120.2, 114.0, 64.8, 62.5, 58.5, 55.8, 42.6, 25.4, 21.5 ppm. HRESIMS calculated for C_20_H_24_ClN_2_O_2_ [M + H]^+^ 359.1448, found 359.1430.

*N-(9-chloro-8-hydroxy-3-methyl-5-phenyl-2,3,4,5-tetrahydro-1H-benzo[d]azepin-7-yl)butyramide* (**19a**): Synthesized according to general procedure I. White solid. Yield: 82%. mp: 148.7–150.1 °C.

^1^H NMR (500 MHz, Acetone-d6) δ 7.44 (td, *J* = 7.3, 2.7 Hz, 3H), 7.39–7.36 (m, 1H), 7.30 (dd, *J* = 7.6, 2.1 Hz, 2H), 7.25 (d, *J* = 7.3 Hz, 1H), 5.09 (dd, *J* = 49.6, 9.5 Hz, 1H), 2.88 (s, 3H), 2.71 (d, *J* = 13.0 Hz, 2H), 2.65 (t, *J* = 7.3 Hz, 2H), 2.36 (t, *J* = 6.1 Hz, 1H), 1.75 (dd, *J* = 14.9, 7.3 Hz, 1H), 1.65 (dd, *J* = 14.8, 7.5 Hz, 2H), 1.04 (t, *J* = 9.3 Hz, 2H), 0.91 (t, *J* = 7.5 Hz, 3H) ppm. ^13^C NMR (150 MHz, CDCl_3_) δ 170.6, 143.1, 129.3, 129.2, 129.1, 128.6, 127.5, 127.2, 124.2, 123.6, 119.7, 65.6, 58.8, 38.9, 35.5, 26.1, 19.1, 18.3, 13.8 ppm. HRESIMS calculated for C_21_H_26_ClN_2_O_2_ [M + H]^+^ 373.1605, found 373.1649.

*N-(9-chloro-8-hydroxy-3-methyl-5-(m-tolyl)-2,3,4,5-tetrahydro-1H-benzo[d]azepin-7-yl)butyramide* (**19b**): Synthesized according to general procedure I. White solid. Yield: 63%. mp: 156.2–157.9 °C.

^1^H NMR (500 MHz, CDCl_3_) δ 7.39–7.30 (m, 1H), 7.18 (dd, *J* = 17.9, 7.7 Hz, 1H), 7.06–6.94 (m, 2H), 5.02 (dd, *J* = 30.1, 9.4 Hz, 1H), 3.79–3.56 (m, 2H), 3.53–3.46 (m, 1H), 3.42–3.19 (m, 3H), 2.93 (d, *J* = 5.7 Hz, 1H), 2.87 (d, *J* = 5.0 Hz, 1H), 2.68 (s, 2H), 2.39 (s, 3H), 1.93–1.78 (m, 3H), 1.09 (t, *J* = 7.2 Hz, 3H); ^1^H NMR (125 MHz, CDCl_3_) δ 170.6, 143.0, 141.1, 139.2, 138.8, 129.8, 129.6, 129.3, 128.9, 128.6, 128.3, 125.4, 119.8, 65.7, 59.1, 42.9, 38.8, 35.7, 26.6, 21.6, 18.4, 13.7 ppm. HRESIMS calculated for C_22_H_28_ClN_2_O_2_ [M + H]^+^ 387.1841, found 387.1843.

*N-(9-chloro-8-hydroxy-3-methyl-5-phenyl-2,3,4,5-tetrahydro-1H-benzo[d]azepin-7-yl)isobutyramide* (**20a**): Synthesized according to general procedure I. White solid. Yield: 76%. mp: 144.6–145.9 °C.

^1^H NMR (500 MHz, CDCl_3_) δ 7.45 (dd, *J* = 12.1, 5.2 Hz, 2H), 7.37 (dd, *J* = 13.4, 6.1 Hz, 1H), 7.29 (s, 1H), 7.23–7.18 (m, 2H), 5.10 (d, *J* = 9.5 Hz, 1H), 3.75 (dd, *J* = 17.9, 11.1 Hz, 1H), 3.53–3.44 (m, 1H), 3.38 (dd, *J* = 19.2, 12.4 Hz, 2H), 3.30–3.20 (m, 1H), 2.99–2.93 (m, 1H), 2.88 (s, 1H), 2.68 (s, 3H), 1.40 (d, *J* = 7.0 Hz, 6H) ppm. ^13^C NMR (125 MHz, CDCl_3_) δ 174.0, 141.1, 129.3, 129.2, 128.7, 128.7, 128.5, 127.8, 127.5, 127.3, 65.5, 58.9, 34.4, 31.7, 26.7, 22.8, 19.0 ppm. HRESIMS calculated for C_21_H_26_ClN_2_O_2_ [M + H]^+^ 373.1605, found 373.1634.

*N-(9-chloro-8-hydroxy-3-methyl-5-phenyl-2,3,4,5-tetrahydro-1H-benzo[d]azepin-7-yl)acrylamide* (**21a**): Synthesized according to general procedure I. Grayish solid. Yield: 81%. mp: 161.4–163.1 °C.

^1^H NMR (600 MHz, CDCl_3_) δ 7.31 (d, *J* = 7.8 Hz, 2H), 7.26 (d, *J* = 7.3 Hz, 2H), 7.19 (s, 1H), 7.07–7.01 (m, 2H), 6.32 (d, *J* = 17.6 Hz, 1H), 6.05 (d, *J* = 9.7 Hz, 1H), 5.78 (d, *J* = 10.3 Hz, 1H), 5.02–4.85 (m, 1H), 3.65 (t, *J* = 5.8 Hz, 1H), 3.58 (dd, *J* = 14.9, 8.1 Hz, 1H), 3.54–3.45 (m, 1H), 3.38 (dd, *J* = 16.2, 8.3 Hz, 1H), 3.31–3.14 (m, 2H), 2.76 (s, 1H), 2.57 (s, 3H) ppm. ^13^C NMR (150 MHz, CDCl_3_) δ 170.3, 143.0, 135.7, 131.4, 129.5, 129.4, 129.1, 128.6, 127.5, 127.2, 125.5, 124.3, 124.1, 67.9, 59.0, 42.9, 34.2, 25.6 ppm. HRESIMS calculated for C_20_H_22_ClN_2_O_2_ [M + H]^+^ 357.1292, found 357.1274.

*N-(9-chloro-8-hydroxy-3-methyl-5-phenyl-2,3,4,5-tetrahydro-1H-benzo[d]azepin-7-yl)methanesulfonamide* (**22**): Synthesized according to general procedure I. Gray solid. Yield: 40%. mp: 149.6–152.6 °C.

^1^H NMR (400 MHz, DMSO-d6) δ 7.48 (d, *J* = 6.9 Hz, 2H), 7.42 (d, *J* = 6.6 Hz, 1H), 7.27 (d, *J* = 6.7 Hz, 3H), 6.44 (s, 1H), 4.77 (d, *J* = 9.6 Hz, 1H), 3.80 (d, *J* = 11.8 Hz, 2H), 3.68–3.55 (m, 2H), 3.49–3.37 (m, 2H), 3.08 (s, 1H), 2.87 (s, 3H), 2.07 (d, *J* = 5.4 Hz, 3H). ^13^C NMR (100 MHz, DMSO-d6) δ 146.0, 140.5, 137.0, 136.2, 129.6, 129.0, 128.0, 122.5, 121.6, 120.0, 59.5, 53.7, 44.7, 44.3, 31.1, 27.2 ppm. HRESIMS calculated for C_18_H_22_ClN_2_O_3_S [M + H]^+^ 381.0961, found 381.0961.

*1-(9-chloro-8-hydroxy-3-methyl-5-phenyl-2,3,4,5-tetrahydro-1H-benzo[d]azepin-7-yl)urea* (**23a**): Synthesized according to general procedure I. White solid. Yield: 76%. mp: 141.0–142.1 °C.

^1^H NMR (400 MHz, DMSO-d6) δ 10.95 (s, 1H), 8.39 (s, 1H), 7.44 (d, *J* = 1.9 Hz, 1H), 7.43 (d, *J* = 1.9 Hz, 1H), 7.41 (s, 1H), 7.35 (d, *J* = 1.4 Hz, 1H), 7.33 (s, 1H), 7.22 (d, *J* = 1.5 Hz, 1H), 7.20–7.19 (m, 1H), 6.25 (s, 1H), 4.85 (d, *J* = 9.5 Hz, 1H), 3.50–3.40 (m, 1H), 3.39–3.35 (m, 1H), 3.30 (dd, *J* = 12.8, 2.5 Hz, 1H), 3.22–3.17 (m, 1H), 3.06 (d, *J* = 12.3 Hz, 1H), 2.79 (s, 1H), 2.58 (s, 3H) ppm. ^13^C NMR (100 MHz, DMSO-d6) δ 158.4, 143.1, 142.4, 132.8, 129.4, 129.0, 127.9, 127.4, 126.8, 123.0, 118.8, 66.9, 60.0, 44.0, 42.1, 26.3 ppm. HRESIMS calculated for C_18_H_21_ClN_3_O_2_ [M + H]^+^ 346.1244, found 346.1241

*1-(9-chloro-8-hydroxy-3-methyl-5-(m-tolyl)-2,3,4,5-tetrahydro-1H-benzo[d]azepin-7-yl)urea* (**23b**): Synthesized according to general procedure I. White solid. Yield: 54%. mp: 184.6–186.1 °C.

^1^H NMR (500 MHz, Acetone-d6) δ 8.21 (s, 2H), 7.37–7.30 (m, 1H), 7.22–7.14 (m, 2H), 7.08 (s, 1H), 7.03 (d, *J* = 7.5 Hz, 1H), 4.83 (d, *J* = 11.4 Hz, 1H), 3.65–3.54 (m, 3H), 3.33–3.24 (m, 3H), 2.89 (s, 3H), 2.37 (s, 3H) ppm; ^13^C NMR (125 MHz, DMSO-d6) δ 157.8, 141.8, 141.5, 137.9, 137.3, 131.7, 128.7, 128.3, 127.8, 127.5, 126.5, 126.3, 122.2, 33.9, 30.9, 28.4, 24.7, 24.1, 21.1 ppm. HRESIMS calculated for C_19_H_23_ClN_3_O_2_ [M + H]^+^ 360.2201, found 360.2200.

*6-chloro-8-nitro-1-phenyl-2,3,4,5-tetrahydro-1H-benzo[d]azepin-7-ol* (**24a**): Synthesized according to general procedure G. Yellow solid. Yield: 74%. mp: 201.4–202.8 °C.

^1^H NMR (600 MHz, CDCl_3_) δ 11.15 (s, 1H), 7.67 (s, 1H), 7.42 (t, *J* = 7.6 Hz, 1H), 7.39 (s, 1H), 7.19 (d, *J* = 7.7 Hz, 2H), 7.08 (d, *J* = 7.5 Hz, 2H), 4.94 (dd, *J* = 14.3, 8.1 Hz, 1H), 4.86 (dd, *J* = 14.3, 4.0 Hz, 1H), 4.64–4.58 (m, 1H), 4.48–4.44 (m, 1H), 3.99–3.94 (m, 1H), 3.54 (ddd, *J* = 15.4, 7.7, 3.3 Hz, 1H), 3.40 (ddd, *J* = 15.4, 8.8, 3.4 Hz, 1H) ppm. ^13^C NMR (150 MHz, CDCl_3_) δ 140.9, 136.3, 135.3, 131.9, 127.9, 127.6, 126.1, 124.4, 118.9, 113.2, 64.8, 58.6, 42.0, 25.3, 24.7 ppm. HRESIMS calculated for C_16_H_16_ClN_2_O_3_ [M + H]^+^ 319.0771, found 319.0800.

*6-chloro-8-nitro-1-(m-tolyl)-2,3,4,5-tetrahydro-1H-benzo[d]azepin-7-ol* (**24b**): Synthesized according to general procedure G. Yellow solid. Yield: 80%. mp: 188.9–191.2 °C.

^1^H NMR (600 MHz, Acetone-d6) δ 7.37 (d, *J* = 7.5 Hz, 1H), 7.31 (dd, *J* = 15.6, 8.1 Hz, 2H), 7.23 (s, 1H), 7.22 (s, 1H), 4.96 (d, *J* = 9.8 Hz, 1H), 3.98–3.89 (m, 2H), 3.86–3.76 (m, 2H), 3.73–3.66 (m, 1H), 3.65–3.56 (m, 2H), 3.00 (s, 3H) ppm. ^13^C NMR (150 MHz, Acetone-d6) δ 150.6, 146.6, 140.1, 139.4, 133.5, 129.7, 129.6, 129.0, 125.9, 124.5, 122.5, 121.7, 60.8, 54.4, 45.3, 28.4, 21.1 ppm. HRESIMS calculated for C_17_H_18_ClN_2_O_3_ [M + H]^+^ 333.0298, found 333.0276.

### 4.11. Biological Experimental Procedures

#### 4.11.1. Dopamine Receptor Binding Assays

D_1_R, D_2_R and D_5_R binding assays were performed by the Psychoactive Drug Screening Program (PDSP). Briefly, compounds were tested initially in primary radioligand binding assays at each receptor. In the primary binding assays, the compounds were tested at a concentration of 10 μM in quadruplicate in 96-well plates. Compounds that displayed a minimum of 50% inhibition in the primary assays were advanced to secondary binding assays to determine K_i_ values at each receptor. In secondary binding assays, compounds were tested at 11 concentrations (0.1, 0.3, 1, 3, 10, 30, 100, 300 nM, 1, 3, 10 µM) and in triplicate. Both primary and secondary binding assays were carried out in a final assay volume of 125 μL per well in binding buffer (50 mM HEPES, 50 mM NaCl, 5 mM MgCl_2_, 0.5 mM EDTA, pH 7.4, rt; Standard Wash Buffer: 50 mM Tris HCl, pH 7.4, cold). The radioligand concentration chosen was close to the K_d_. Radioligands used for D_1_R, D_2_R and D_5_R were [^3^H]-SCH23390, [^3^H]-N-methylspiperone and [^3^H]-SCH23390, respectively, with K_d_ values of 0.74, 0.47 and 2.03, respectively. The total binding and nonspecific binding were determined in the absence and presence of the appropriate reference compound. Reference compounds for D_1_R, D_2_R and D_5_R assays were (+)-butaclamol, haloperidol and SKF 83586, respectively.

Complete details of the assays performed may be found online in the PDSP assay protocol book (http://pdsp.med.unc.edu/PDSP%20Protocols%20II%202013-03-28.pdf (accessed on 30 July 2023)).

#### 4.11.2. Dopamine Receptor Functional Assays

The D_1_R agonist and antagonist function of compound **10a** was evaluated in cAMP assays by Eurofins Discover X using their proprietary HitHunter platform. Briefly, cells were seeded in a total volume of 20 μL into white-walled microplates and incubated at 37 °C prior to testing. In the agonist format, cells were first incubated with sample to induce a response. Then, the media was aspirated from the cells and replaced with 15 μL 2:1 HBSS/10 mM Hepes: cAMP XS + Ab reagent. Sample stocks were diluted to generate 4X sample in assay buffer. A 5 μL volume of 4X sample was then added to cells and incubated at 37 °C for 30 min. The final assay concentration used was 1%. In antagonist mode, the cells were pre-incubated with a sample followed by agonist challenge at the EC_80_ concentration. The media was aspirated from cells and replaced with 10 μL 2:1 HBSS/10 mM Hepes: cAMP XS + Ab reagent. A 5 μL volume of 4X sample was then added to cells and incubated at 37 °C for 30 min. Subsequently, 5 μL of 4X EC_80_ agonist was added to the cells and incubated at 37 °C for 30 min. For agonist and antagonist assays, signal detection was performed as follows. After appropriate compound incubation, the assay signal was generated via incubation with 20 μL cAMP XS + ED/CL lysis cocktail for 1 h. This was followed by incubation with 20 μL cAMP XS + EA reagent for 3 h at room temperature. The microplates were read following signal generation with a PerkinElmer Envision™ instrument for chemiluminescent signal detection.

## Figures and Tables

**Table 1:** Binding affinities of series 1 analogs at D1R, D2R and D5R.

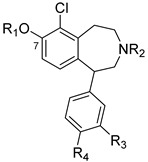								
					K_i_ (nM) ^a^			Selectivity
Cmpd #	R_1_	R_2_	R_3_	R_4_	D1R ^b^	D2R ^c^	D5R ^d^	D5/D1
**10a**	Me	H	H	H	na ^e^	na	na	
**11a**	H	H	H	H	na	na	470	
**12a**	Me	Allyl	H	H	na	na	na	
**13a**	Me	Me	H	H	na	na	na	
**14a**	H	Allyl	H	H	102 ± 5.2	na	297 ± 19.3	2.9
**15a**	H	Me	H	H	30.1 ± 1.3	na	187.4 ± 7.3	6.2
**10b**	Me	H	Me	H	na	na	na	
**11b**	H	H	Me	H	na	na	na	
**12b**	Me	Allyl	Me	H	na	na	na	
**13b**	Me	Me	Me	H	na	na	na	
**14b**	H	Allyl	Me	H	281.4 ± 9.4	na	740.6 ± 12.5	2.6
**15b**	H	Me	Me	H	77.9 ± 4.1	na	165.2 ± 4.9	2.1
**10c**	Me	H	H	OMe	na	na	na	
**12c**	Me	Allyl	H	OMe	na	na	na	
**11d**	H	H	H	OH	na	na	na	
**14d**	H	Allyl	H	OH	na	na	na	
**15d**	H	Me	H	OH	160.9 ± 5.5	na	412.9 ± 9.8	2.6
**(+)-Butaclamol**					4.0 ± 0.2			
**Haloperidol**						5.5 ± 0.3		
**SKF 83566**							3.9 ± 0.2	

^a^ Experiments carried out in triplicate; ^b^ [3H] SCH23390 used as radioligand; ^c^ [3H]N-methylspiperone used as radioligand; ^d^ [3H]SCH23390 used as radioligand; ^e^ na—not active (<50% inhibition in a primary assay when tested at 10 µM).

**Table 2:** Binding affinities of series 2 analogs at D1R, D2R and D5R.

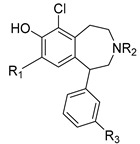							
					K_i_ (nM) ^a.^		Selectivity
Cmpd #	R_1_	R_2_	R_3_	D1R ^b^	D2R ^c^	D5R ^d^	D5/D1
**16a**	NO_2_	Me	H	na ^e^	na	na	
**16b**	NO_2_	Me	Me	4354.1 ± 67.3	na	na	
**17a**	NH_2_	Me	H	210.4 ± 7.1	na	269.8 ± 8.4	1.3
**17b**	NH_2_	Me	Me	71.9 ± 3.9	na	146.4 ± 5.8	2.0
**18a**	NHCOMe	Me	H	na	na	na	
**18b**	NHCOMe	Me	Me	1364.9 ± 14.0	na	na	
**19a**	NHCO(CH_2_)_3_Me	Me	H	1970.1 ± 41.4	na	na	
**19b**	NHCO(CH_2_)_3_Me	Me	Me	634.4 ± 12.5	na	2959.3 ± 29.6	4.7
**20a**	NHCOCH_2_CH(CH_3_)_2_	Me	H	2938.3 ± 36.2	na	na	
**20b**	NHCOCH_2_CH(CH_3_)_2_	Me	Me	4734.8 ± 55.9	na	na	
**21a**	NHCOCH=CH_2_	Me	H	1497.9 ± 18.9	na	na	
**21b**	NHCOCH=CH_2_	Me	Me	4979.6 ± 61.3	na	na	
**22**	NHSO_2_Me	Me	Me	113.7 ± 5.1	na	165.7 ± 6.8	1.5
**23a**	NHCONH_2_	Me	H	1761.9 ± 17.6	na	na	
**23b**	NHCONH_2_	Me	Me	na	na	na	
**24a**	NO_2_	H	H	na	na	na	
**24b**	NO_2_	H	Me	38.1 ± 2.0	na	97.9 ± 3.6	2.6
**(+)-Butaclamol**				4.0 ± 0.2			
**Haloperidol**					5.5 ± 0.3		
**SKF 83566**						3.9 ± 0.2	

^a^ Experiments carried out in triplicate; ^b^ [3H]SCH23390 used as radioligand; ^c^ [3H]N-methylspiperone used as radioligand; ^d^ [3H]SCH23390 used as radioligand; ^e^ na—not active (<50% inhibition in a primary assay when tested at 10 µM).

**Table 3:** Activity of **15a** in D1R cAMP functional assays.

Compound	Assay Format	Result	RC_50_ (nM)	Max Response
**Dopamine**	Agonist	EC_50_	12.30	103.01
**SCH 39166**	Antagonist	IC_50_	1.67	102.24
**15a**	Agonist	EC_50_	>10,000	17.22
**15a**	Antagonist	IC_50_	130.32	66.12

## Data Availability

^1^H NMR and ^13^C NMR spectral data for all tested analogs are available in the Supplementary Materials.

## Supplementary Materials

The following supporting information can be downloaded at: https://www.mdpi.com/article/10.3390/molecules28166010/s1.
