# Supplementary material for: Structure–Activity Relationship Studies on 6-Chloro-1-phenylbenzazepines Leads to the Identification of a New Dopamine D1 Receptor Antagonist
Source: Molecules. 2023 Aug 11;28(16):6010. doi: 10.3390/molecules28166010 (PMC10458514; doi:10.3390/molecules28166010)
Supplement: Supplementary file 1 [file molecules-28-06010-s001.zip › molecules-2541031-supplementary.pdf]

Supporting Information for

Structure-Activity Relationship Studies on 6-Chloro-1-phenylbenzazepines Leads to the Identification of a New Dopamine D1 Receptor Antagonist

Rajan Giri [1,2], Hari K. Namballa [1], Vishwashiv Emogaje [1] and Wayne W. Harding [1,2,3,*]

[1]*Department of Chemistry, Hunter College, City University of New York, 695 Park Avenue, NY 10065, USA*

[2]*Program in Chemistry, CUNY Graduate Center, 365 5th Avenue, New York, NY 10016, USA*

[3]*Program in Biochemistry, CUNY Graduate Center, 365 5th Avenue, New York, NY 10016, USA*

Table of Contents:

1. ^{1}H NMR and ^{13}C NMR spectra S2-S29

10a

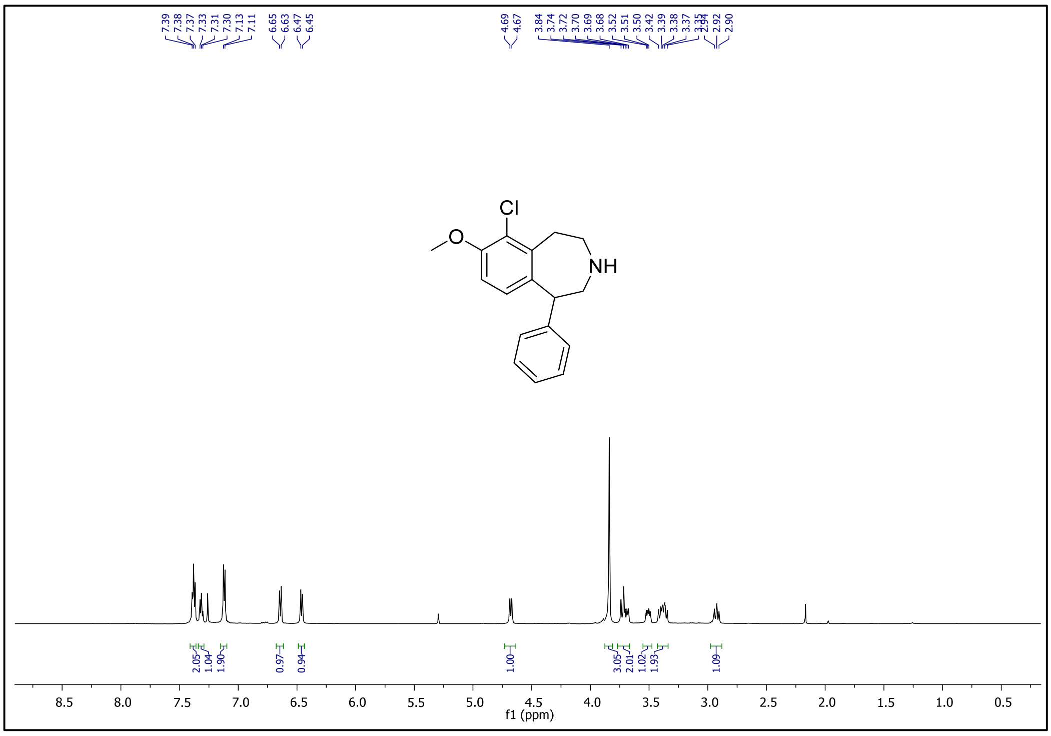

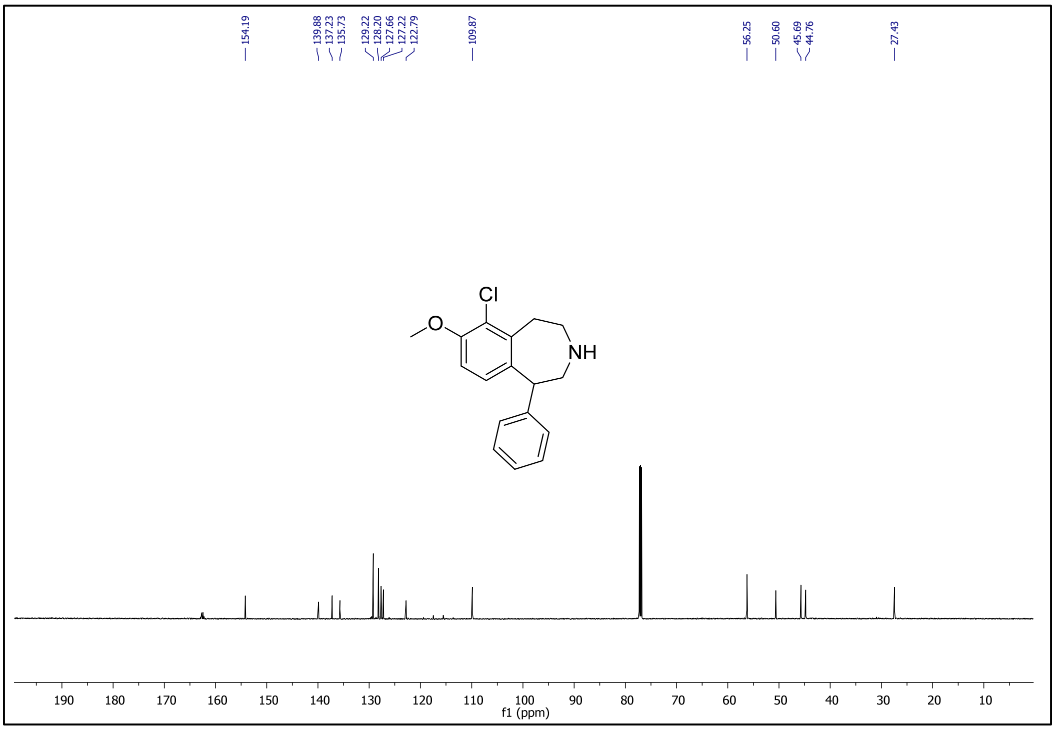

10b

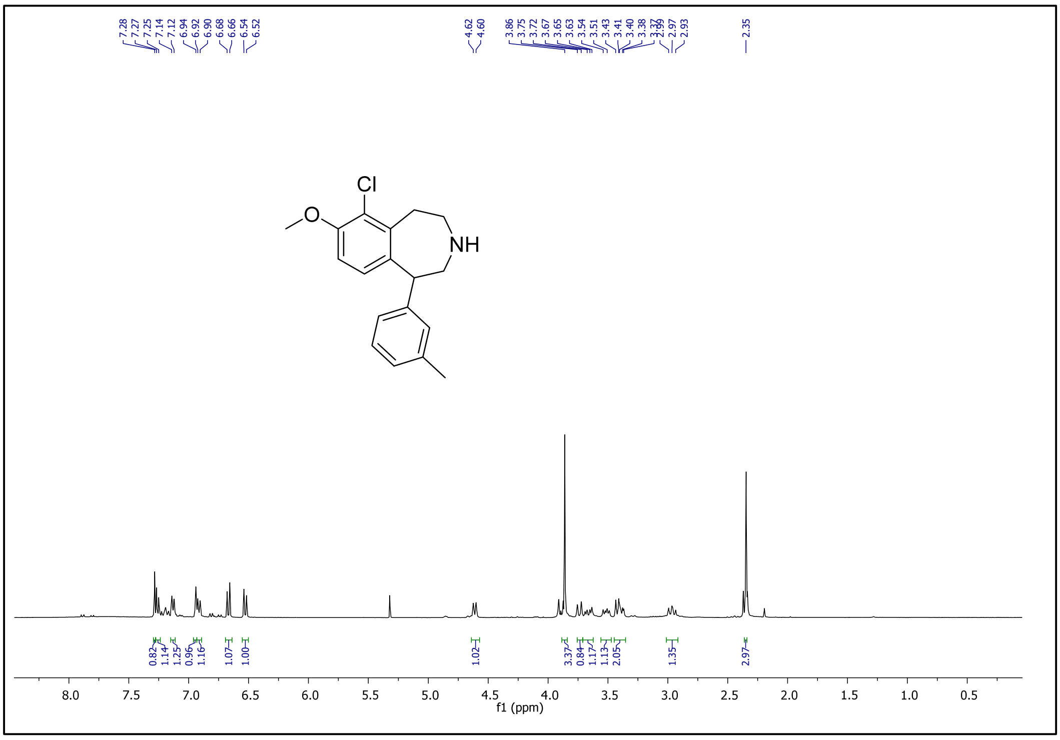

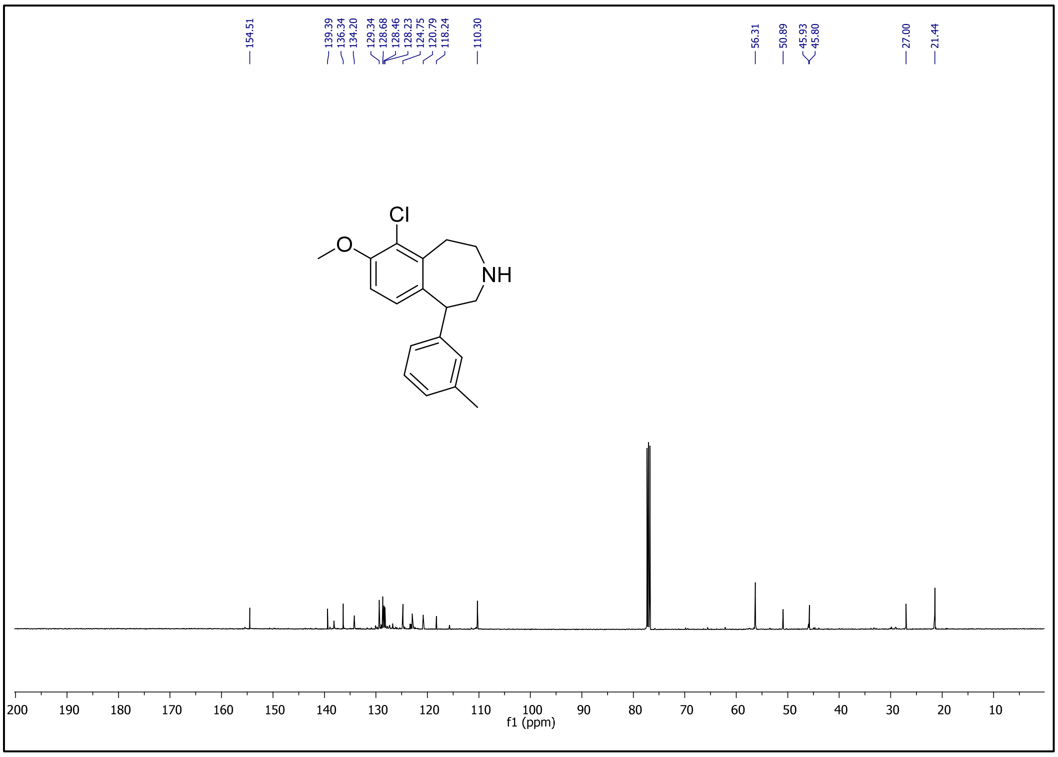

10c

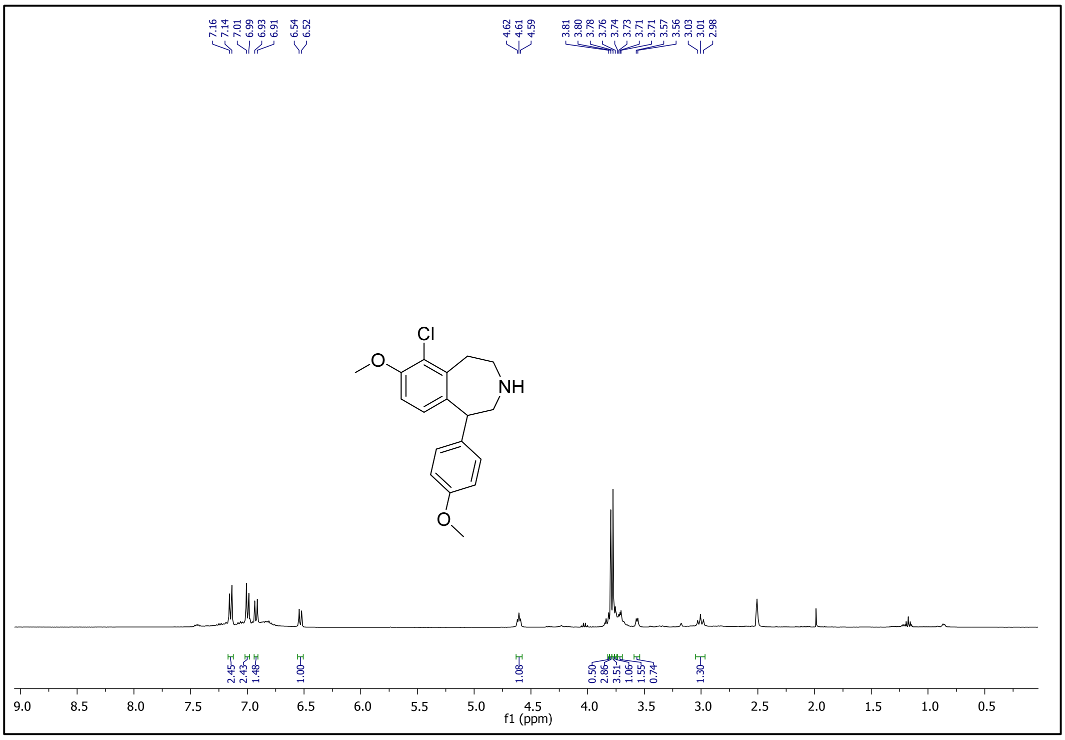

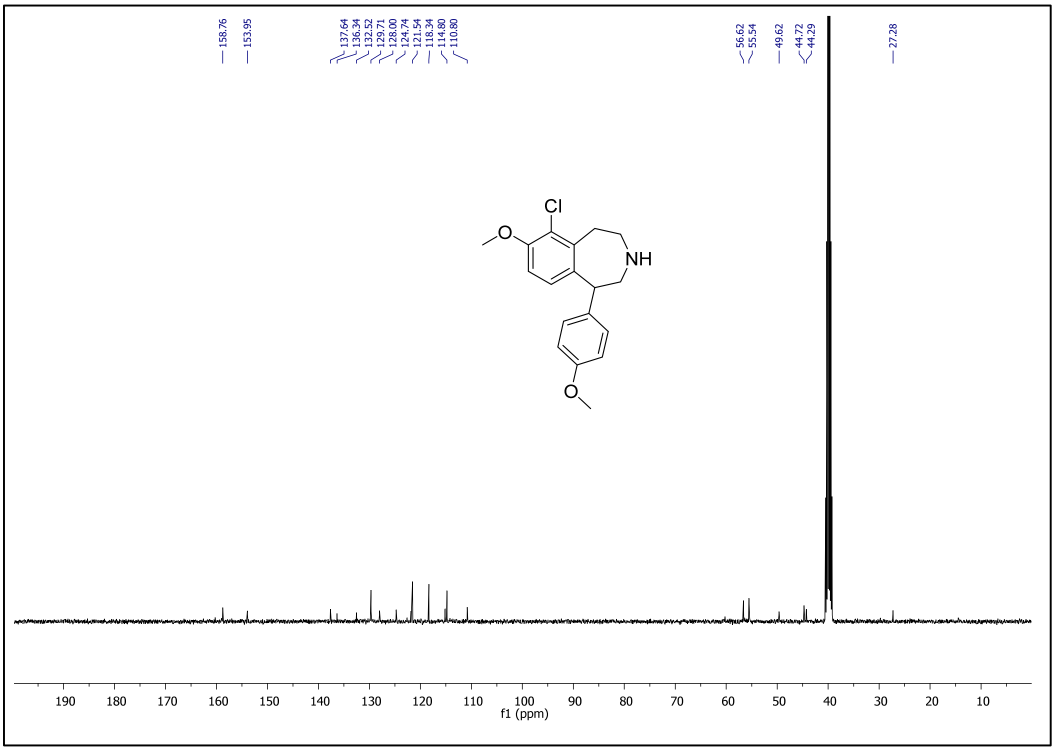

11b

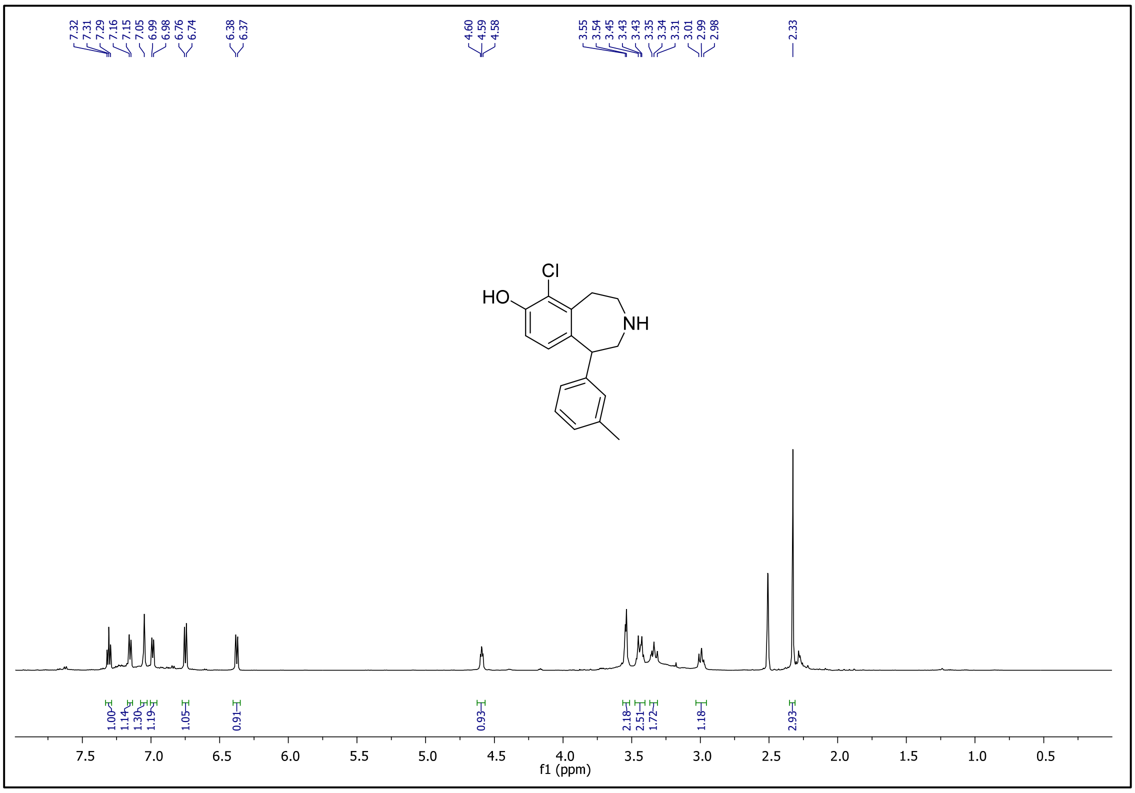
7.32
7.31
7.29
7.16
7.15
7.05
6.99
6.98
6.76
6.74
6.38
6.37
4.60
4.59
4.58
3.55
3.54
3.45
3.43
3.43
3.35
3.34
3.31
3.01
2.99
2.98
2.33
Cl
HO
NH
1.00
1.14
1.30
1.19
1.05
0.91
0.93
2.18
2.51
1.72
1.18
2.93
7.5 7.0 6.5 6.0 5.5 5.0 4.5 4.0 3.5 3.0 2.5 2.0 1.5 1.0 0.5
f1 (ppm)

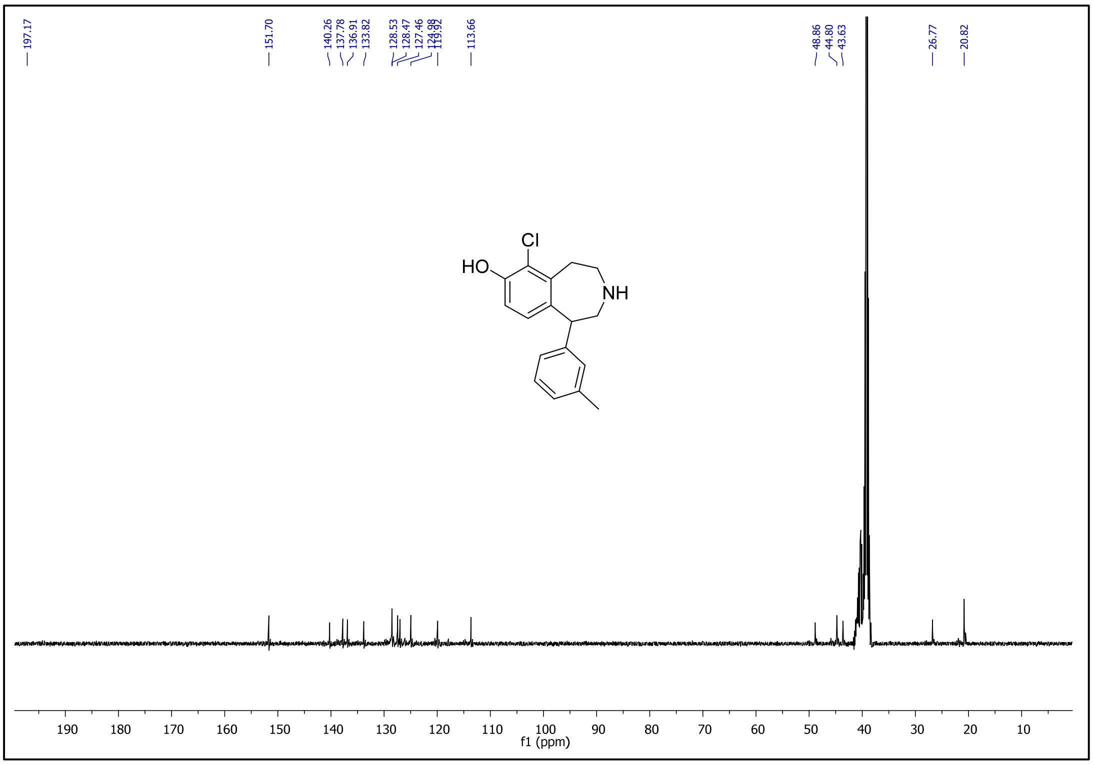
197.17
151.70
140.26
137.78
136.91
133.82
128.53
128.47
127.46
124.98
119.92
113.66
48.86
44.80
43.63
26.77
20.82
Cl
HO
NH
190 180 170 160 150 140 130 120 110 100 90 80 70 60 50 40 30 20 10
f1 (ppm)

11c

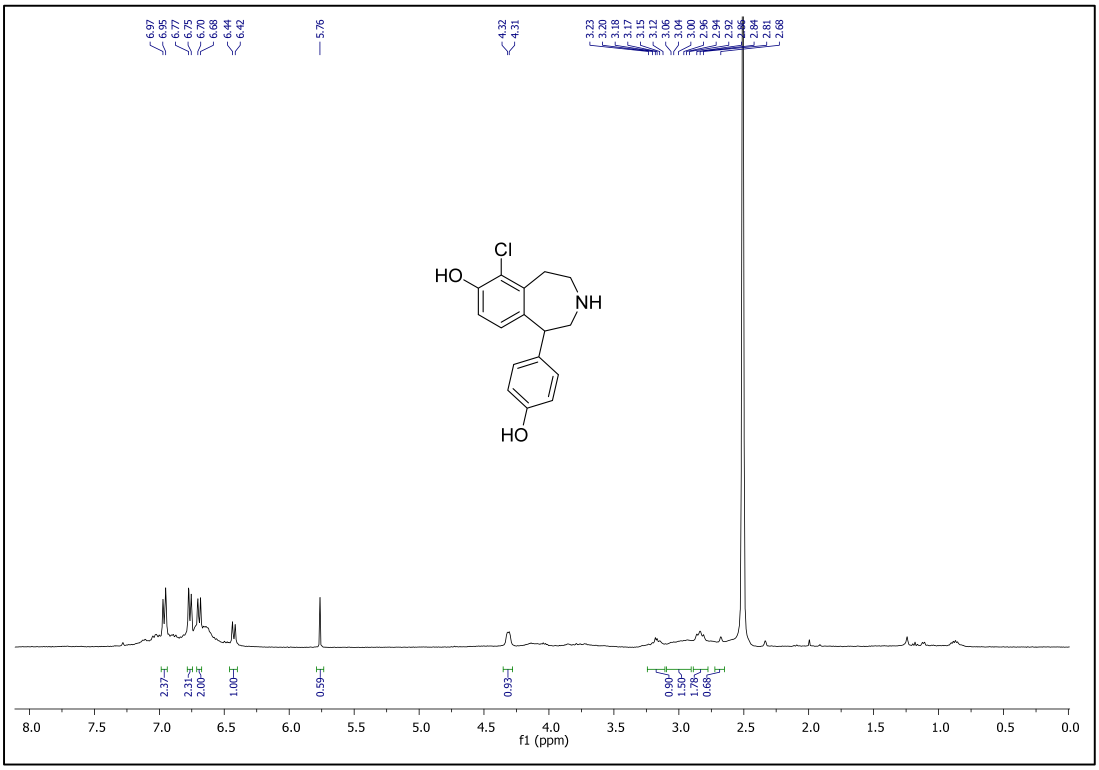
6.97
6.95
6.77
6.75
6.70
6.68
6.44
6.42
5.76
4.32
4.31
3.23
3.20
3.18
3.17
3.15
3.12
3.06
3.04
3.00
2.96
2.94
2.92
2.84
2.81
2.68
Cl
HO
NH
HO
2.37
2.31
2.00
1.00
0.59
0.93
0.90
1.50
1.78
0.68
8.0 7.5 7.0 6.5 6.0 5.5 5.0 4.5 4.0 3.5 3.0 2.5 2.0 1.5 1.0 0.5 0.0
f1 (ppm)

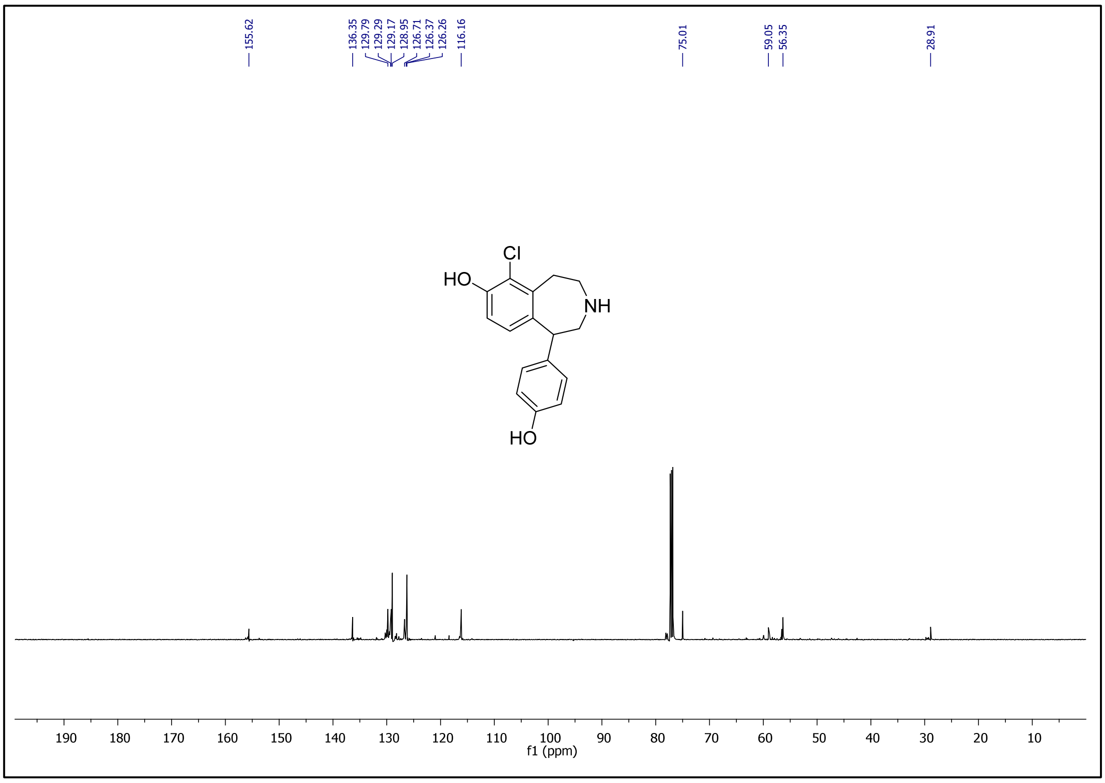
155.62
136.35
129.79
129.29
129.17
128.95
126.71
126.37
126.26
116.16
75.01
59.05
56.35
28.91
Cl
HO
NH
HO
190 180 170 160 150 140 130 120 110 100 90 80 70 60 50 40 30 20 10
f1 (ppm)

12b

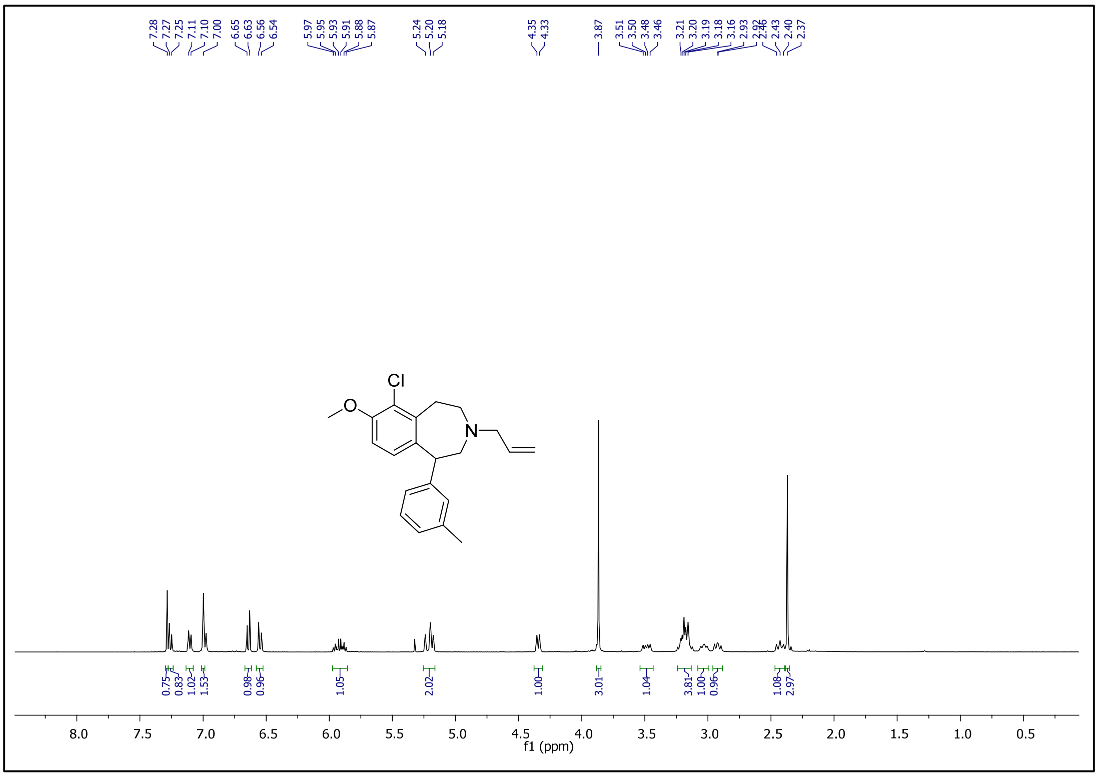

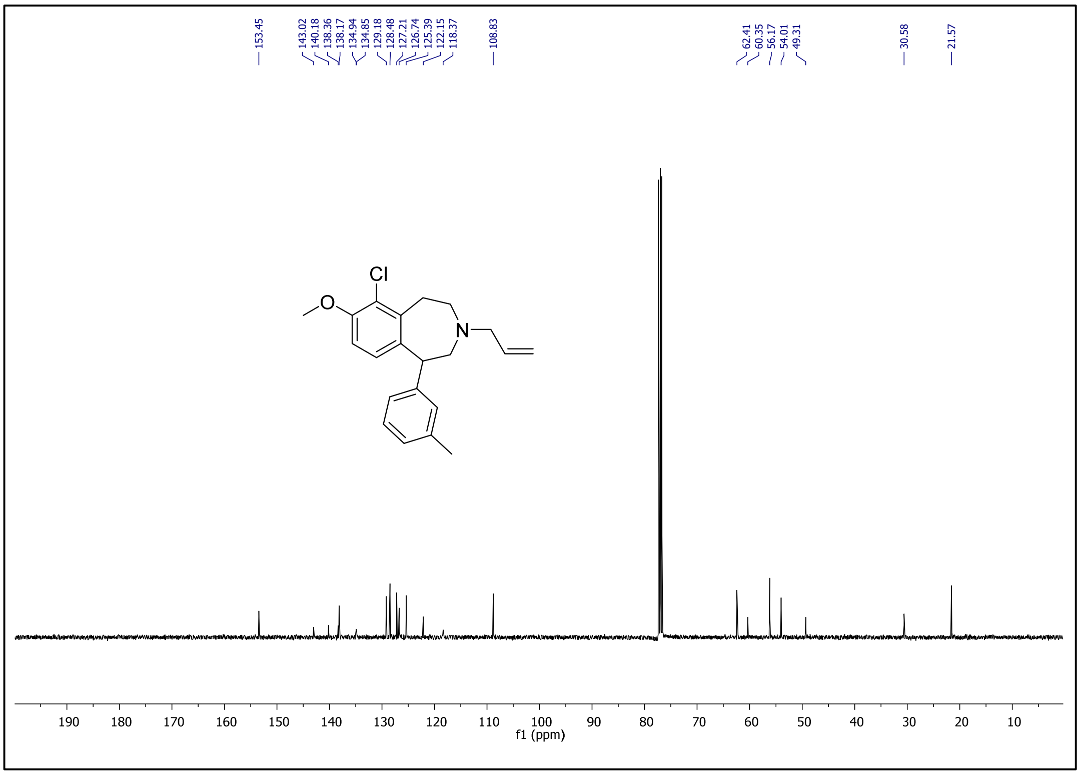

12c

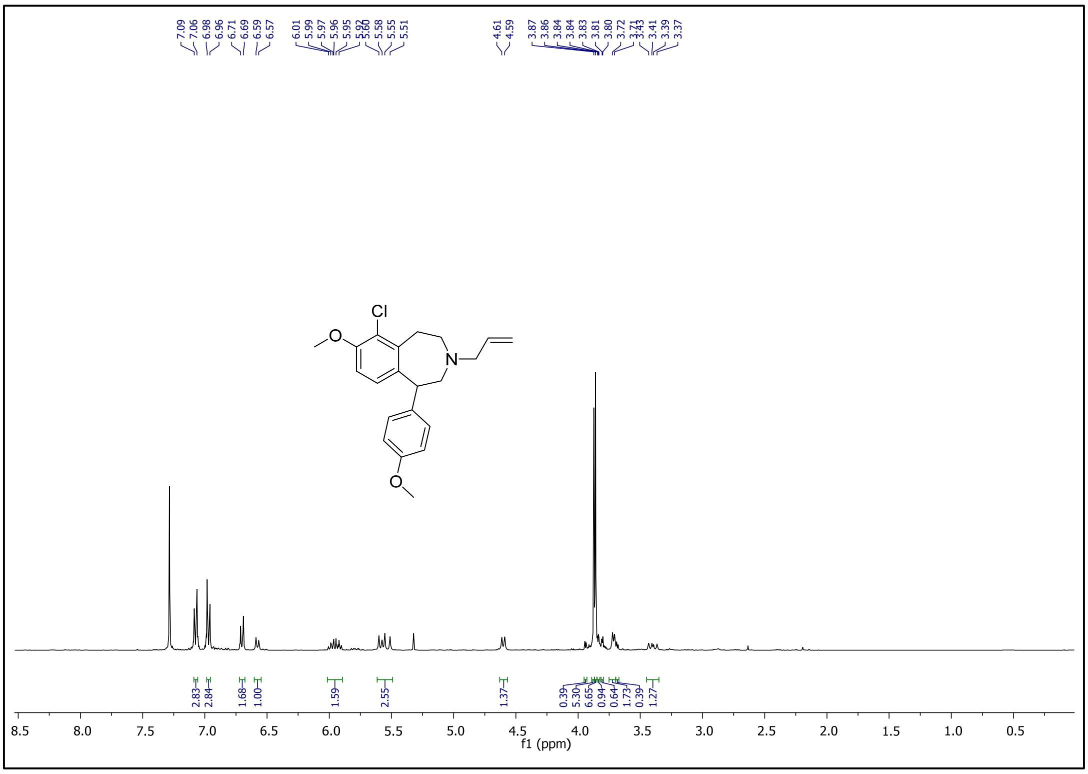
7.09
7.06
6.98
6.96
6.71
6.69
6.59
6.57
6.01
5.99
5.97
5.96
5.95
5.58
5.55
5.51
4.61
4.59
3.87
3.86
3.84
3.84
3.83
3.81
3.80
3.72
3.41
3.39
3.37
Cl
O
N
O
2.83
2.84
1.68
1.00
1.59
2.55
1.37
0.39
5.30
6.65
0.94
0.64
1.73
0.39
1.27
8.5
8.0
7.5
7.0
6.5
6.0
5.5
5.0
4.5
4.0
3.5
3.0
2.5
2.0
1.5
1.0
0.5
f1 (ppm)

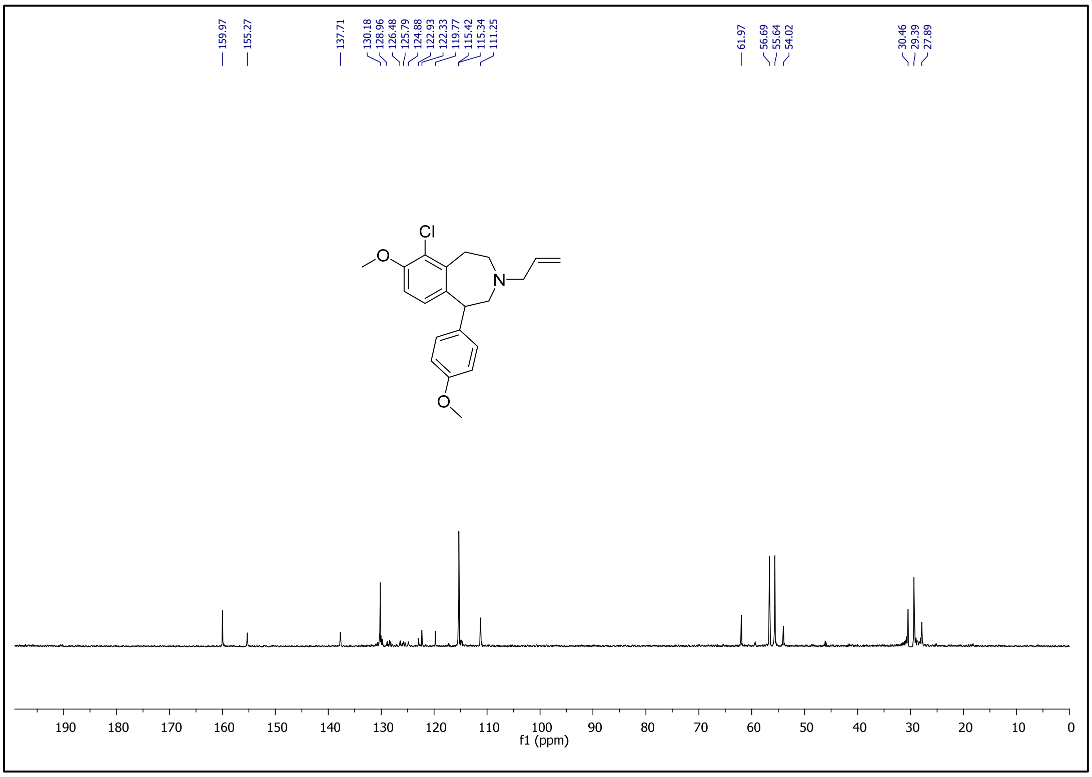
159.97
155.27
137.71
130.18
128.96
126.48
125.79
124.88
122.93
122.33
119.77
115.42
115.34
111.25
61.97
56.69
55.64
54.02
30.46
29.39
27.89
Cl
O
N
O
190
180
170
160
150
140
130
120
110
100
90
80
70
60
50
40
30
20
10
0
f1 (ppm)

13b

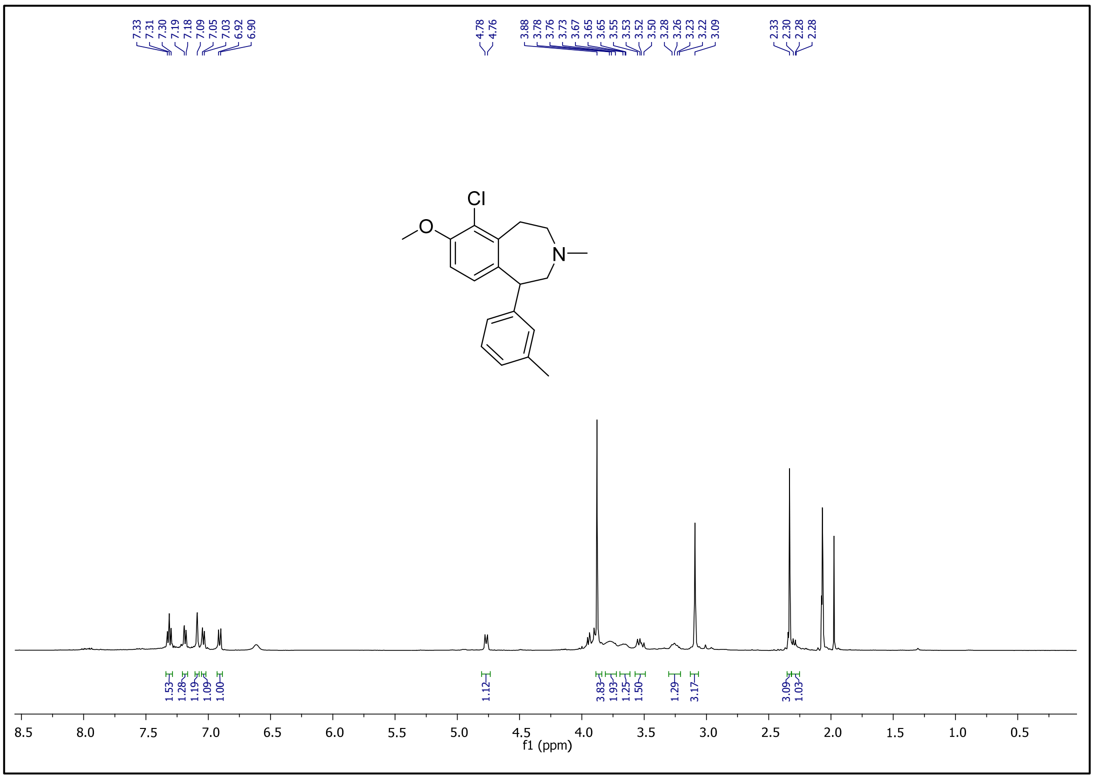
7.33
7.31
7.30
7.19
7.18
7.09
7.05
7.03
6.92
6.90
4.78
4.76
3.88
3.78
3.76
3.73
3.67
3.65
3.55
3.53
3.52
3.50
3.28
3.26
3.23
3.22
3.09
2.33
2.30
2.28
2.28
Cl
O
N
1.53
1.28
1.19
1.09
1.00
1.12
3.83
1.93
1.25
1.50
1.29
3.17
3.09
1.03
8.5
8.0
7.5
7.0
6.5
6.0
5.5
5.0
4.5
4.0
3.5
3.0
2.5
2.0
1.5
1.0
0.5
f1 (ppm)

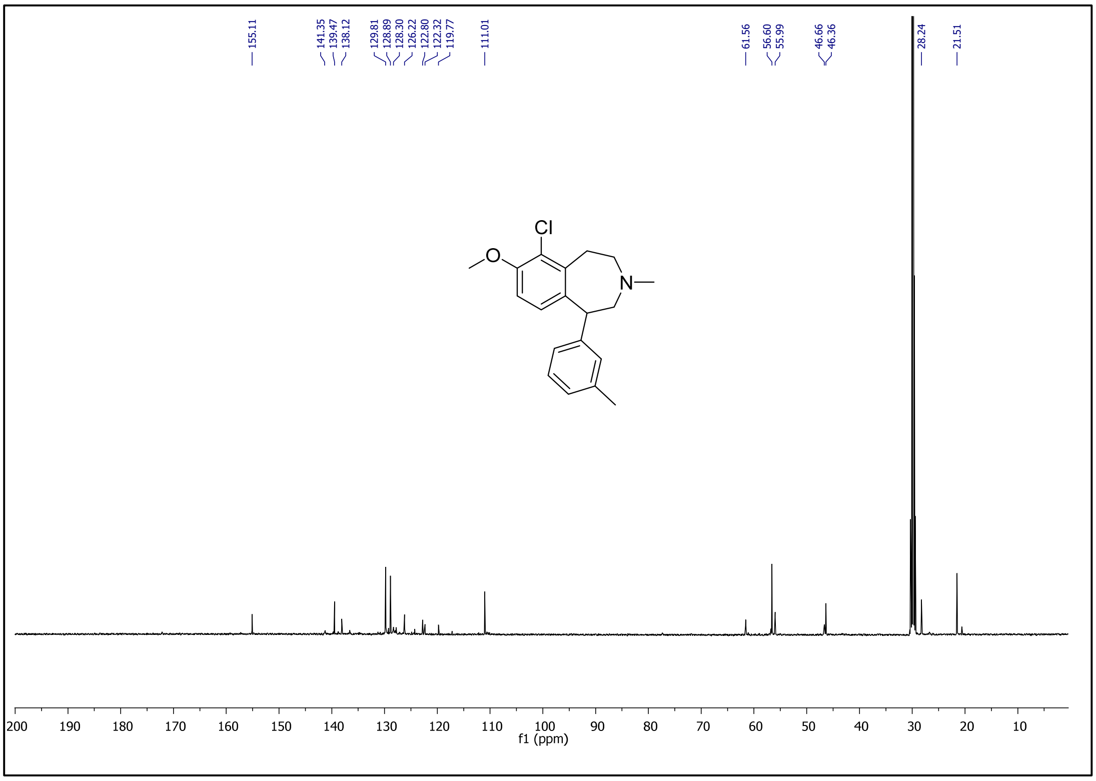
155.11
141.35
139.47
138.12
129.81
128.89
128.30
126.22
122.80
122.32
119.77
111.01
61.56
56.60
55.99
46.66
46.36
28.24
21.51
Cl
O
N
200
190
180
170
160
150
140
130
120
110
100
90
80
70
60
50
40
30
20
10
f1 (ppm)

13c

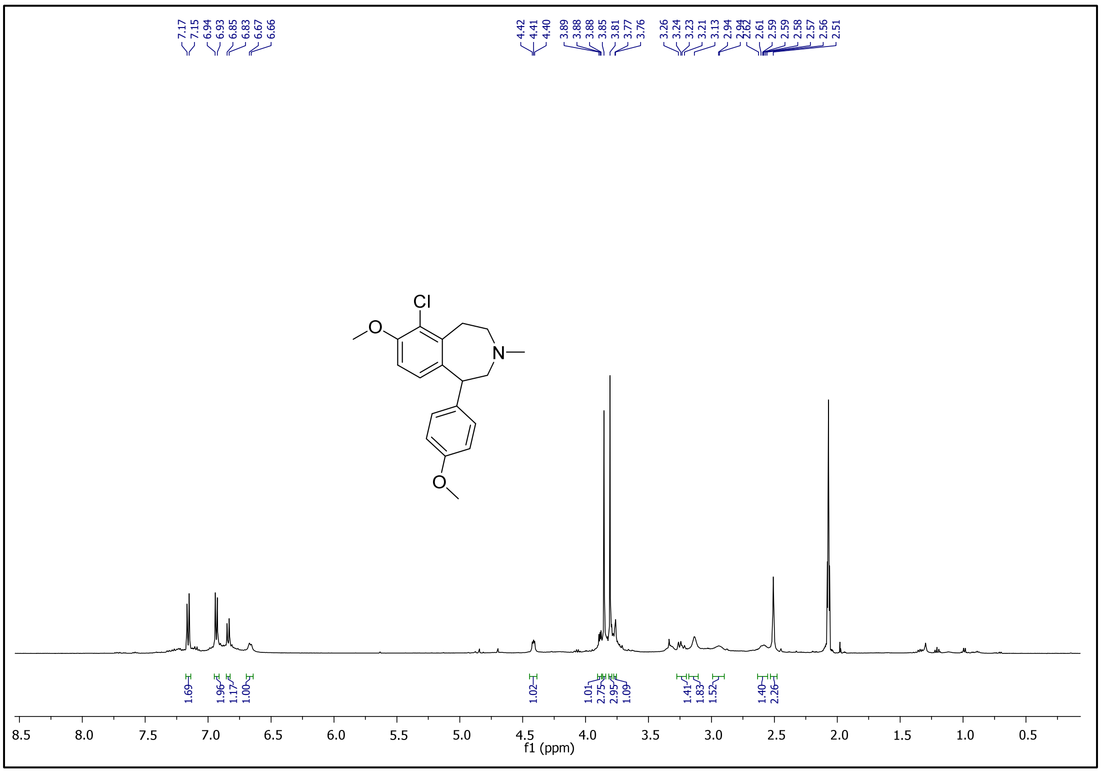

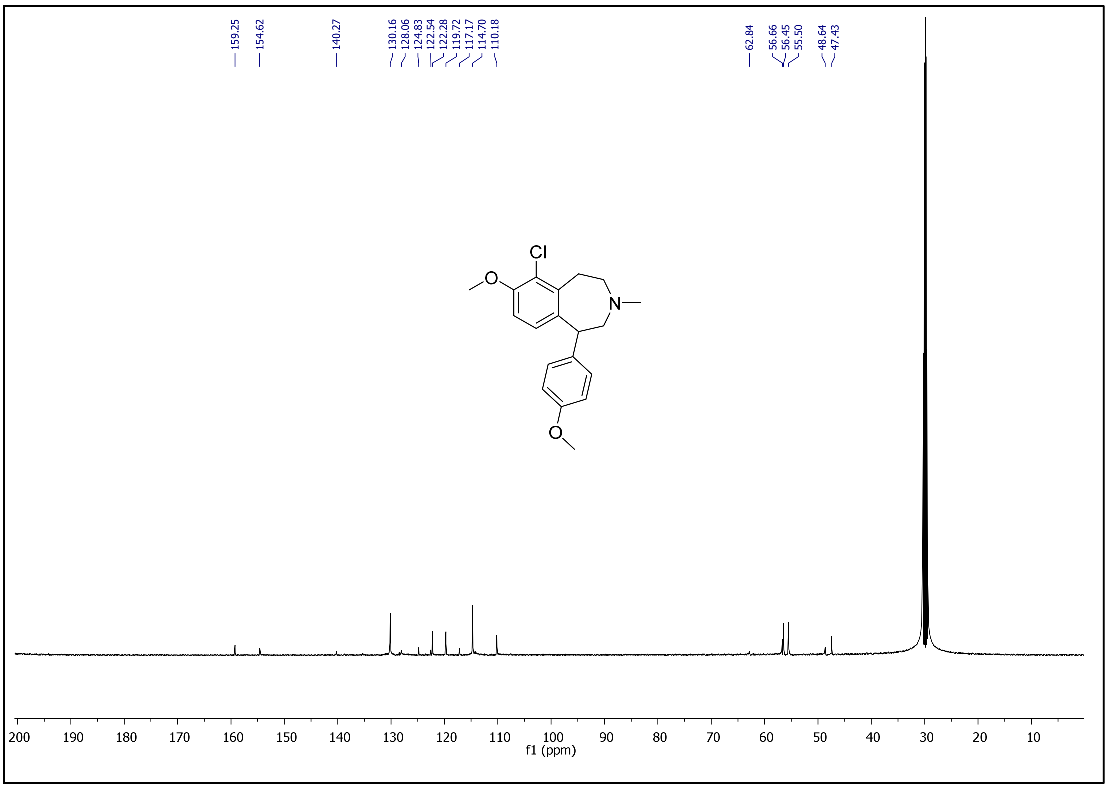

14b

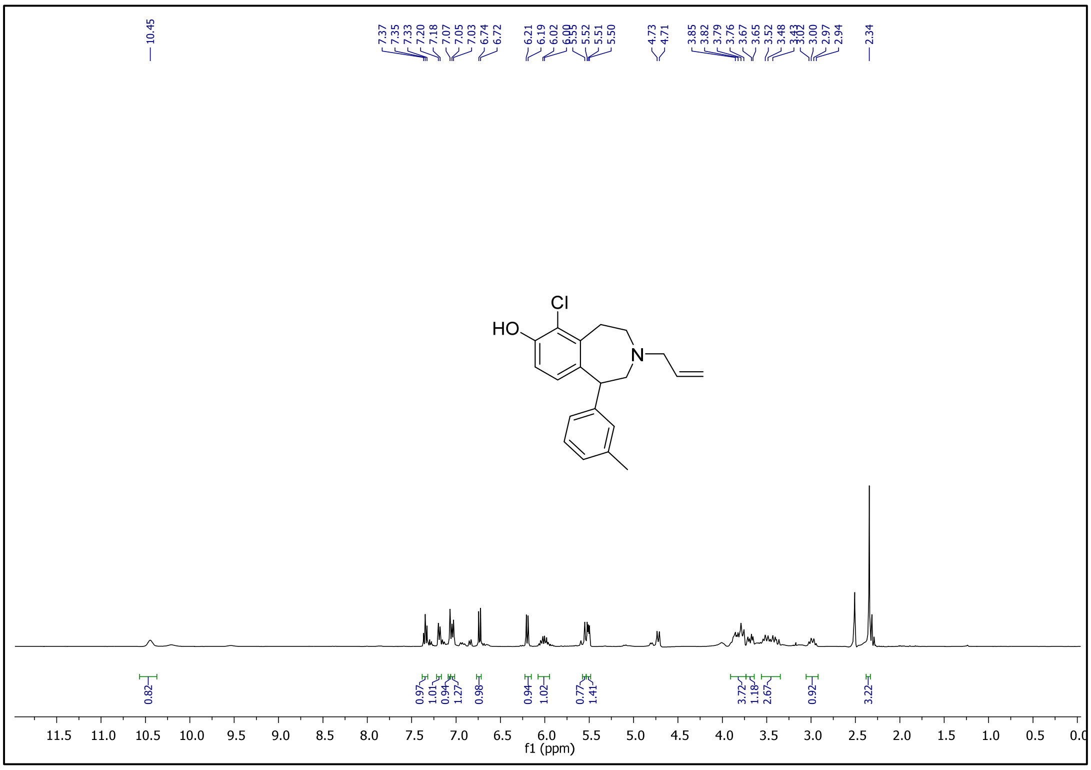

Cl
HO
N
f1 (ppm)

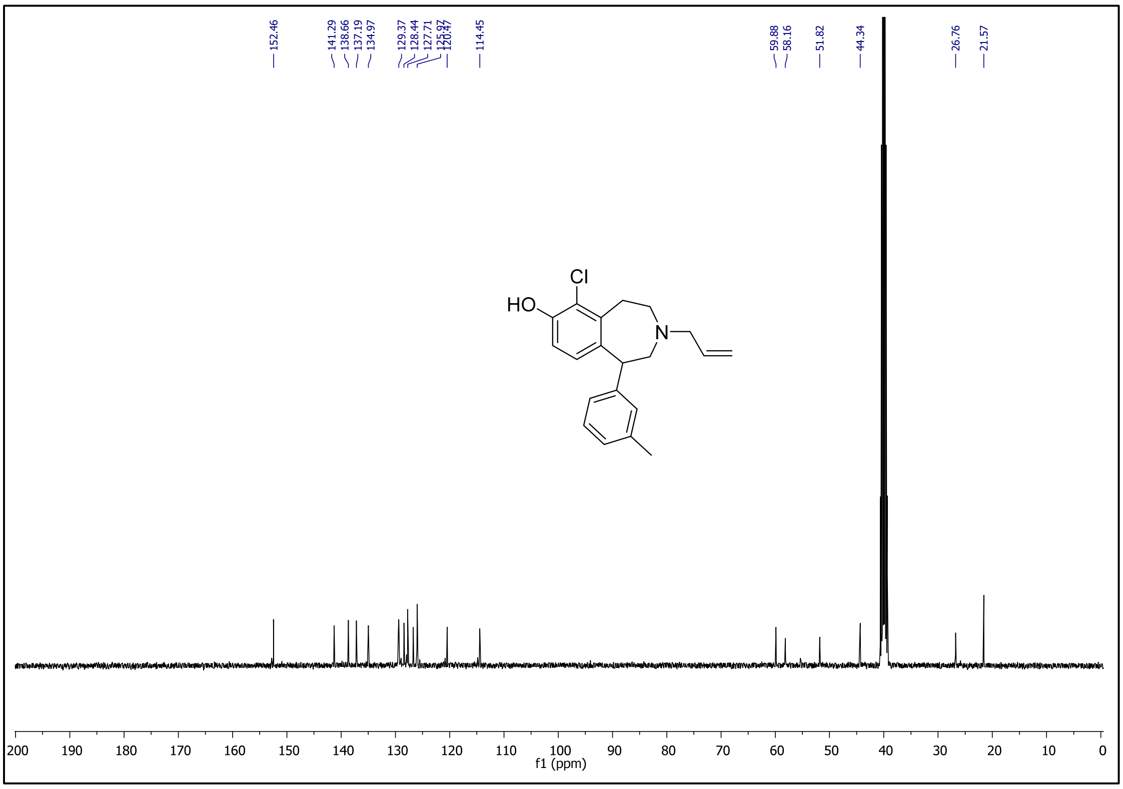

152.46
141.29
138.66
137.19
134.97
129.37
128.44
127.71
114.45
59.88
58.16
51.82
44.34
26.76
21.57
Cl
HO
N
f1 (ppm)

14d

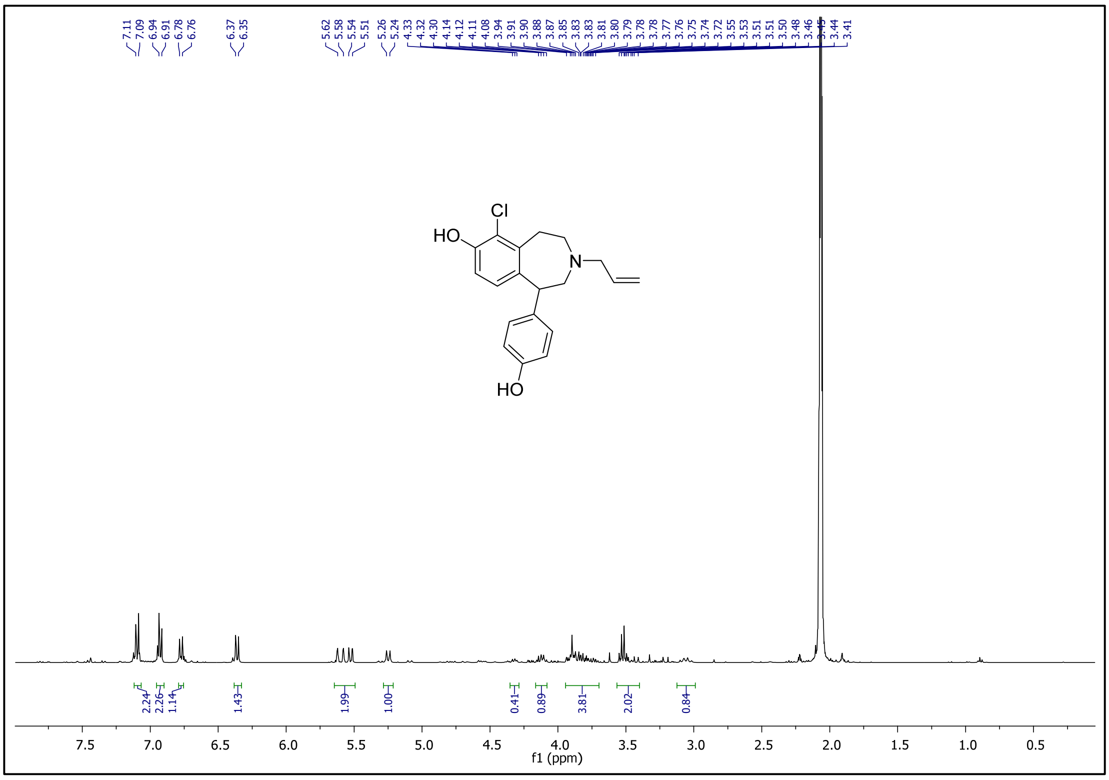
7.11
7.09
6.94
6.91
6.78
6.76
6.37
6.35
5.62
5.58
5.54
5.51
5.26
5.24
4.33
4.32
4.30
4.14
4.12
4.11
4.08
3.94
3.91
3.90
3.88
3.87
3.85
3.83
3.83
3.81
3.80
3.79
3.78
3.78
3.77
3.76
3.75
3.74
3.72
3.55
3.53
3.51
3.51
3.50
3.48
3.46
3.44
3.41
Cl
HO
N
HO
2.24
2.26
1.14
1.43
1.99
1.00
0.41
0.89
3.81
2.02
0.84
f1 (ppm)

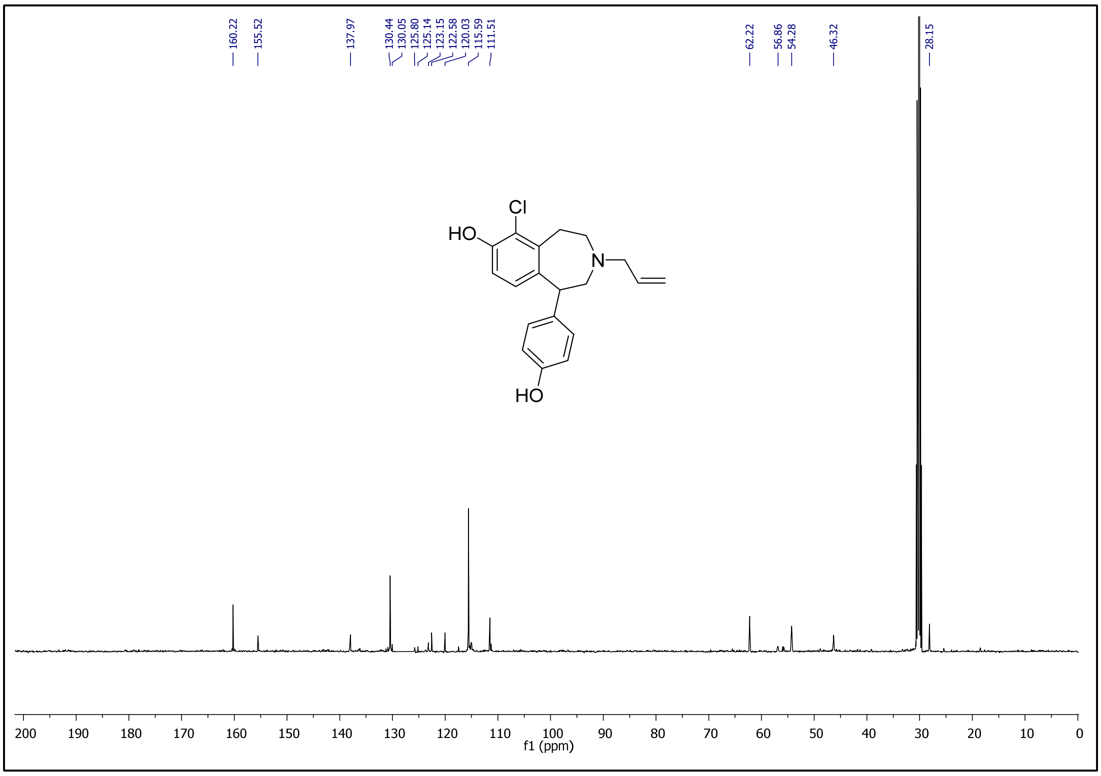
160.22
155.52
137.97
130.44
130.05
125.80
125.14
123.15
122.58
120.03
115.59
111.51
62.22
56.86
54.28
46.32
28.15
Cl
HO
N
HO
f1 (ppm)

15a

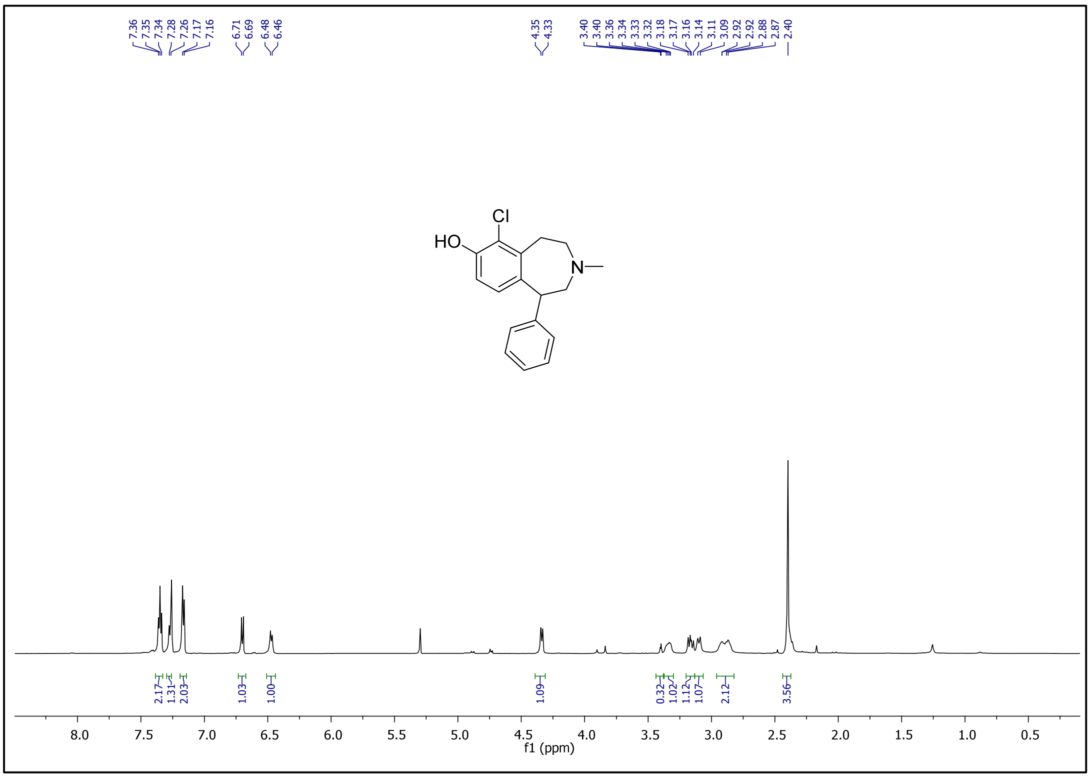

7.36
7.35
7.34
7.28
7.26
7.17
7.16
6.71
6.69
6.48
6.46
4.35
4.33
3.40
3.40
3.36
3.34
3.33
3.32
3.18
3.17
3.16
3.14
3.11
3.09
2.92
2.92
2.88
2.87
2.40
Cl
HO
N
2.17
1.31
2.03
1.03
1.00
1.09
0.32
1.02
1.12
1.07
2.12
3.56
8.0
7.5
7.0
6.5
6.0
5.5
5.0
4.5
4.0
3.5
3.0
2.5
2.0
1.5
1.0
0.5
f1 (ppm)

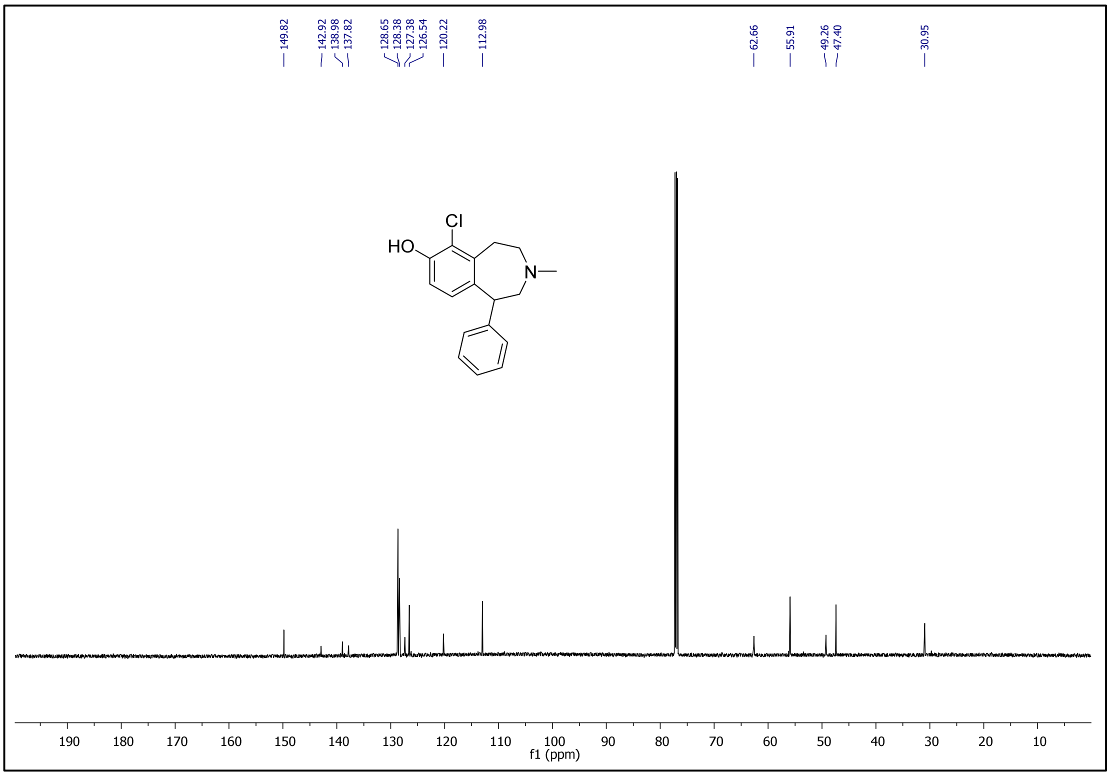

149.82
142.92
138.98
137.82
128.65
128.38
127.38
126.54
120.22
112.98
62.66
55.91
49.26
47.40
30.95
Cl
HO
N
190
180
170
160
150
140
130
120
110
100
90
80
70
60
50
40
30
20
10
f1 (ppm)

15b

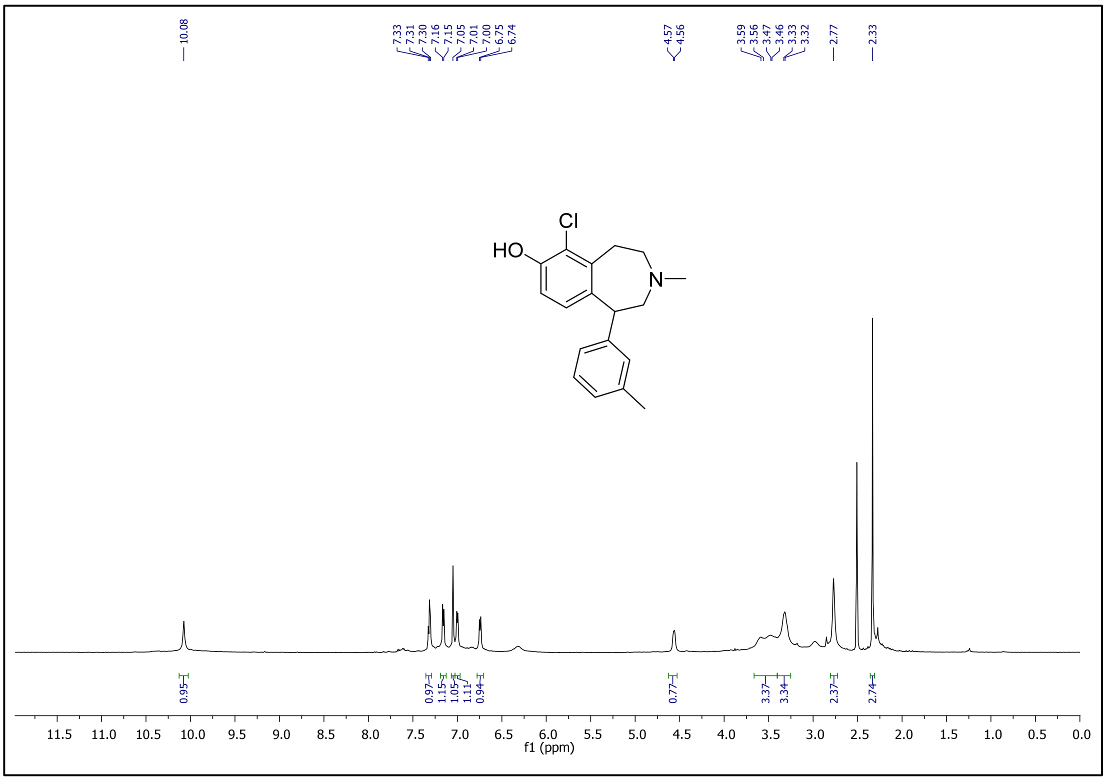
10.08
7.33 7.31 7.30 7.16 7.15 7.05 7.01 7.00 6.75 6.74
4.57 4.56
3.59 3.56 3.47 3.46 3.33 3.32
2.77
2.33
Cl
HO
N
0.95
0.97 1.15 1.05 1.11 0.94
0.77
3.37 3.34
2.37
2.74
11.5 11.0 10.5 10.0 9.5 9.0 8.5 8.0 7.5 7.0 6.5 6.0 5.5 5.0 4.5 4.0 3.5 3.0 2.5 2.0 1.5 1.0 0.5 0.0
f1 (ppm)

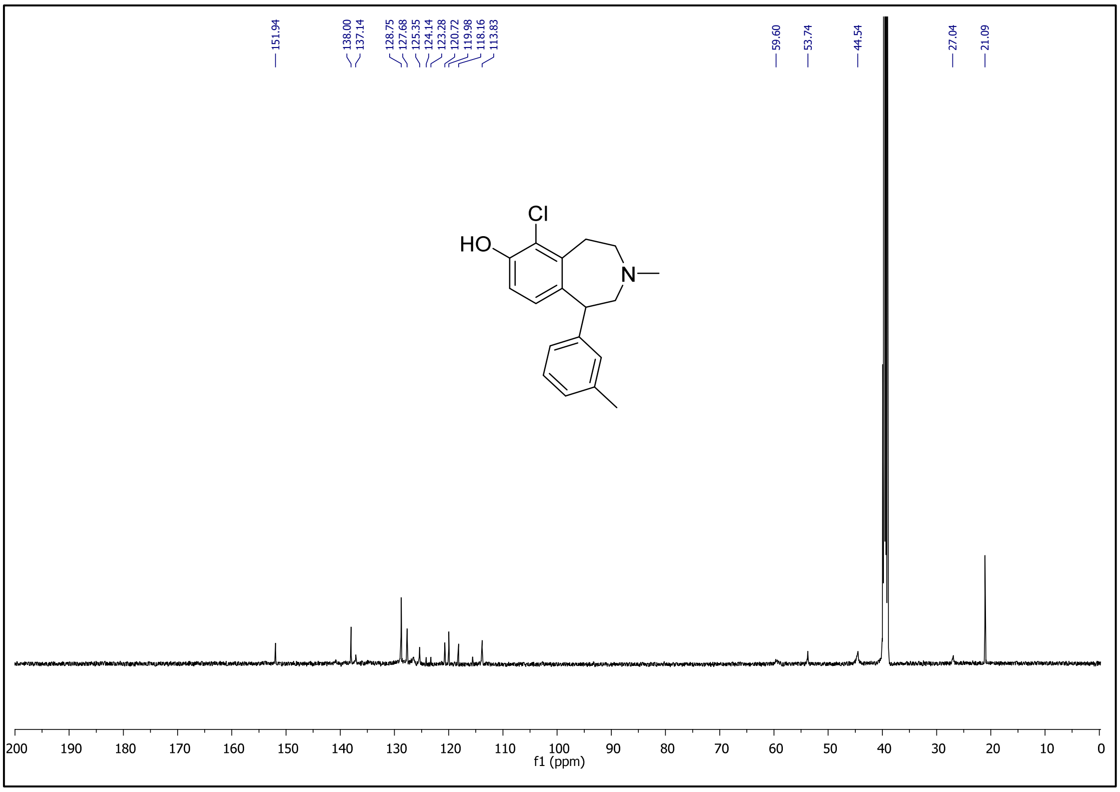
151.94
138.00 137.14
128.75 127.68 125.35 124.14 123.28 120.72 119.98 118.16 113.83
59.60
53.74
44.54
27.04
21.09
Cl
HO
N
200 190 180 170 160 150 140 130 120 110 100 90 80 70 60 50 40 30 20 10 0
f1 (ppm)

16a

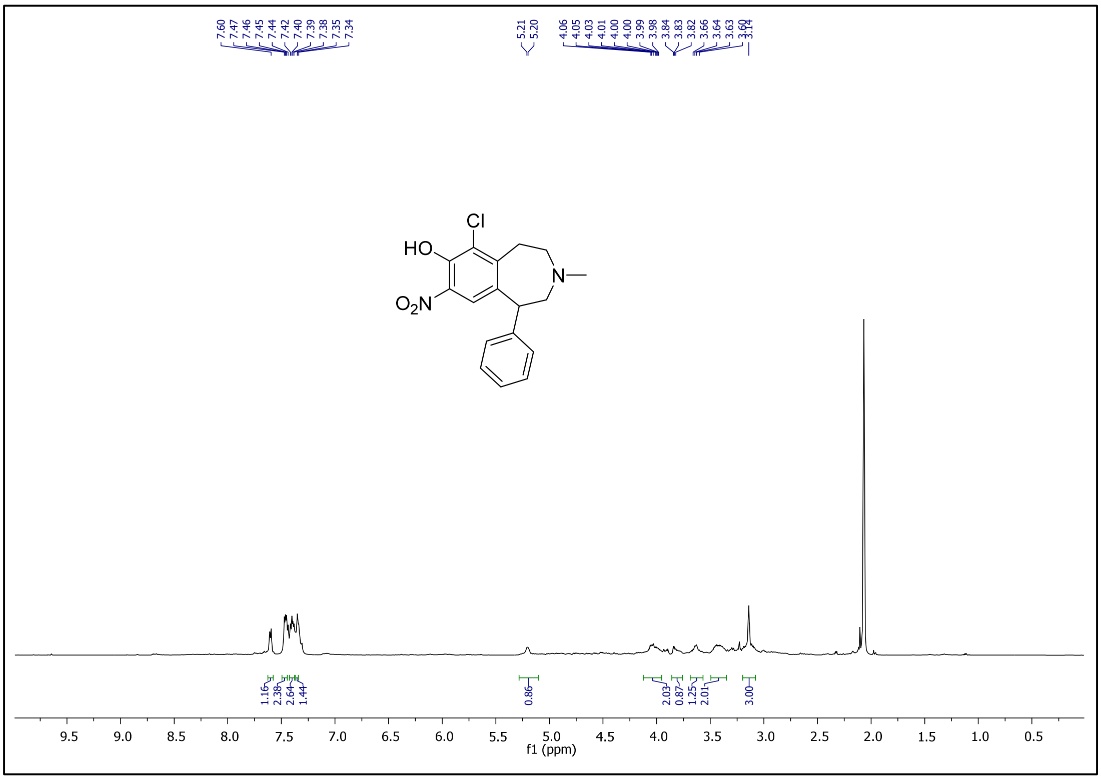

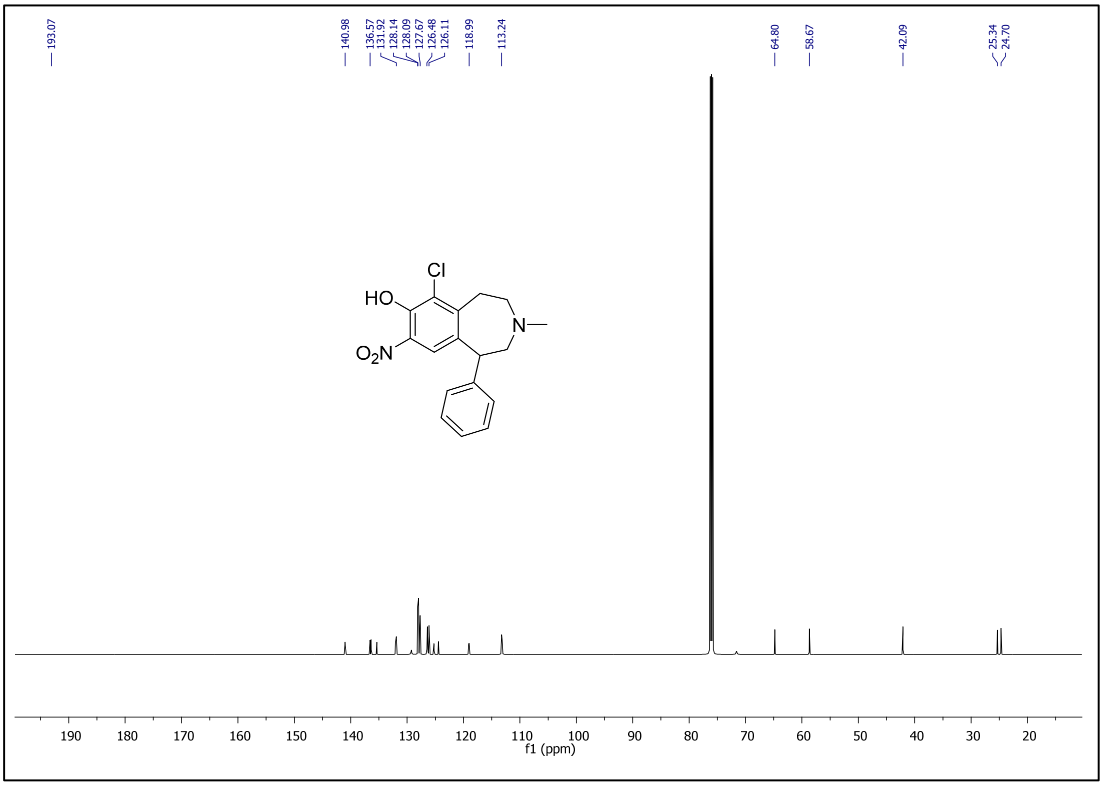

16b

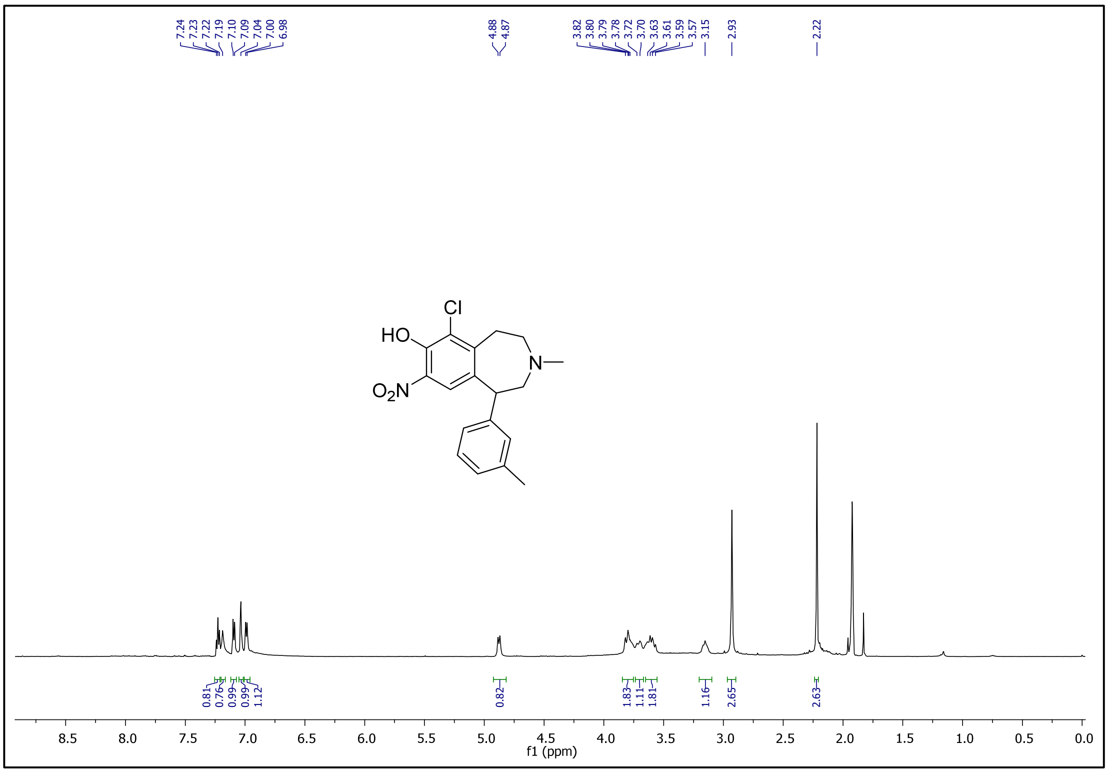
7.24
7.23
7.22
7.19
7.10
7.09
7.04
7.00
6.98
4.88
4.87
3.82
3.80
3.79
3.78
3.72
3.70
3.63
3.61
3.59
3.57
3.15
2.93
2.22
Cl
HO
N
O2N
0.81
0.76
0.99
0.99
1.12
0.82
1.83
1.11
1.81
1.16
2.65
2.63
8.5 8.0 7.5 7.0 6.5 6.0 5.5 5.0 4.5 4.0 3.5 3.0 2.5 2.0 1.5 1.0 0.5 0.0
f1 (ppm)

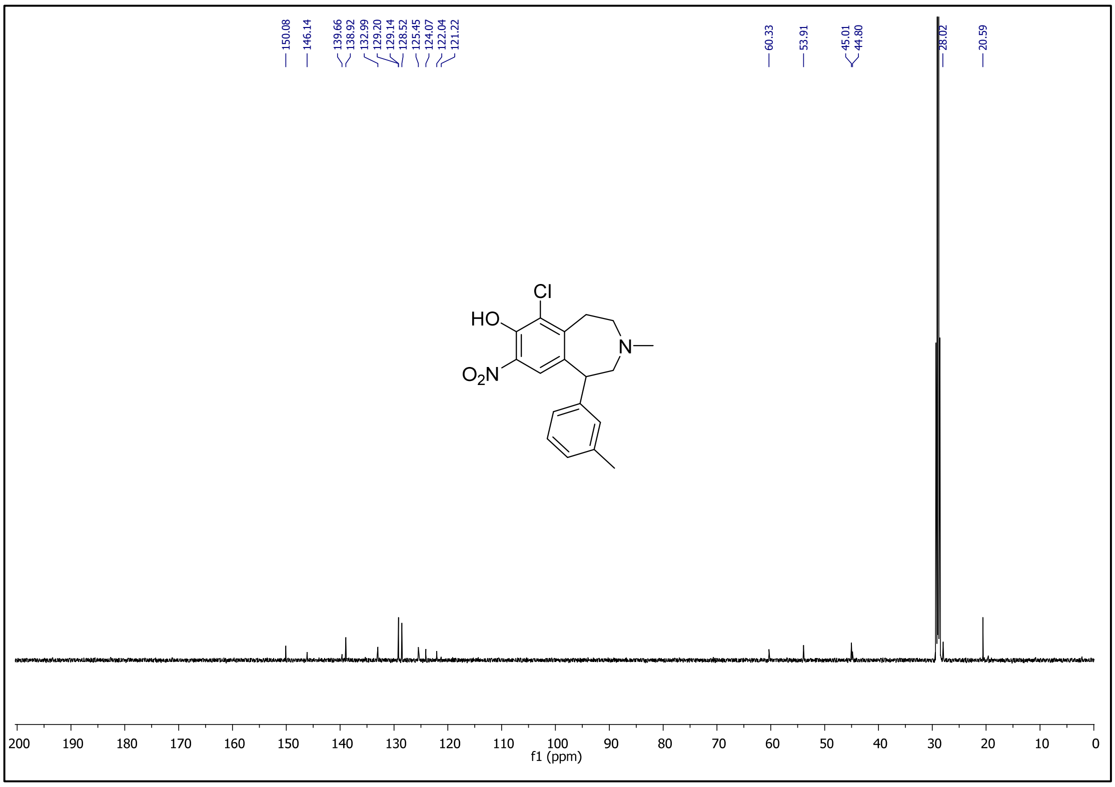
150.08
146.14
139.66
138.92
132.99
129.20
129.14
128.52
125.45
124.07
122.04
121.22
60.33
53.91
45.01
44.80
28.02
20.59
Cl
HO
N
O2N
200 190 180 170 160 150 140 130 120 110 100 90 80 70 60 50 40 30 20 10 0
f1 (ppm)

17a

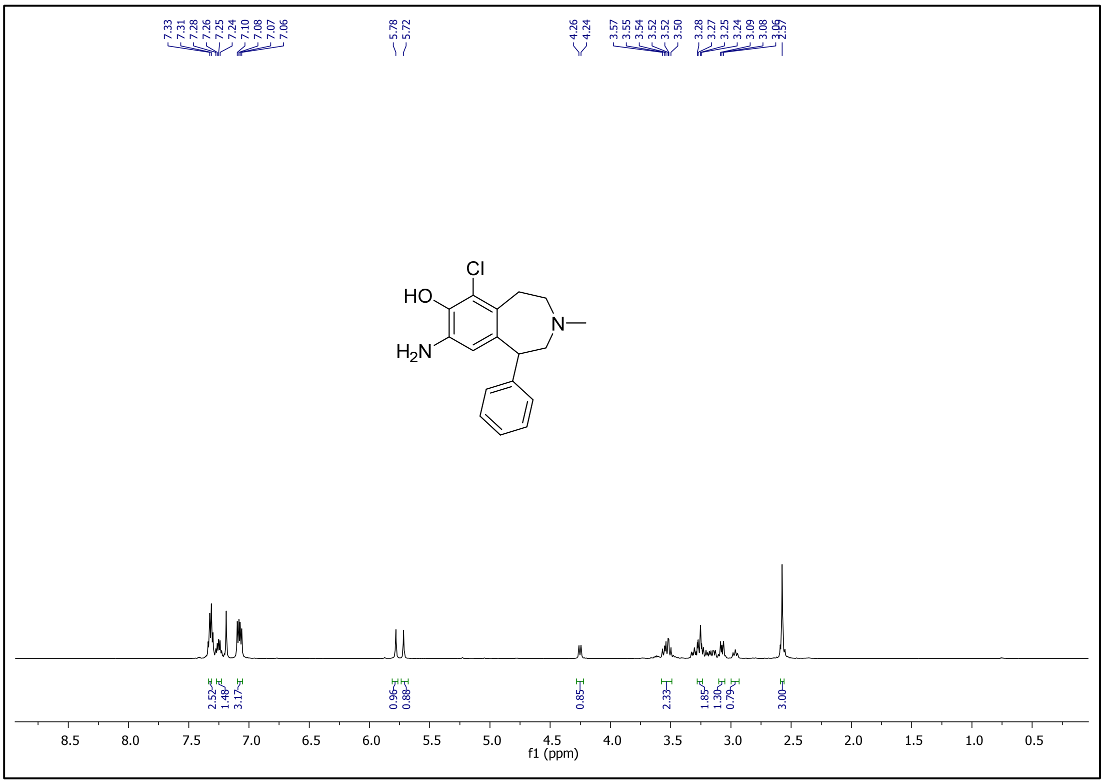
7.33
7.31
7.28
7.26
7.25
7.24
7.10
7.08
7.07
7.06
5.78
5.72
4.26
4.24
3.57
3.55
3.54
3.52
3.52
3.50
3.28
3.27
3.25
3.24
3.09
3.08
3.06
2.57
Cl
HO
N
H2N
2.52
1.40
3.17
0.96
0.88
0.85
2.33
1.85
1.30
0.79
3.00
8.5
8.0
7.5
7.0
6.5
6.0
5.5
5.0
4.5
4.0
3.5
3.0
2.5
2.0
1.5
1.0
0.5
f1 (ppm)

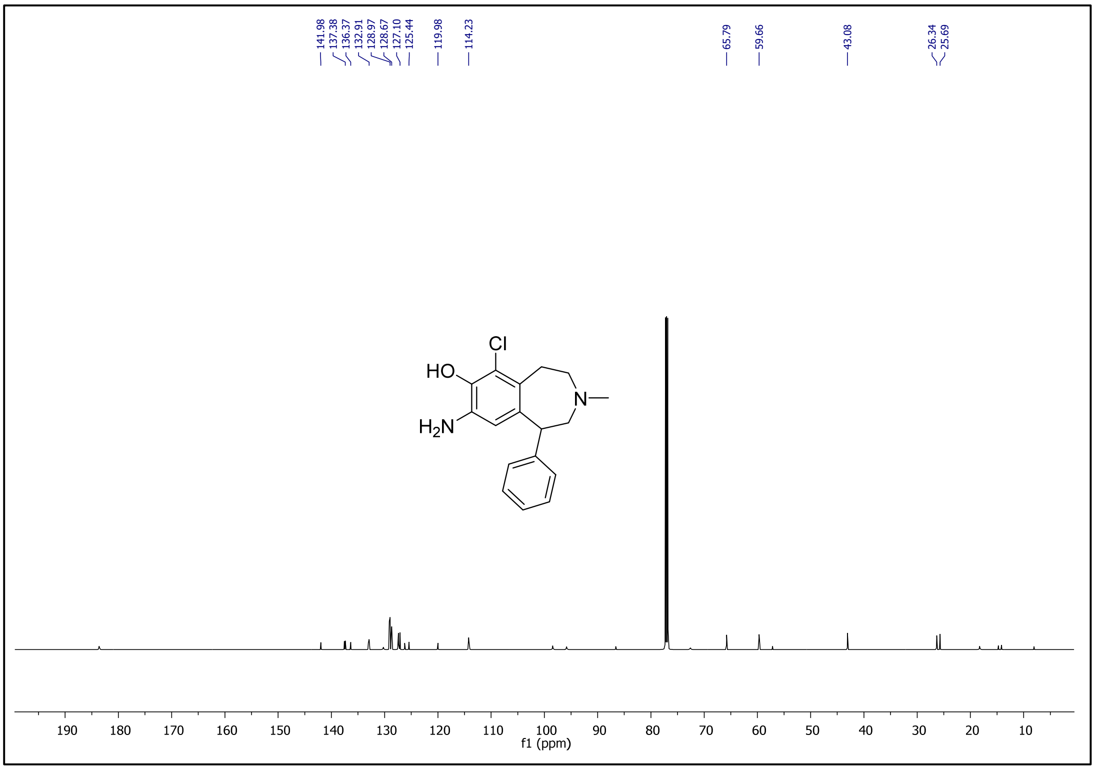
141.98
137.38
136.37
132.91
128.97
128.67
127.10
125.44
119.98
114.23
65.79
59.66
43.08
26.34
25.69
Cl
HO
N
H2N
190
180
170
160
150
140
130
120
110
100
90
80
70
60
50
40
30
20
10
f1 (ppm)

17b

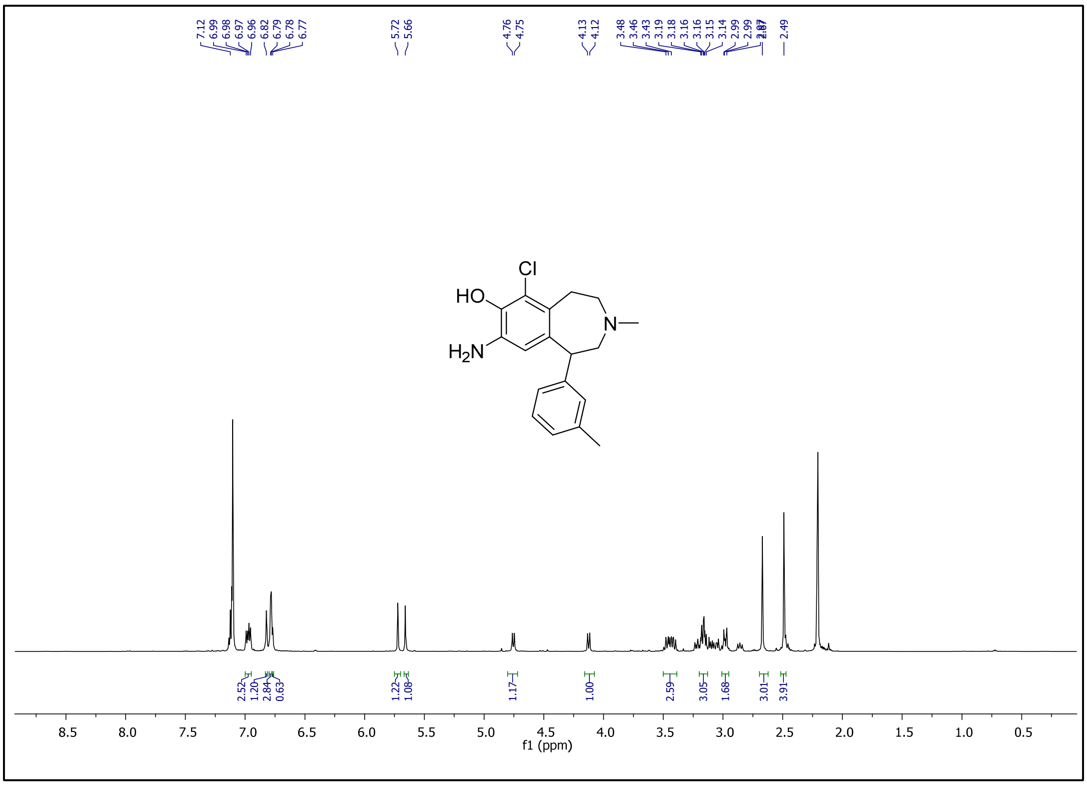

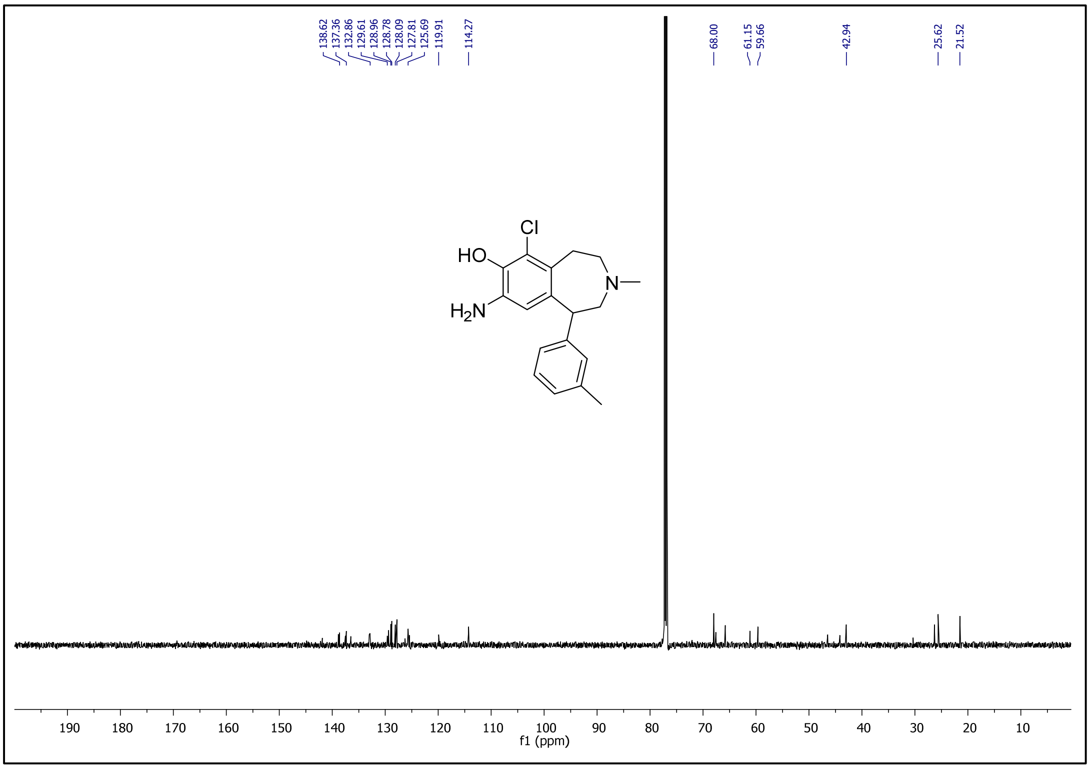

18a

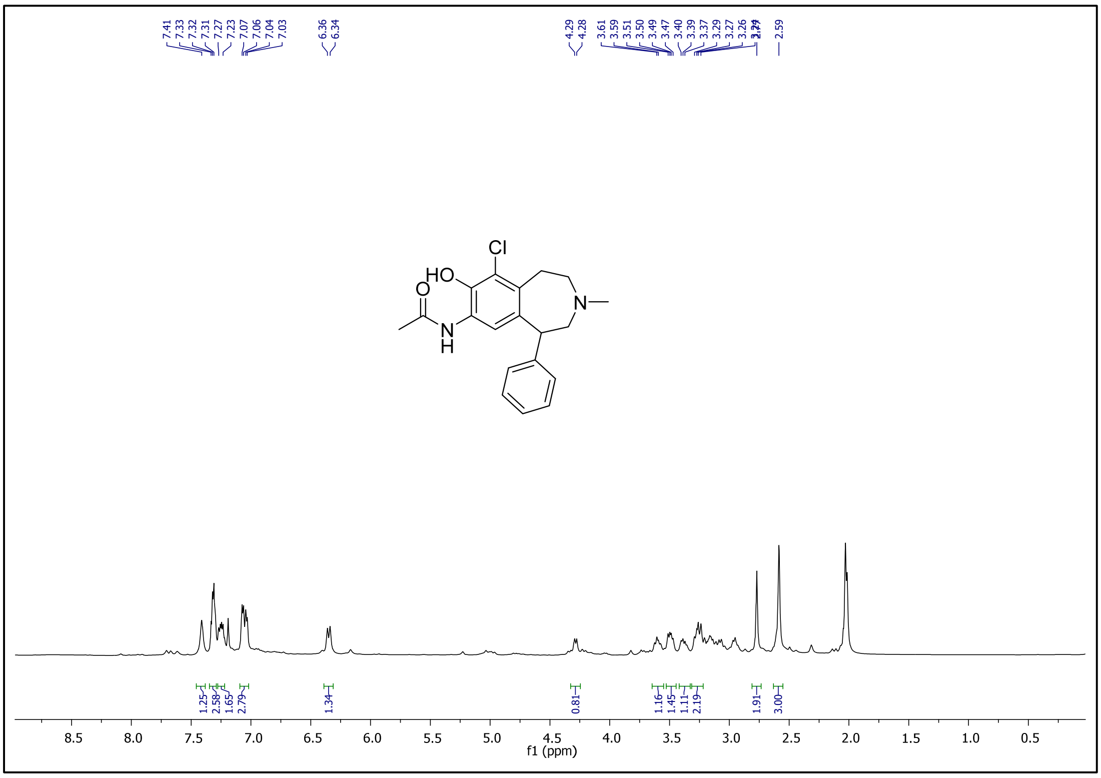
7.41
7.33
7.32
7.31
7.27
7.23
7.07
7.06
7.04
7.03
6.36
6.34
4.29
4.28
3.61
3.59
3.51
3.50
3.49
3.47
3.40
3.39
3.37
3.29
3.27
3.26
2.59
Cl
HO
O
N
N
H
1.25
2.58
1.65
2.79
1.34
0.81
1.16
1.45
1.11
2.19
1.91
3.00
8.5
8.0
7.5
7.0
6.5
6.0
5.5
5.0
4.5
4.0
3.5
3.0
2.5
2.0
1.5
1.0
0.5
f1 (ppm)

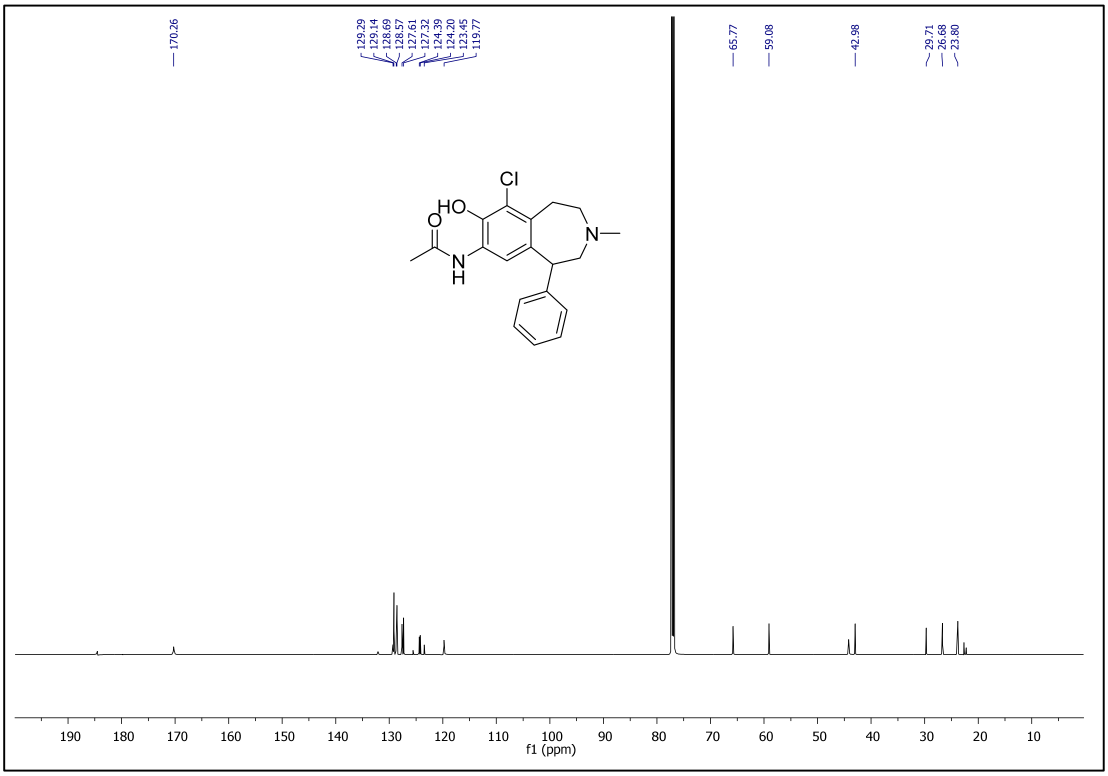
170.26
129.29
129.14
128.69
128.57
127.61
127.32
124.39
124.20
123.45
119.77
65.77
59.08
42.98
29.71
26.68
23.80
Cl
HO
O
N
N
H
190
180
170
160
150
140
130
120
110
100
90
80
70
60
50
40
30
20
10
f1 (ppm)

18b

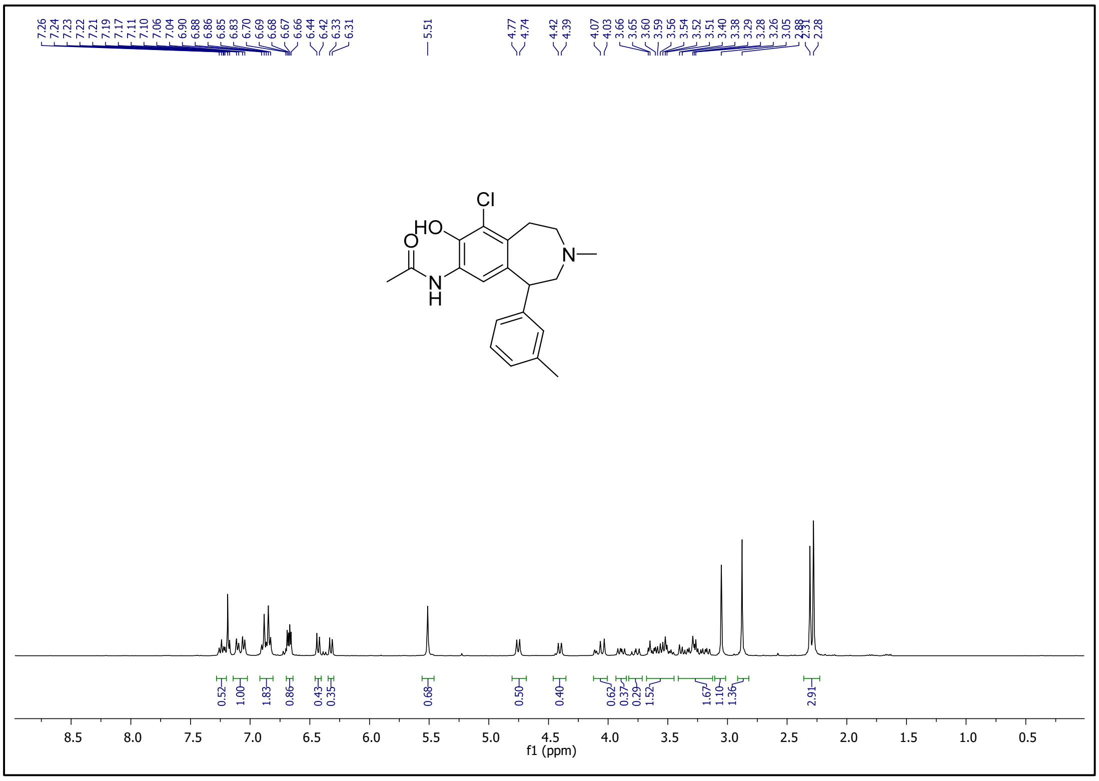
Cl
HO
O
N
N
H
8.5
8.0
7.5
7.0
6.5
6.0
5.5
5.0
4.5
4.0
3.5
3.0
2.5
2.0
1.5
1.0
0.5
f1 (ppm)

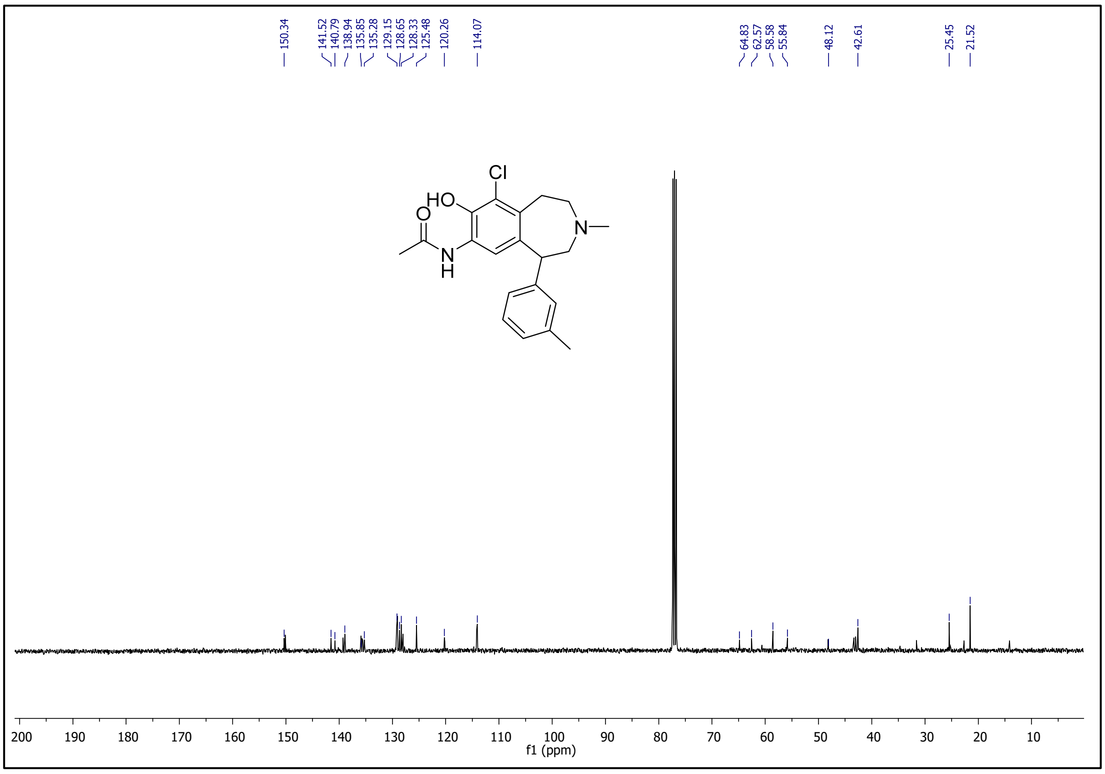
Cl
HO
O
N
N
H
200
190
180
170
160
150
140
130
120
110
100
90
80
70
60
50
40
30
20
10
f1 (ppm)

19a

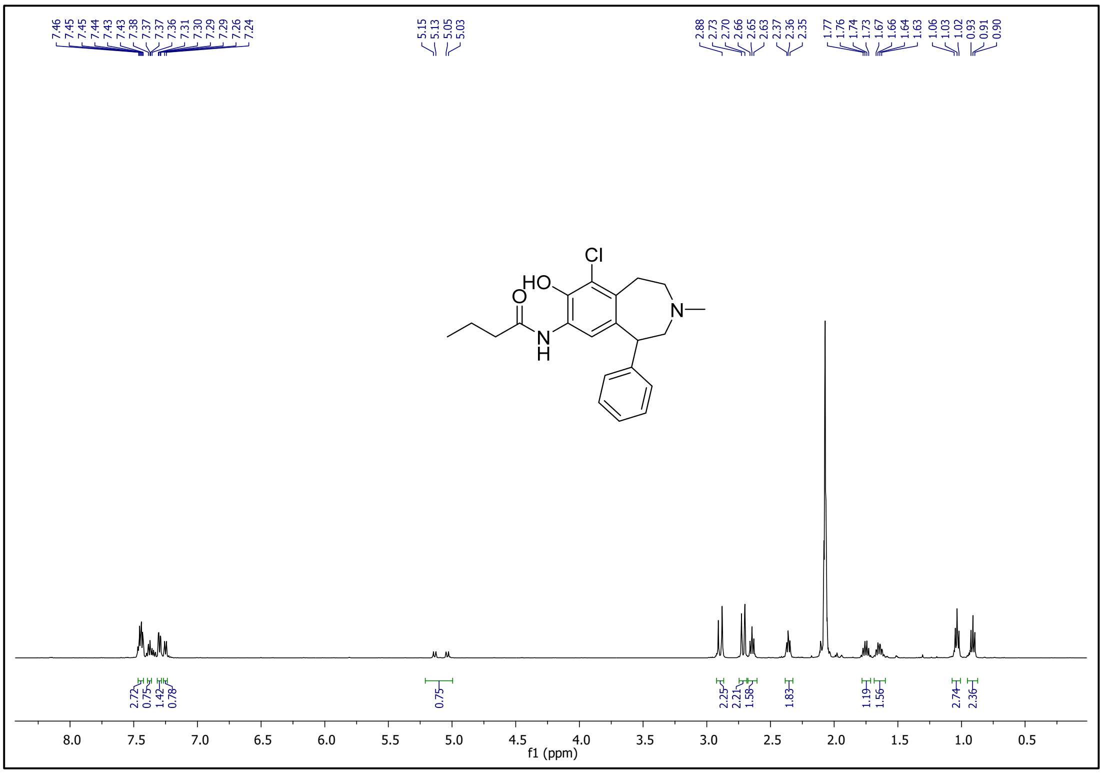

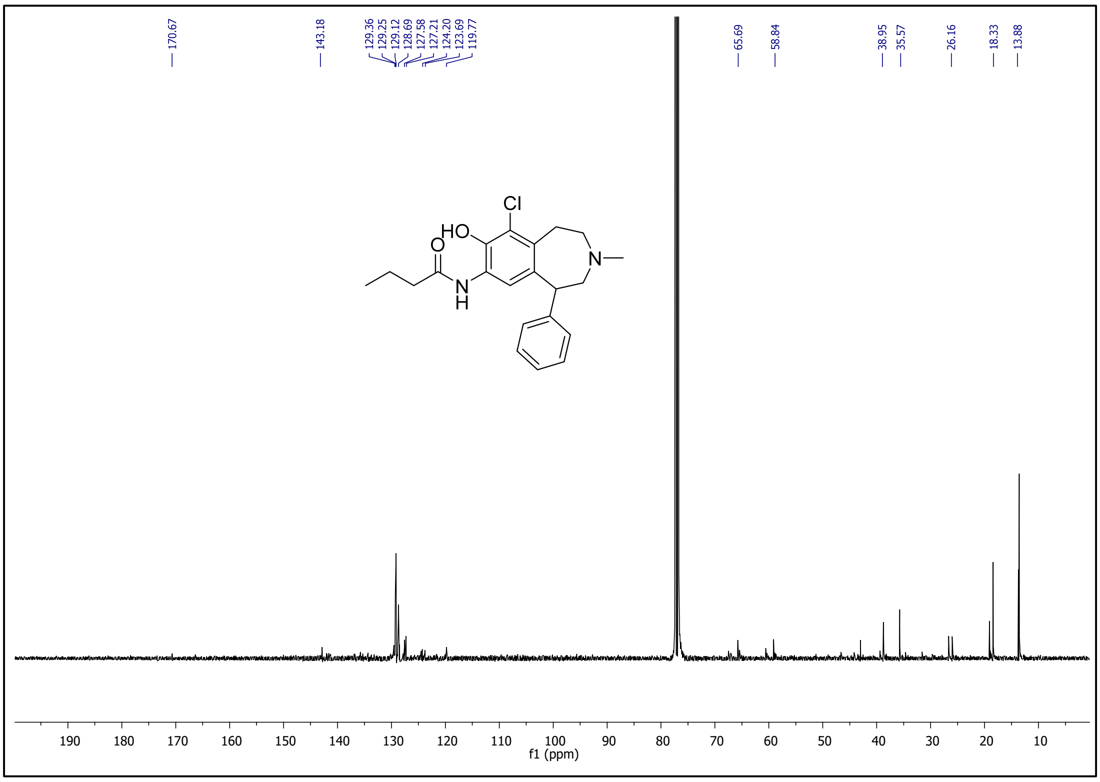

19b

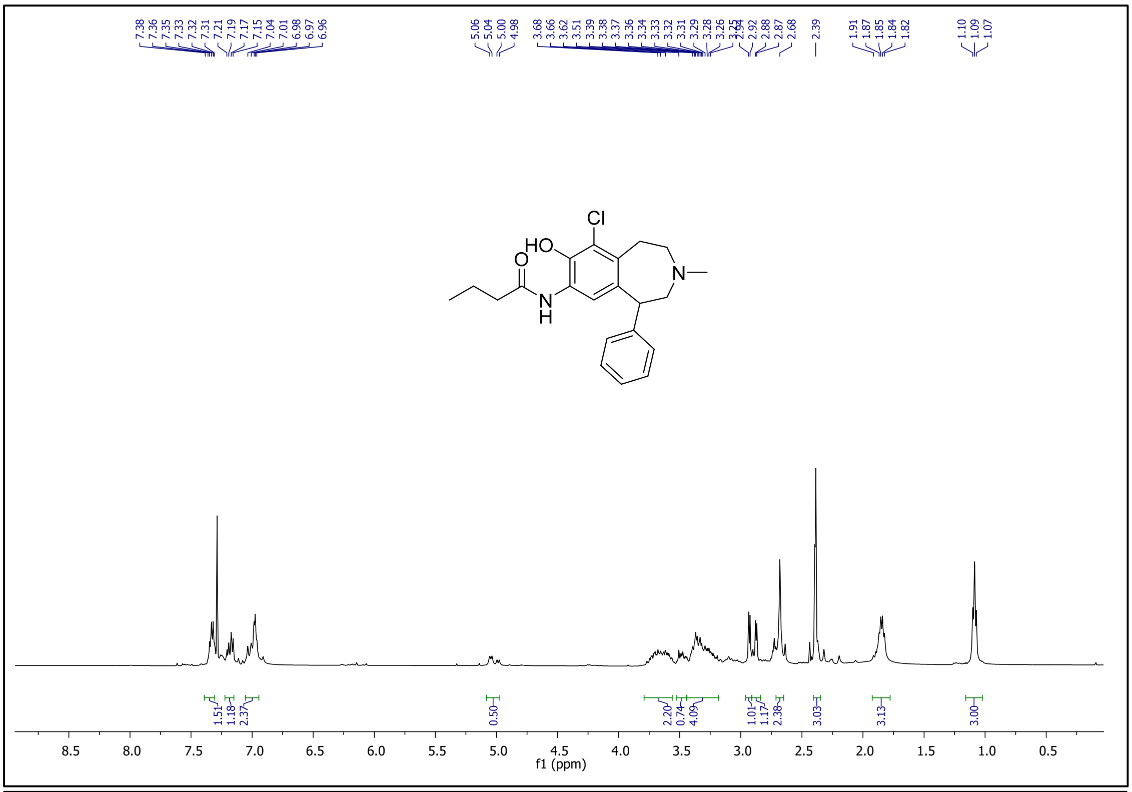

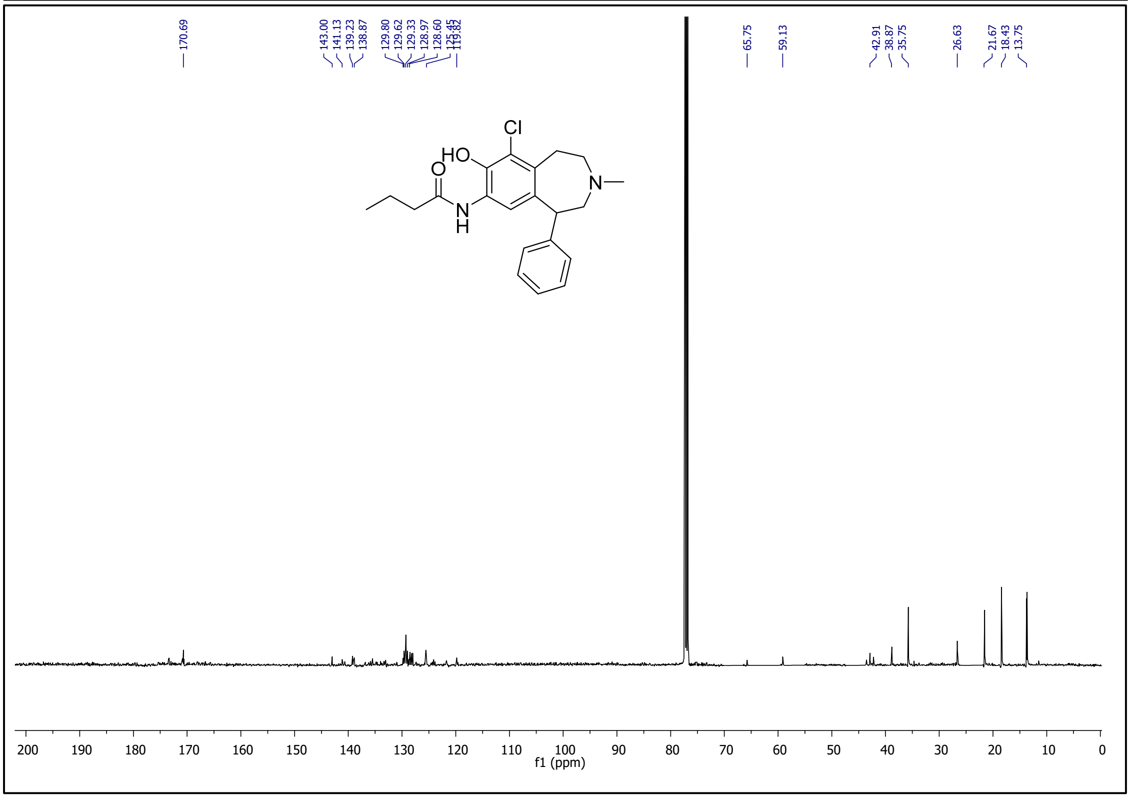

20a

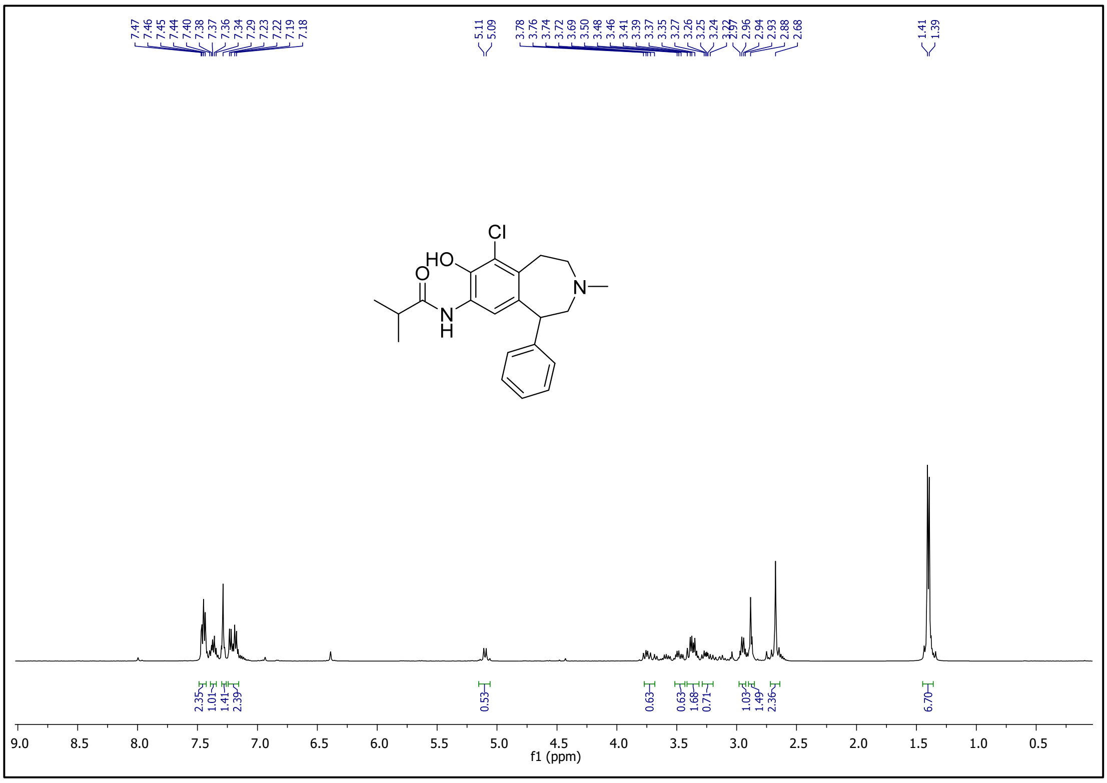

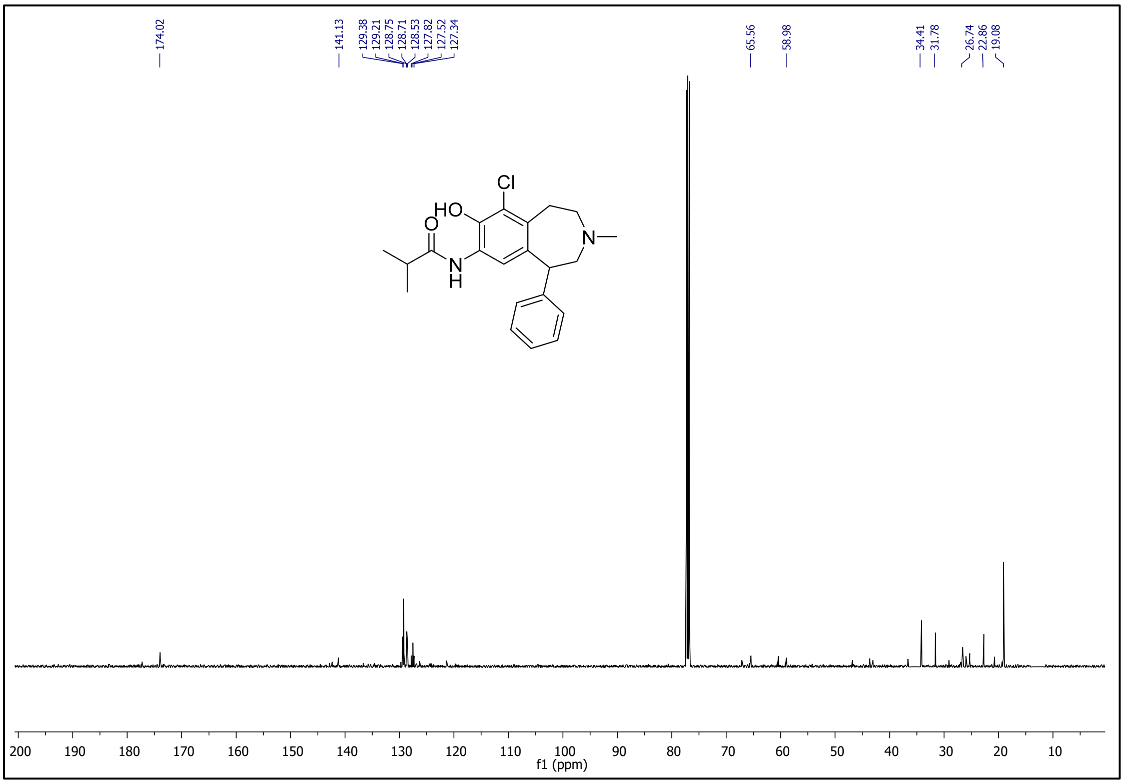

21a

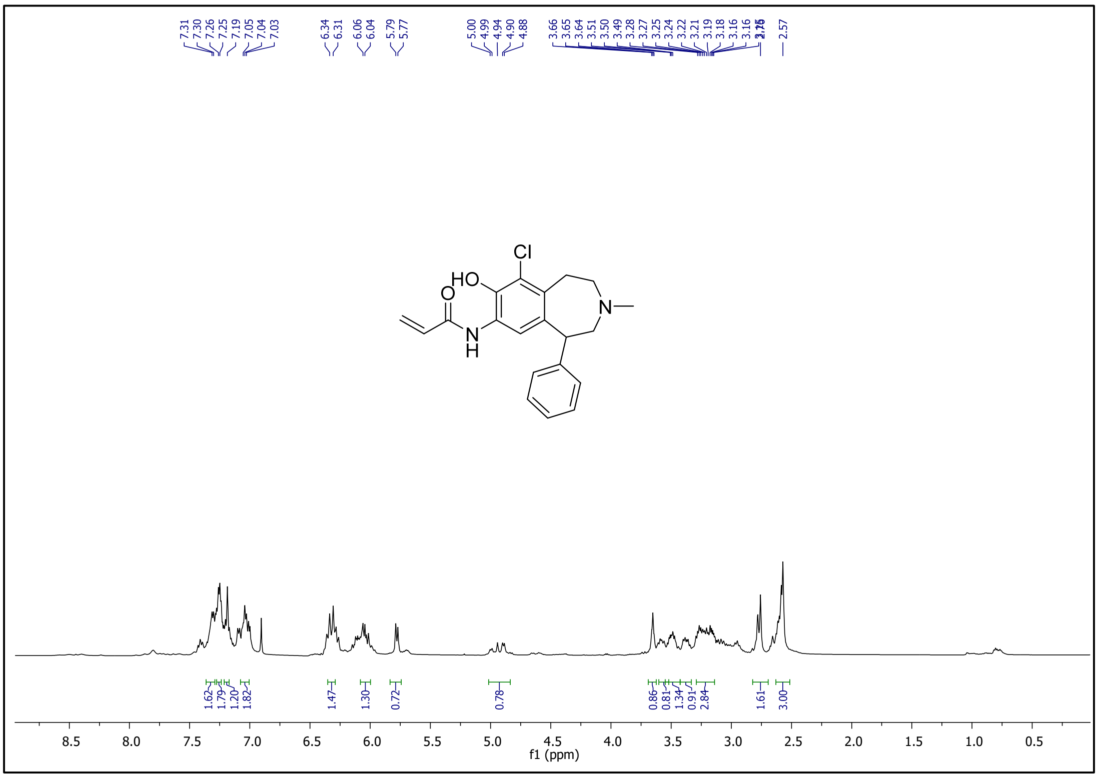
7.31
7.30
7.26
7.25
7.19
7.05
7.04
7.03
6.34
6.31
6.06
6.04
5.79
5.77
5.00
4.99
4.94
4.90
4.88
3.66
3.65
3.64
3.51
3.50
3.49
3.28
3.27
3.25
3.24
3.22
3.21
3.19
3.18
3.16
3.16
2.57
Cl
HO
O
N
N
H
1.62
1.79
1.20
1.82
1.47
1.30
0.72
0.78
0.86
0.81
1.34
0.91
2.84
1.61
3.00
8.5
8.0
7.5
7.0
6.5
6.0
5.5
5.0
4.5
4.0
3.5
3.0
2.5
2.0
1.5
1.0
0.5
f1 (ppm)

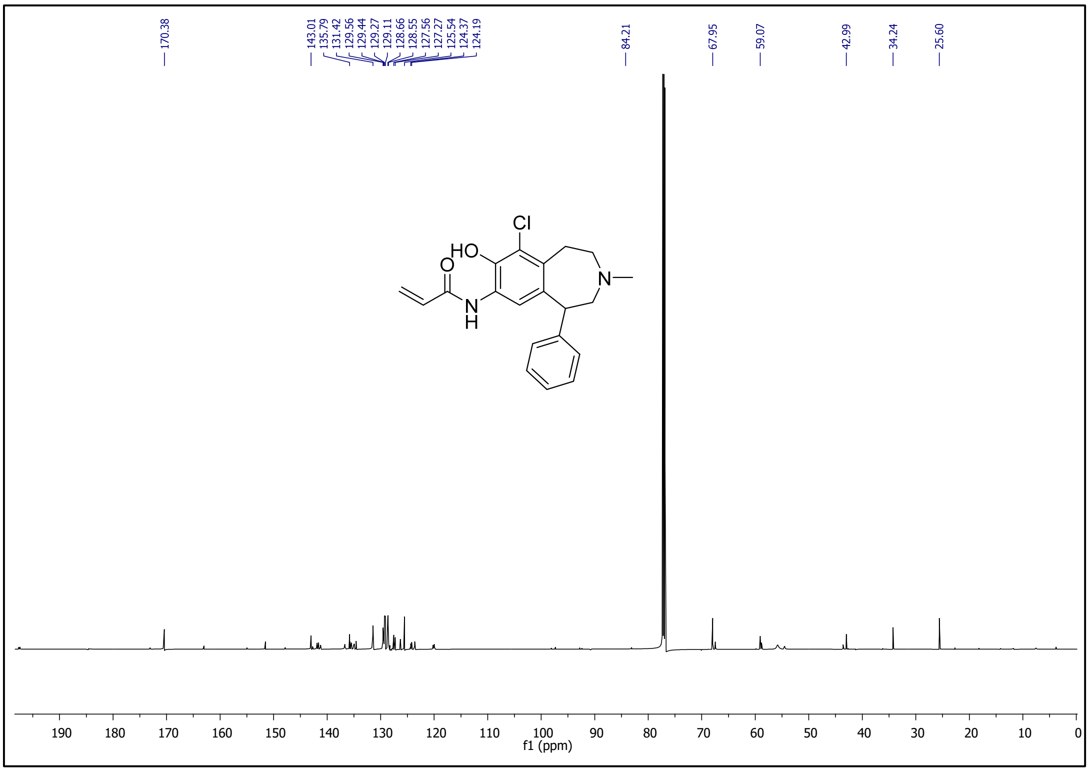
170.38
143.01
135.79
131.42
129.56
129.44
129.27
129.11
128.66
128.55
127.56
127.27
125.54
124.37
124.19
84.21
67.95
59.07
42.99
34.24
25.60
Cl
HO
O
N
N
H
190
180
170
160
150
140
130
120
110
100
90
80
70
60
50
40
30
20
10
0
f1 (ppm)

22

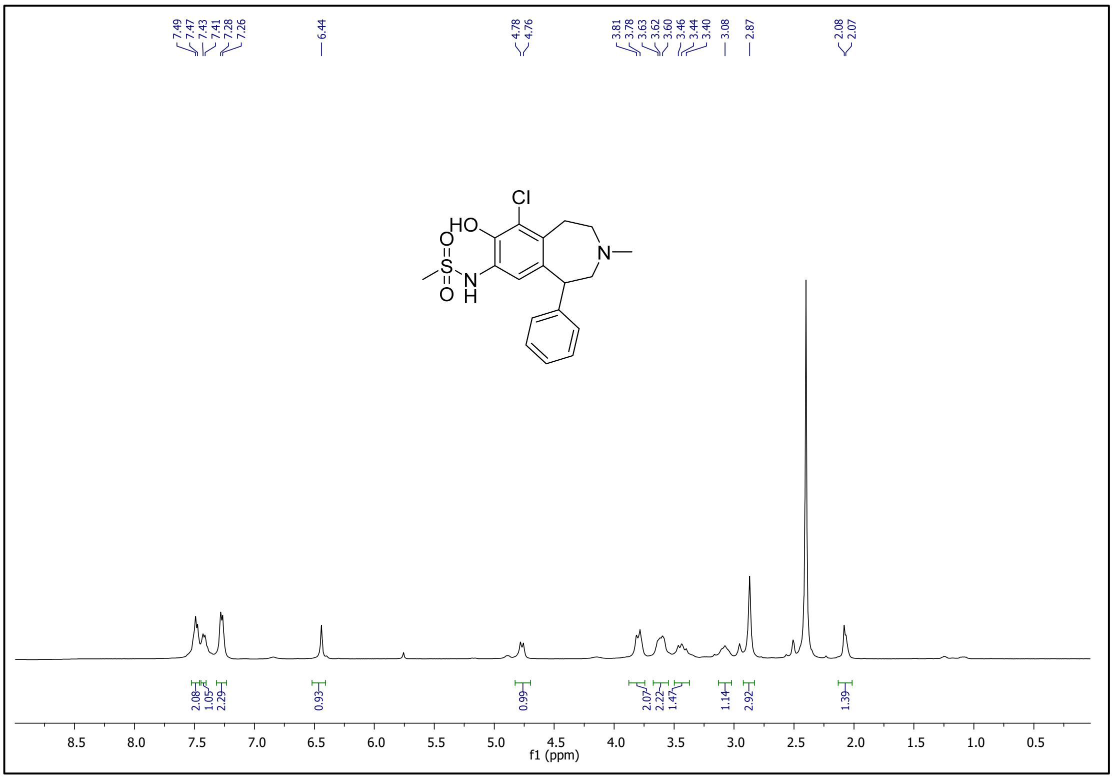
7.49
7.47
7.43
7.41
7.28
7.26
6.44
4.78
4.76
3.81
3.78
3.63
3.62
3.60
3.46
3.44
3.40
3.08
2.87
2.08
2.07
Cl
HO
O
S
O
N
H
N
2.08
1.05
2.29
0.93
0.99
2.07
2.22
1.47
1.14
2.92
1.39
8.5
8.0
7.5
7.0
6.5
6.0
5.5
5.0
4.5
4.0
3.5
3.0
2.5
2.0
1.5
1.0
0.5
f1 (ppm)

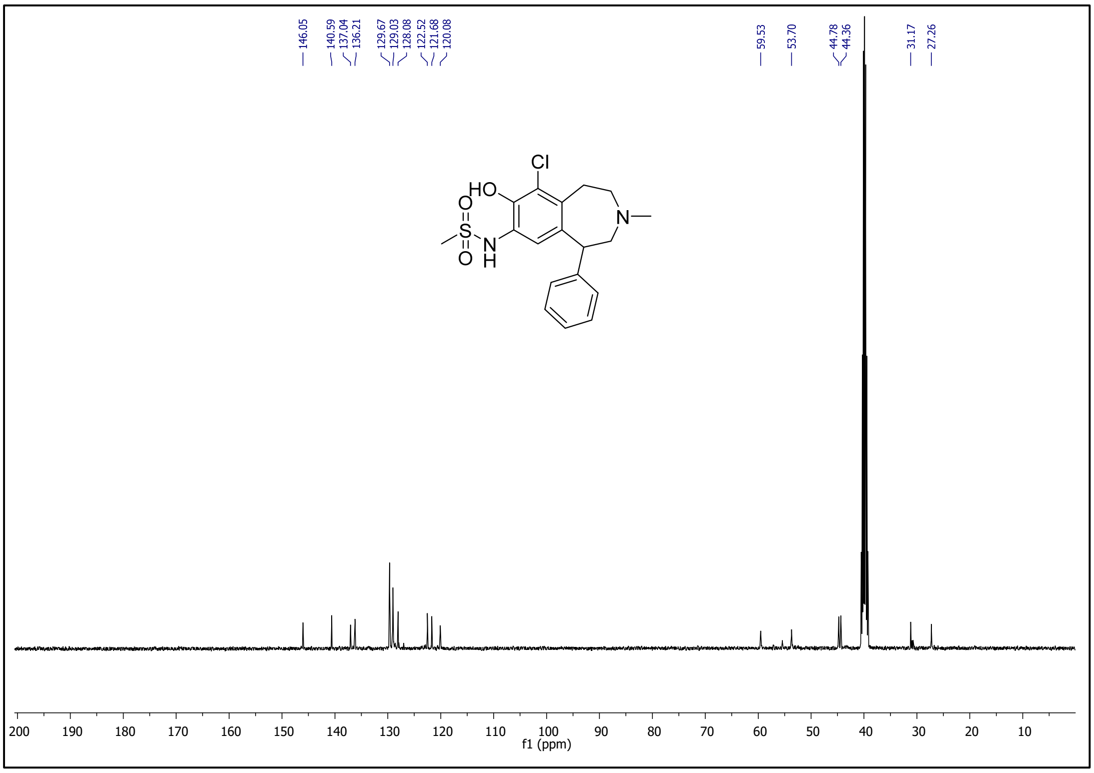
146.05
140.59
137.04
136.21
129.67
129.03
128.08
122.52
121.68
120.08
59.53
53.70
44.78
44.36
31.17
27.26
Cl
HO
O
S
O
N
H
N
200
190
180
170
160
150
140
130
120
110
100
90
80
70
60
50
40
30
20
10
f1 (ppm)

23a

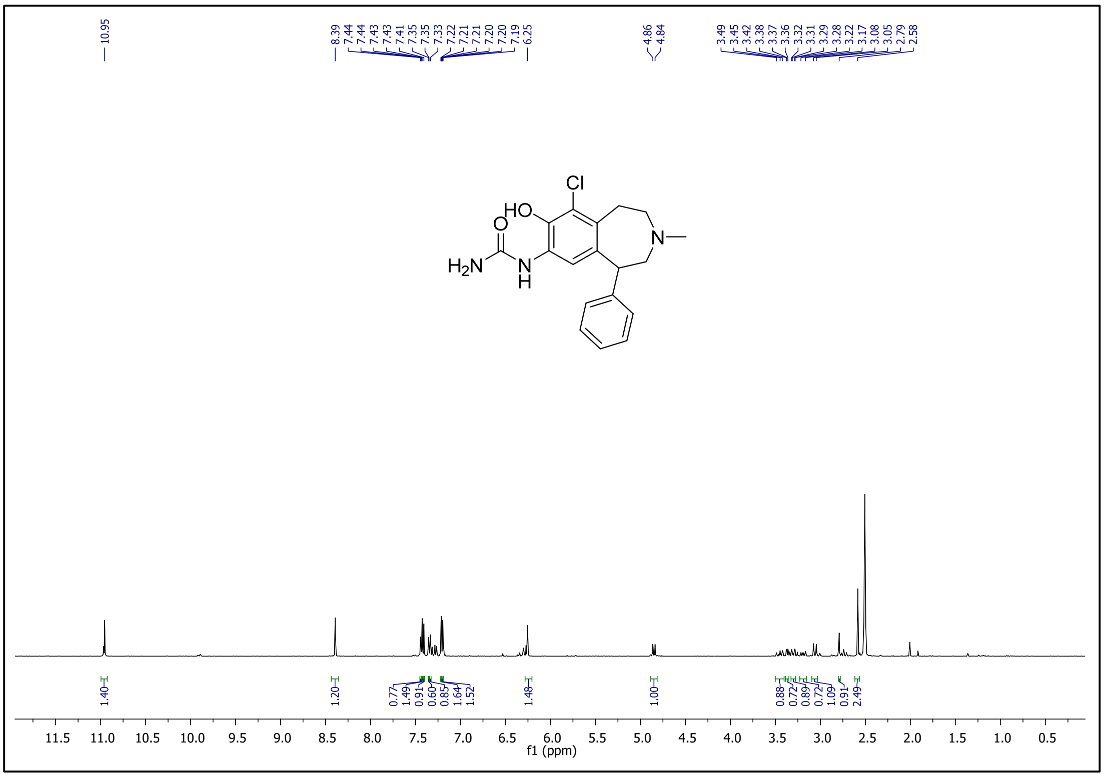
10.95
8.39 7.44 7.44 7.43 7.43 7.41 7.35 7.35 7.33 7.22 7.21 7.21 7.20 7.20 7.19 6.25
4.86 4.84
3.49 3.45 3.42 3.38 3.37 3.36 3.32 3.31 3.29 3.28 3.22 3.17 3.08 3.05 2.79 2.58
Cl
HO
O
H_2N
N
H
N
1.40
1.20
0.77 1.49 0.91 0.60 0.85 1.64 1.52
1.48
1.00
0.88 0.72 0.89 0.72 1.09 0.91 2.49
11.5 11.0 10.5 10.0 9.5 9.0 8.5 8.0 7.5 7.0 6.5 6.0 5.5 5.0 4.5 4.0 3.5 3.0 2.5 2.0 1.5 1.0 0.5
f1 (ppm)

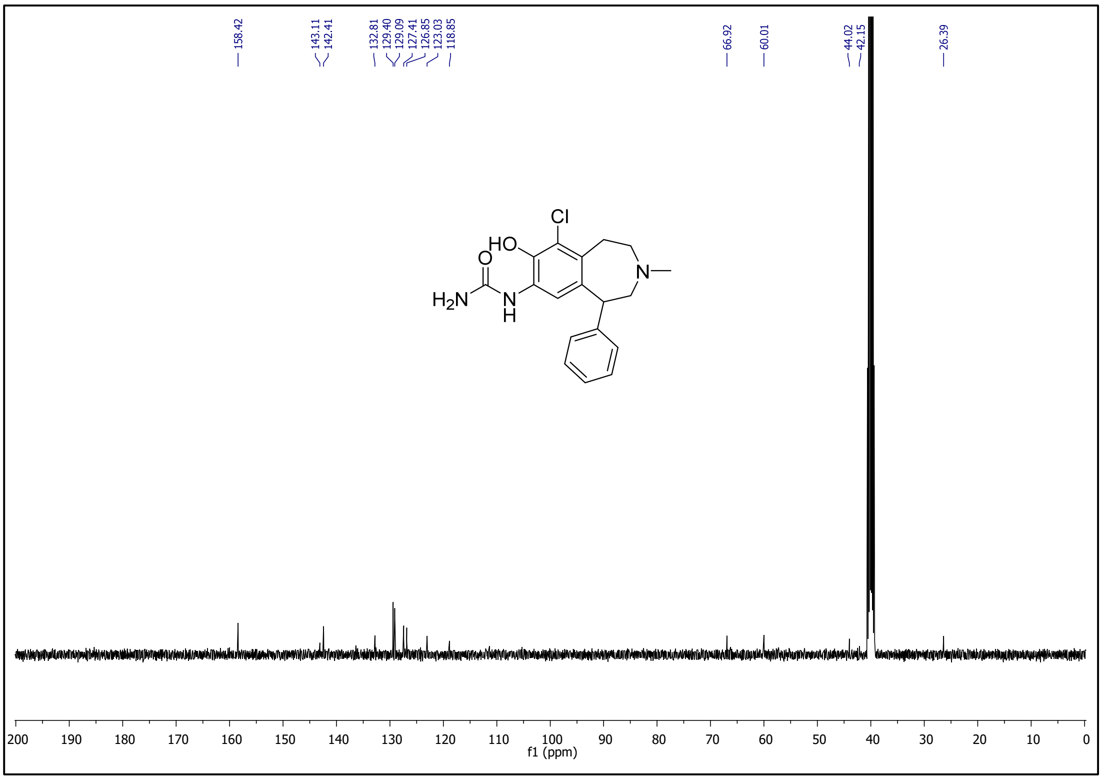
158.42
143.11 142.41
132.81 129.40 129.09 127.41 126.85 123.03 118.85
66.92
60.01
44.02 42.15
26.39
Cl
HO
O
H_2N
N
H
N
200 190 180 170 160 150 140 130 120 110 100 90 80 70 60 50 40 30 20 10 0
f1 (ppm)

23b

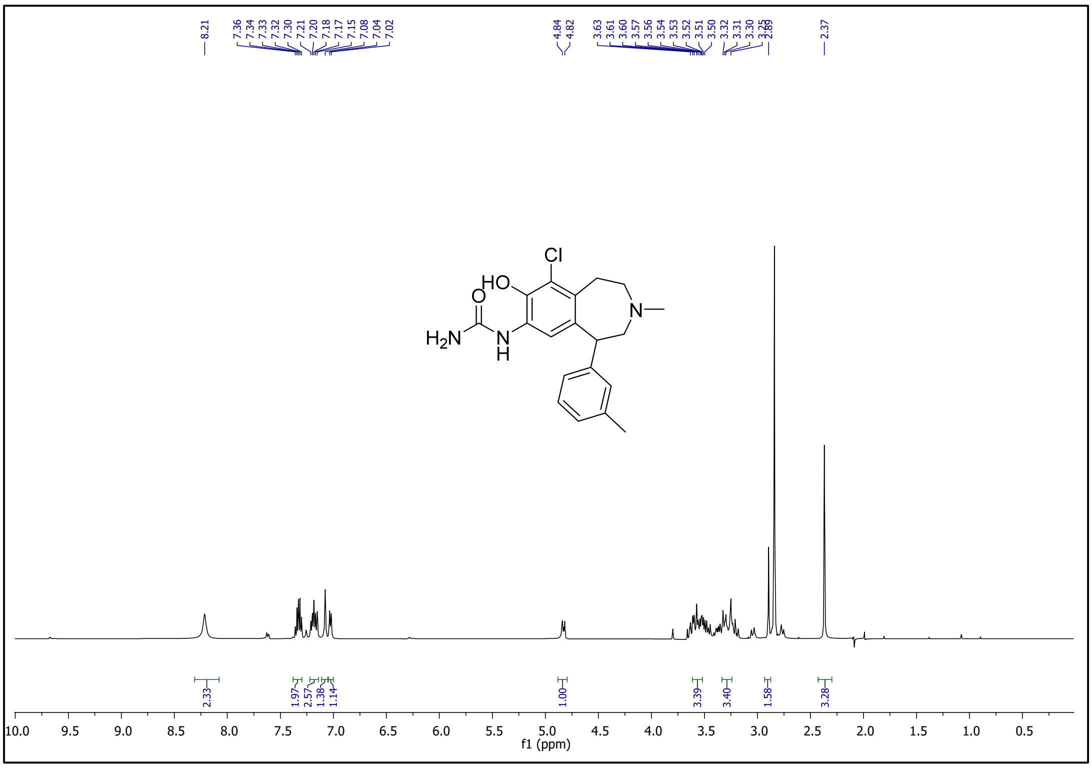
8.21
7.36
7.34
7.33
7.32
7.30
7.21
7.20
7.18
7.17
7.15
7.08
7.04
7.02
4.84
4.82
3.63
3.61
3.60
3.57
3.56
3.54
3.53
3.52
3.51
3.50
3.32
3.31
3.30
3.25
2.89
2.37
2.33
1.97
2.57
1.38
1.14
1.00
3.39
3.40
1.58
3.28
f1 (ppm)

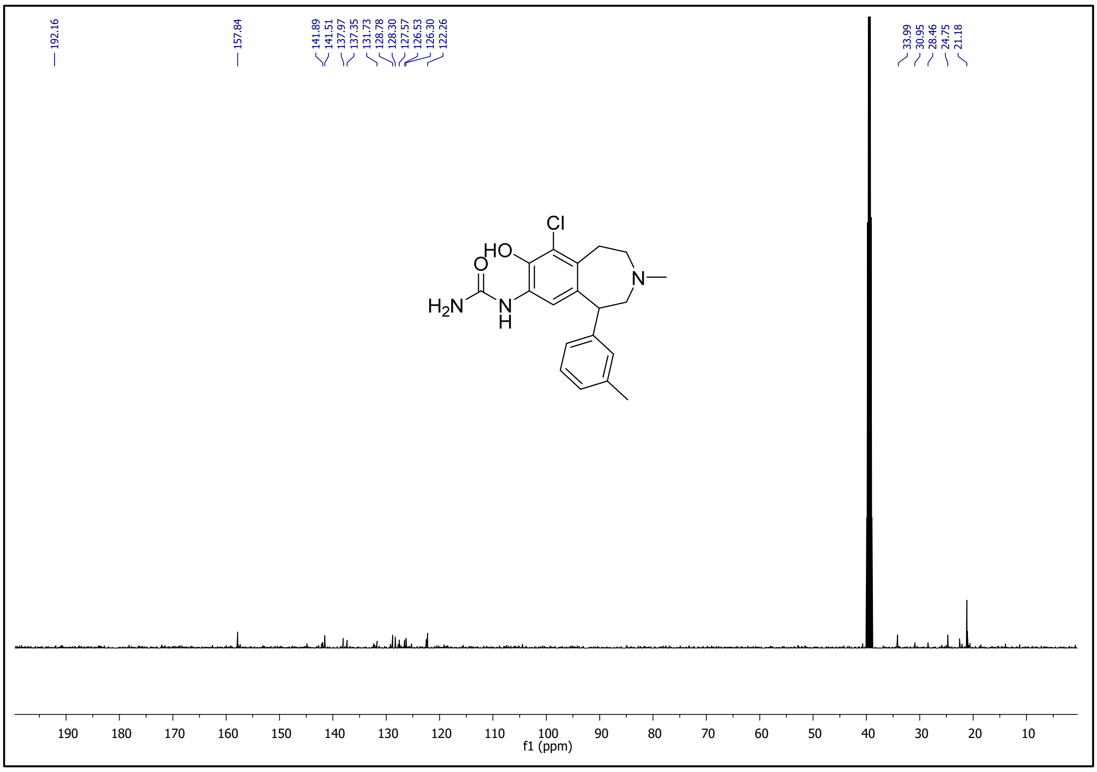
192.16
157.84
141.89
141.51
137.97
137.35
131.73
128.78
128.30
127.57
126.53
126.30
122.26
33.99
30.95
28.46
24.75
21.18
f1 (ppm)

24a

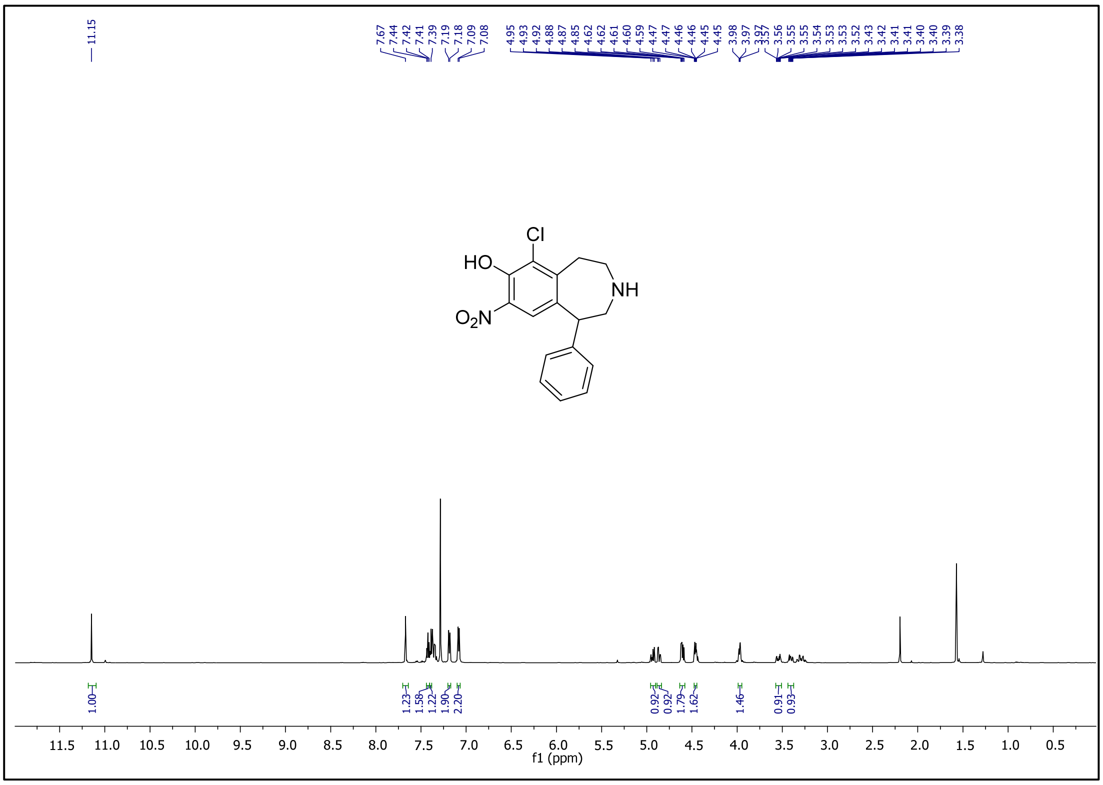
11.15
7.67 7.44 7.42 7.41 7.39 7.19 7.18 7.09 7.08
4.95 4.93 4.92 4.88 4.87 4.85 4.62 4.62 4.61 4.60 4.59 4.47 4.47 4.46 4.46 4.45 4.45
3.98 3.97 3.57 3.56 3.55 3.55 3.54 3.53 3.53 3.52 3.43 3.42 3.41 3.40 3.39 3.38
Cl
HO
NH
O2N
1.00 1.23 1.58 1.22 1.90 2.20 0.92 0.92 1.79 1.62 1.46 0.91 0.93
11.5 11.0 10.5 10.0 9.5 9.0 8.5 8.0 7.5 7.0 6.5 6.0 5.5 5.0 4.5 4.0 3.5 3.0 2.5 2.0 1.5 1.0 0.5
f1 (ppm)

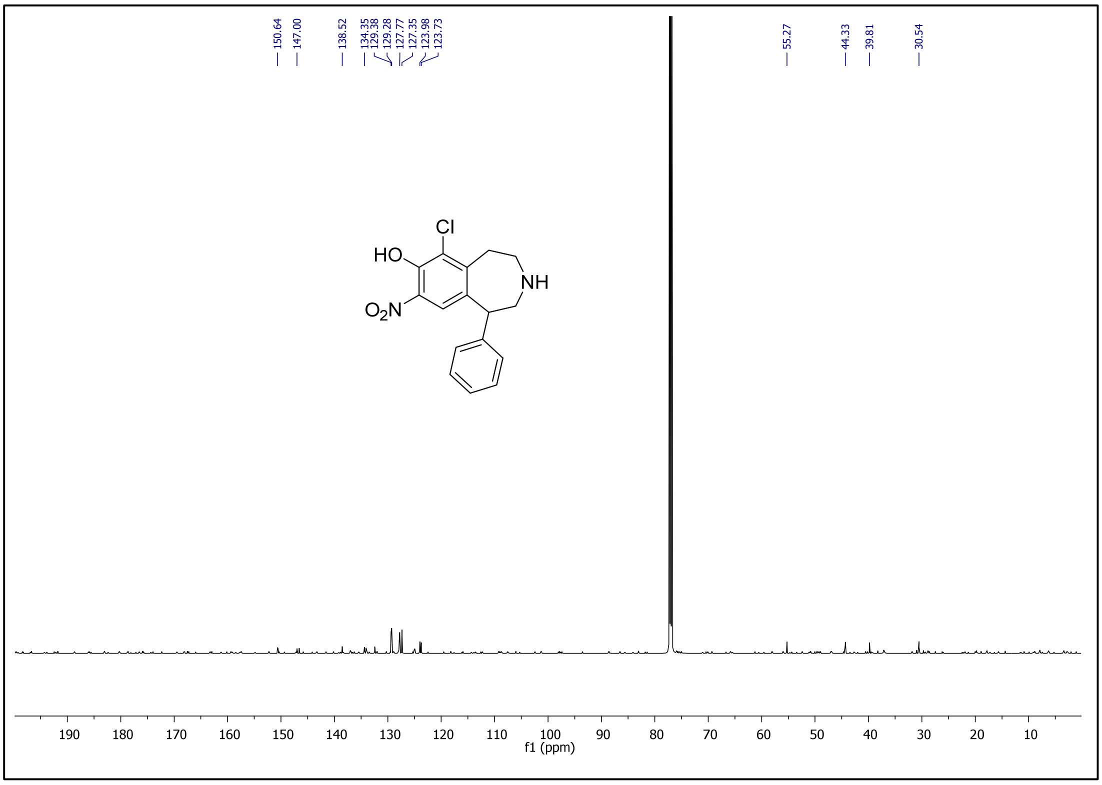
150.64 147.00 138.52 134.35 129.38 129.28 127.77 127.35 123.98 123.73
55.27 44.33 39.81 30.54
Cl
HO
NH
O2N
190 180 170 160 150 140 130 120 110 100 90 80 70 60 50 40 30 20 10
f1 (ppm)

24b

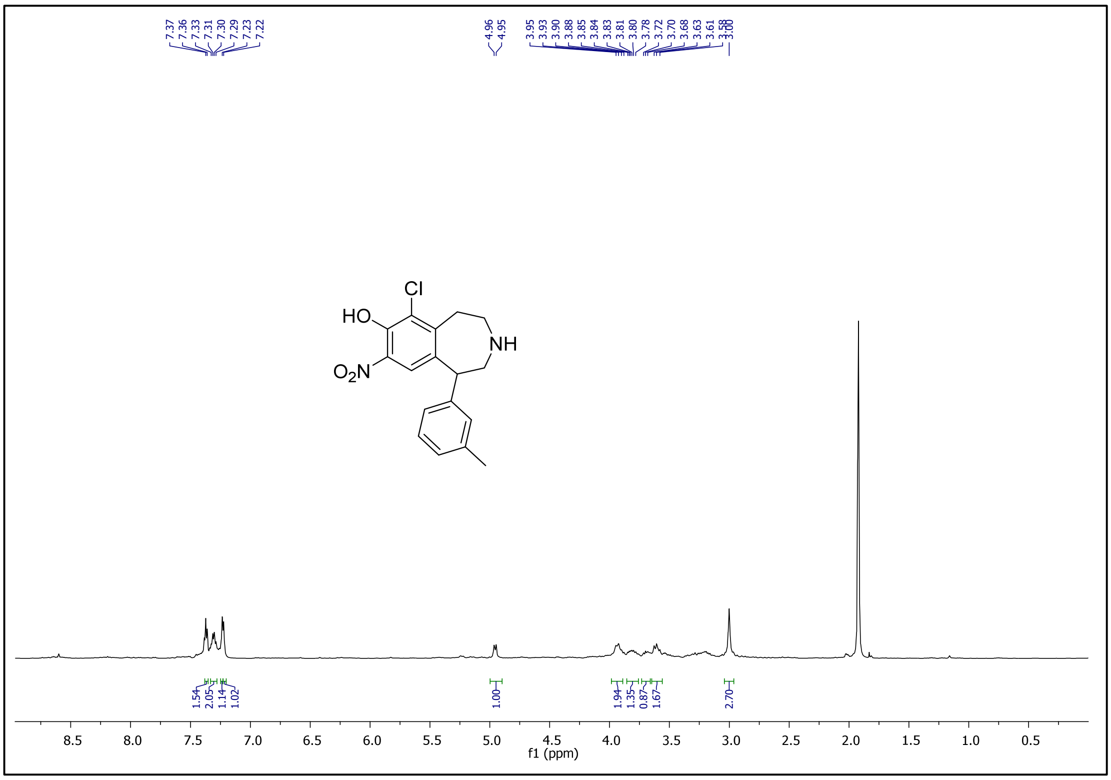

7.37
7.36
7.33
7.31
7.30
7.29
7.23
7.22
4.96
4.95
3.95
3.93
3.90
3.88
3.85
3.84
3.83
3.81
3.80
3.78
3.72
3.70
3.68
3.63
3.61
3.58
3.00
Cl
HO
NH
O2N
1.54
2.05
1.14
1.02
1.00
1.94
1.35
0.87
1.67
2.70
8.5
8.0
7.5
7.0
6.5
6.0
5.5
5.0
4.5
4.0
3.5
3.0
2.5
2.0
1.5
1.0
0.5
f1 (ppm)

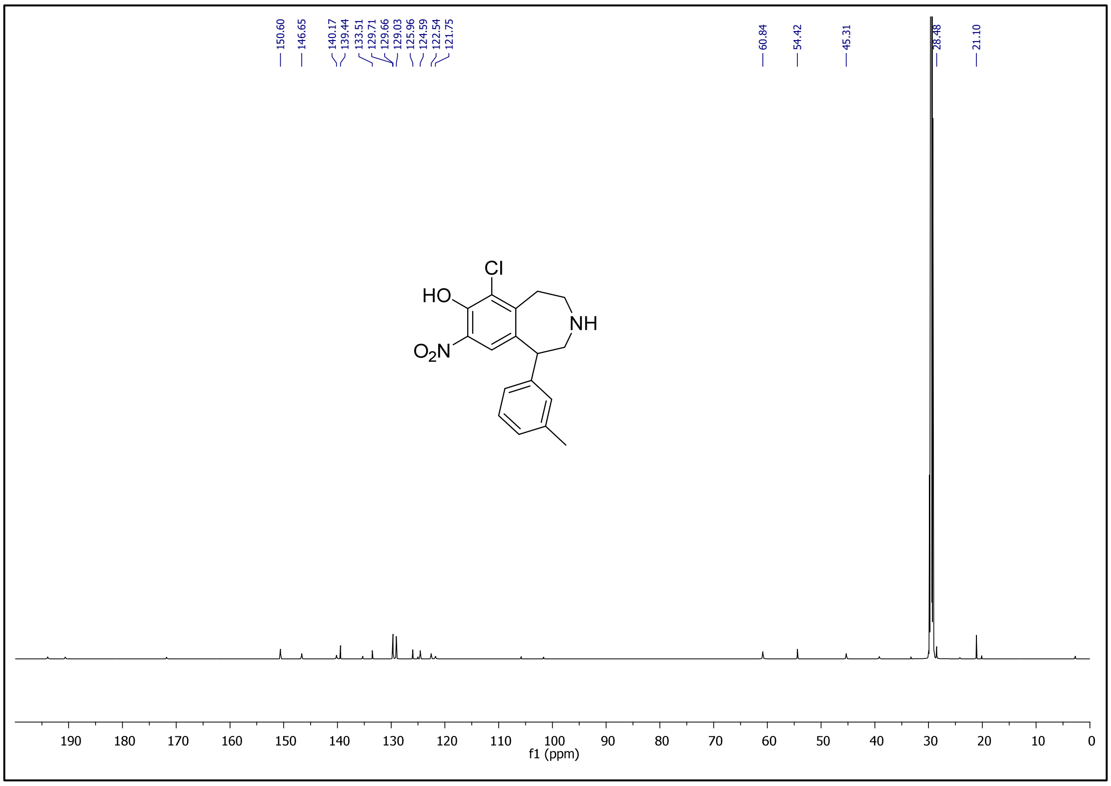

150.60
146.65
140.17
139.44
133.51
129.71
129.66
129.03
125.96
124.59
122.54
121.75
60.84
54.42
45.31
28.48
21.10
Cl
HO
NH
O2N
190
180
170
160
150
140
130
120
110
100
90
80
70
60
50
40
30
20
10
0
f1 (ppm)
